# Comparative Genomics Reveals Specific Genetic Architectures in Nicotine Metabolism of *Pseudomonas* sp. JY-Q

**DOI:** 10.3389/fmicb.2017.02085

**Published:** 2017-10-31

**Authors:** Jun Li, Shulan Qian, Lie Xiong, Chengyun Zhu, Ming Shu, Jie Wang, Yang Jiao, Houlong He, Fuming Zhang, Robert J. Linhardt, Weihong Zhong

**Affiliations:** ^1^Department of Applied Biology, College of Biotechnology and Bioengineering, Zhejiang University of Technology, Hangzhou, China; ^2^Technology Center, China Tobacco Zhejiang Industrial Co., Ltd., Hangzhou, China; ^3^Departments of Chemical and Biological Engineering, Biological Science, Chemistry and Chemical Biology and Biomedical Engineering, Center for Biotechnology and Interdisciplinary Studies, Rensselaer Polytechnic Institute, Troy, NY, United States

**Keywords:** nicotine degradation, *Pseudomonas*, type VI secretion system, tobacco waste extracts, niche advantages

## Abstract

Microbial degradation of nicotine is an important process to control nicotine residues in the aqueous environment. In this study, a high active nicotine degradation strain named *Pseudomonas* sp. JY-Q was isolated from tobacco waste extract (TWE). This strain could completely degrade 5.0 g l^−1^ nicotine in 24 h under optimal culture conditions, and it showed some tolerance even at higher concentrations (10.0 g l^−1^) of nicotine. The complete genome of JY-Q was sequenced to understand the mechanism by which JY-Q could degrade nicotine and tolerate such high nicotine concentrations. Comparative genomic analysis indicated that JY-Q degrades nicotine through putative novel mechanisms. Two candidate gene cluster duplications located separately at distant loci were predicted to be responsible for nicotine degradation. These two nicotine (*Nic*) degradation-related loci (*AA098_21325—AA098_21340*, AA098_03885—AA098_03900) exhibit nearly completely consistent gene organization and component synteny. The nicotinic acid *(NA)* degradation gene cluster (*AA098_17770–AA098_17790*) and *Nic*-like clusters were both predicted to be flanked by mobile genetic elements (MGE). Furthermore, we analyzed the regions of genomic plasticity (RGP) in the JY-Q strain and found a dynamic genome carrying a type VI secretion system (T6SS) that promotes nicotine metabolism and tolerance based on transcriptomics and used *in silico* methods to identify the T6SS effector protein. Thus, a novel nicotine degradation mechanism was elucidated for *Pseudomonas* sp. JY-Q, suggesting its potential application in the bioremediation of nicotine-contaminated environments, such as TWEs.

## Introduction

Nicotine is regarded as toxicant that can cause a variety of adverse environmental effects and preventable diseases. Nicotine degrading microorganisms have been isolated, providing potential approaches in tobacco manufacturing to control nicotine content in final tobacco products and in tobacco waste treatment (Zhong et al., 2010; Liu H. et al., 2015). Nicotine biodegradation has been used in the past to effectively control the nicotine level in tobacco waste extract (TWE) during tobacco processing (Liu H. et al., 2015; Liu J. et al., 2015).

Nicotine biodegradation pathways have been characterized in several microorganisms, including, *Pseudomonas* (Tang et al., 2012) and *Arthrobacter* species (Baitsch et al., 2001). These pathways have been proposed to include the pyrrolidine, pyridine, and a variant of the pyridine and pyrrolidine (Vpp) pathways (Baitsch et al., 2001; Tang et al., 2013; Liu J. et al., 2015; Yu et al., 2015). A pyrrolidine pathway of nicotine degradation was determined to be present in *Pseudomonas*. This pathway involves nicotine oxidoreductase (*NicA)*, pseudooxynicotine amidase (*Pnao)*, 3-succinoylsemialdehyde-pyridne dehydrogenase (*Sapd)*, 3-succinoylpyridine monooxygenase (*SpmABC)*, 6-hydroxy-3-succinoylpyridine hydroxylase (*HspB)*, 2,5-dihydroxypyridine dioxygenase (*Hpo), N*-formylmaleamate deformylase (*Nfo)*, maleamate amidohydrolase, or amidase (*Ami)*, and maleate cis/transisomerase *(Iso*) (Tang et al., 2013). In *Arthrobacter*, nicotine is converted into 2,3,6-trihydroxypyrdine through the pyridine pathway involving the key genes, nicotine dehydrogenase (*Ndh)*, 6-hydroxy-L-nicotine oxidase (6*Hlno*), ketone dehydrogenase (*Kdh)*, 2,6-dihydroxypseudooxynicotine hydrolase (*Ponh)*, and 2,6-dihydroxypyridine 3-hydroxylase (*Dhph)* (Baitsch et al., 2001). A nicotine-degradation associated pathway, the nicotinic acid (NA) degradation pathway, was also demonstrated in *Pseudomonas putida* KT2440 (Jimenez et al., 2008). Recently, a variable hybrid of pyridine and pyrrolidine (Vpp) pathways for nicotine degradation was discovered in several organisms including, *Agrobacterium tumefaciens* S33 (Li et al., 2016), *Shinella* sp. HZN7 (Ma et al., 2014) and *Ochrobactrum* sp. SJY1 (Yu et al., 2015). However, the molecular mechanisms for the nicotine degradation pathways have not been elucidated in most reported bacterial strains. Furthermore, several bacterial catabolic traits or metabolic pathways might result from horizontal gene transfer (HGT) events (Baitsch et al., 2001; Wang et al., 2009; Tang et al., 2012). For example, comparative genomic analyses suggest that mobile genetic elements (MGE) carrying particular traits can provide *Pseudomonas putida* S16 with the capacity to degrade nicotine (Tang et al., 2012). Nevertheless, multi-dimensional approaches are required to decipher either additional novel routes or entire nicotine degradation routes in incompletely known bacteria.

Over the past decade, biochemical activities resulting from the type VI secretion systems (T6SS) in bacteria have generated increasing interest within the research community. It is clear that T6SS can be deployed as a versatile weapon to target/attack other bacteria or impair eukaryotic cells by means of outcompeting rivals in microbial communities. VipA/VipB (also known as TssB/TssC) depolymerization is proposed to assemble long contractile tubes and form phage tail, sheath-like architecture in bacterial cytoplasm (Filloux, 2013). Proteins secreted by bacteria are involved in many important tasks, such as detoxification, drug resistance, and even in key roles in intra-species and inter-species antagonistic interplay. T6SS provides an important way for bacteria to establish niche advantages by delivering a variety of toxic effectors (Durand et al., 2014; Li et al., 2015; Wang et al., 2015; Wan et al., 2017). In addition to the identified conserved components, VgrG also contains flanking regions that might carry secreted effector genes, encoding enzymes, such as lipases and nucleases or other T6SS-relative accessory elements. VgrG1, identified in *Aeromonas hydrophila* SSU, contains a vegetative insecticidal protein (VIP-2) domain with a sequence carrying a carboxyl-terminal extension showing ADP-ribosyltransferase (ADPRT) activity (Filloux, 2013; Durand et al., 2014). T6SS gene clusters often carry less conserved “auxiliary” genes, even ones encoding T6SS specific regulatory factors, effector-immunity protein pairs and secretion chaperones. Paired cognate immunity proteins neutralize effector toxicity, protect self-cellular individuals, and are usually found adjacent to anti-bacterial effectors. Interestingly, additional conserved secreted structural components (namely *hcp* and *vgrG*), outside of the T6SS main clusters termed as Hcp/VgrG islands, function as orphan or auxiliary clusters. “Orphan” *hcp, vgrG*, and adaptor/cargo genes are commonly observed in the proximity of putative effector genes (Filloux, 2013; Durand et al., 2014). To date, however, only a limited number of antibacterial effectors and their corresponding immunity protein pairs have been experimentally identified. We recommend a possible relationship between T6SS and bacterial ability to degrade and tolerate a high concentration of chemicals, such as nicotine in this study.

In the current study, a nicotine-degradation *Pseudomonas* strain was isolated. Its native nicotine-degradation ability is greater than most other reported bacterial strains, particularly its ability to tolerate high concentrations of nicotine. The complete genome of this strain was sequenced to decipher its putative mechanism of nicotine degradation. In-depth data mining for the genetic context of nicotine degradation was performed using comparative genomic analysis. Distantly associated gene clusters, located on the mobile regions of the JY-Q genome, represent different bio-degradation pathways and expression behaviors, and these might originate from other bacterial strains. One particular interesting finding was that T6SS and its secreted effectors may provide variety and complexity to the nicotine-degradation ability of the JY-Q strain. The co-occurrence of designated genetic elements and copy number variation affords diversification required for the high nicotine-degrading performance of *Pseudomonas* sp. JY-Q. These results should facilitate the application of microbial bioremediation in tobacco and tobacco waste processing.

## Materials and methods

### Sample collection and bacterial cultivation

Tobacco waste extract (TWE) samples were obtained from Hangzhou Liqun Environment Protecting Paper Co., Ltd., Hangzhou, China. One bacterial strain named JY-Q showing highly efficient nicotine degradation was isolated from the TWE. Strain JY-Q has been deposited in the China Center for Type Culture Collection (CCTCC No. M2013236).

**Figure 1:**
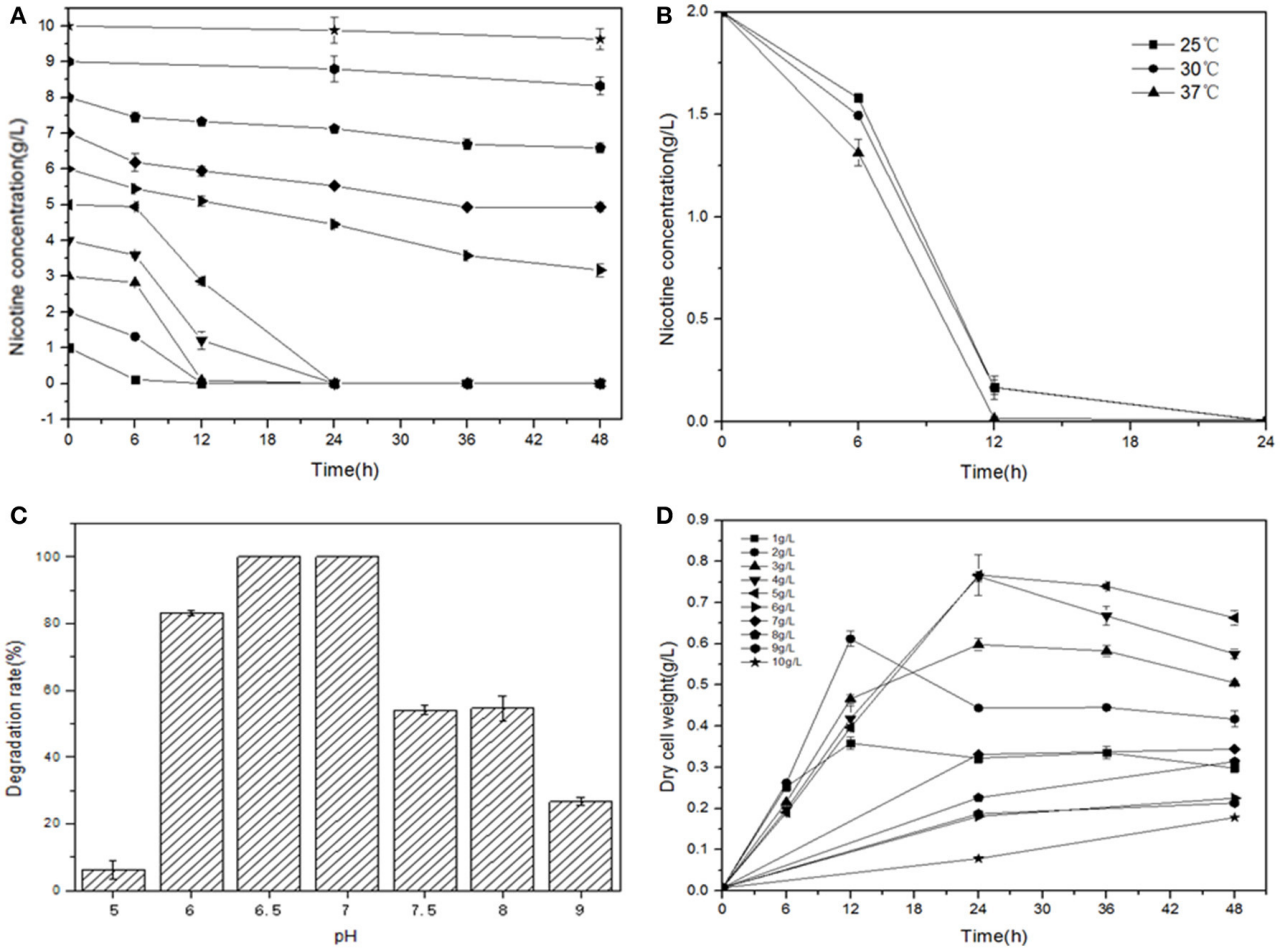
Optimal conditions for bacterial growth and nicotine degradation ability of strain JY-Q. The nicotine concentrations **(A)**, temperatures **(B)** and pH values **(C)** are indicated at the end of each curve. Furthermore, dry cell weight was measured under different nicotine concentrations to estimate the growth and tolerance capacities of strain JY-Q **(D)**. For each sample, three replicates were performed.

L-Nicotine (of 99% purity) was purchased from Fluka Chemie GmbH (Buchs Corp., Switzerland). Filtration-sterilized nicotine was added to basic salt medium (BSM) to prepare nicotine-containing BSM medium. If necessary, adding 1.5% (wt/vol) agar into the liquid medium to prepare for the solid agar plate. Reagents/solvents under study were pertaining to analytical grade and commercially applicable. Meanwhile, different culture temperatures, medium pH values, and additive nicotine concentrations were systemically investigated to acquire the optimal culture conditions of JY-Q (Figure 1).

### DNA sequencing and genomic assembly

The JY-Q was cultivated overnight at 37°C to stationary phase in BSM medium containing additive 2 g l^−1^ nicotine. The total genomic DNA was collected from cells harvested by centrifugation (12,000 × g) at 4°C for 10 min. Genomic DNA (gDNA) in cells was extracted with Qiagen Genomic DNA Preparation Kits and libraries were prepared using the large SMRTbell gDNA protocol (Pacific Biosciences). The genome sequence of JY-Q was first determined using PacBio SMRT, and the HS HGAP *De Novo* Assembler was used to assemble the genomic scaffolds, resulting in 100-fold coverage. Next, 23 large contigs (>500-kb) were acquired by combinational re-sequencing validation by Illumina Hiseq 2000/Mi-Seq (mate paired sequencing with 400-bp library, >100-fold coverage). Finally, gaps and uncertainties, among the obtained contigs, were filled by sequencing of PCR products with Applied Biosystems 3,730 Genetic Analyzer.

### JY-Q sequence annotation and comparative genomics

*Ori*Finder (Luo et al., 2014) was used to predict the replication origin (*ori*C) in JY-Q genome. The gene finding of JY-Q assembled genome were implemented using Prodigal V2.60 (Hyatt et al., 2010) for identification of protein-coding sequences (CDSs), along with careful manual validation of predicted CDS on the basis of annotations of the S16 and ND6 genomes, the ARAGORN (Laslett and Canback, 2004) software for tRNA, and the RNAmmer (Lagesen et al., 2007) program for rRNA. CDS functions were annotated using NCBI-accessible BLASTp searches of NCBI-archived non-redundant (nr) database followed by careful examination of reference annotations of *P. putida* KT2440 (NCBI accession NC_002947) and ND6 (NCBI accession NC_017986).

The putative virulence factor genes were predicted with the BLASTp-based searches against the manually curated VFDB (virulence factor dataset) (Chen et al., 2015). Resistance genes were predicted with ARDB (Liu and Pop, 2009) and CARD (Antimicrobial Resistance Determinant DataBase) (McArthur et al., 2013). Genomic islands (GIs) or regions of genomic plasticity (RGP) harboring aforementioned elements were systemically analyzed and manually determined using the IslandViewer tool [http://pathogenomics.sfu.ca/islandviewer) (Bertelli et al., 2017) and the VRprofile suite (http://bioinfo-mml.sjtu.edu.cn/VRprofile) (Li et al., 2017). The atypical G+C% and integrase-encoding gene features of GIs were also analyzed in-depth. GI-carrying gene clusters were comparatively described using the available standalone program MultiGeneBlast (http://multigeneblast.sourceforge.net/) (Medema et al., 2013). Assembled and partially sequenced bacterial genomic GenBank-formatted information was used in the customized search database for the supplied *Nic*-like gene cluster sequences with no reliance on any pre-calculated resources.

Genomic level comparisons between JY-Q with NCBI-archived 9 completely assembled *Pseudomonas putida* genomes were performed using the BLAST-based rapid genome alignment tool–online “mGenomeSubtractor” (Shao et al., 2010). In that, *H*-value (0–1.0) was used as cut-off to reflect the similarity degree between two matching homologs. JY-Q annotated protein-coding genes were served as queries to be comparatively examined by mGenomeSubtractor-facilitated BLASTn-optional analyses against other selected *Pseudomonas putida* genomes (in this study as subject), together with conserved genes definition using embedded *H* > 0.42 (identities >70, matching length coverage >60%).

Insertion sequence (IS) elements can mediate lateral gene transfer to influence the instability of the bacterial genome. ISs were mainly identified with ISsaga and ISfinder (Varani et al., 2011) and then manually curated with focuses on intactness, terminal inverse repeats (IRs) and flanking direct repeats (DRs). Prophages were predicted with PHAST (Zhou et al., 2011) and *Phage_Finder* (Fouts, 2006), and cognate boundaries were systemically checked. The 16S rRNA and proteome phylogenetics tree was inferred by PhyML (Guindon et al., 2010) and CVtree (Xu and Hao, 2009) with the maximum likelihood and *K*-string composition methods, respectively, showing that individual genome of strain JY-Q was more closely related to *Pseudomonas putida* ND6 than to S16.

### Nicotine-degradation related gene clusters identification

**Figure 2:**
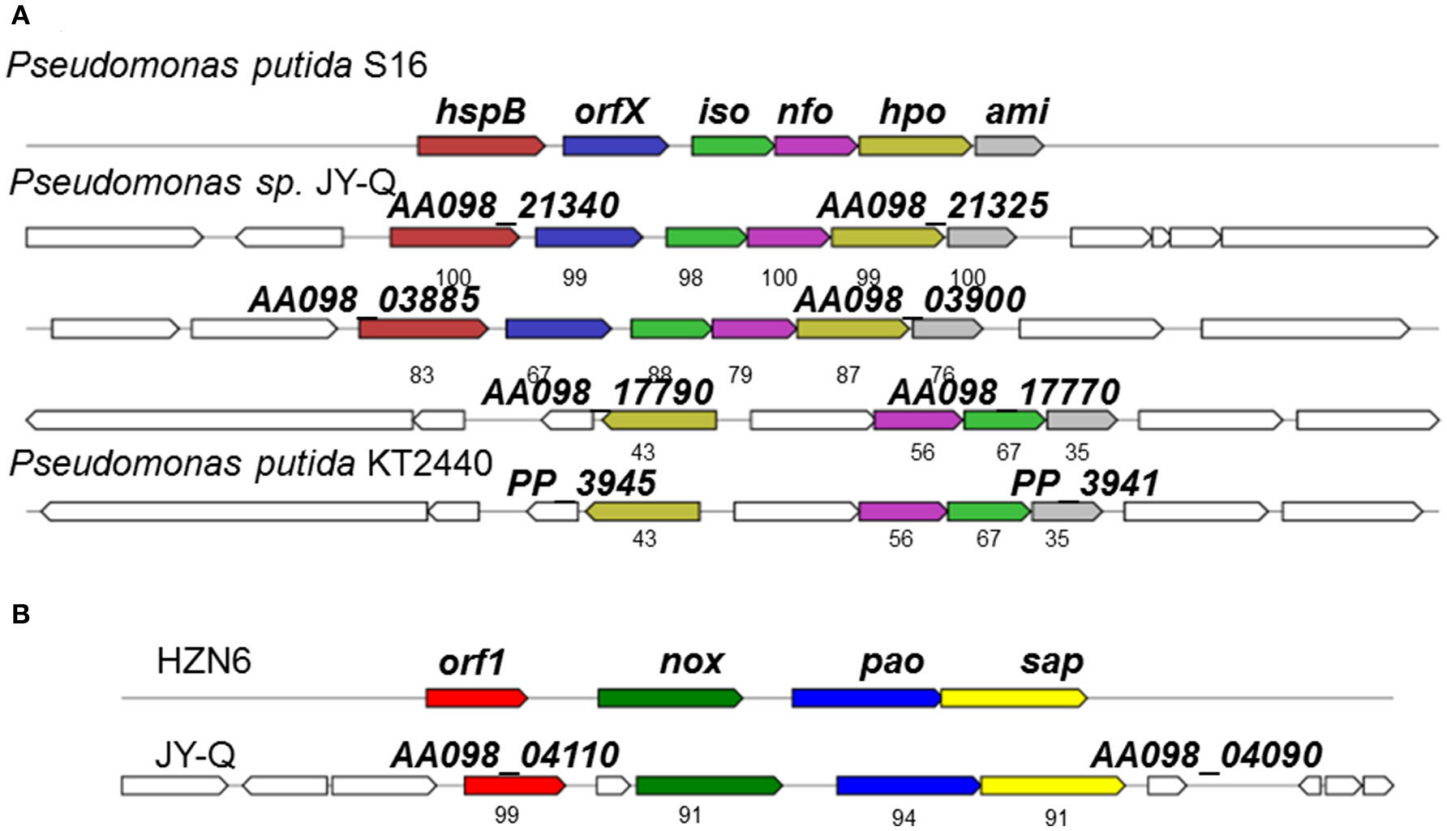
Schematic representation for genetic organization and immediate vicinity of putative nicotine-degradation gene clusters of *Pseudomonas* sp. JY-Q compared with the similar gene cluster from *Pseudomonas putida* S16, KT2440 **(A)** and HZN6 **(B)**. *HspB*, HSP hydroxylase; *Iso*, maleate isomerase; *Nfo*, NFM deformylase; *Hpo*, DHP dioxygenase; *Ami*, maleamate amidase; *Hna*, 6-hydroxynicotine 3-monooxygenase; *Nox*, nicotine oxidase; *Pao*, pseudooxynicotine amine oxidase; *Sap*, NADP^+^-dependent 3-succinoylsemialdehyde-pyridine dehydrogenase; *Orf*, no predicted function. The numbers within the arrows indicate the percent amino acid sequence identity with the orthologous gene product from *Pseudomonas putida* S16 and sp. HZN6.

Fragments containing the *Nic*-like gene clusters in strain JY-Q, involved in the nicotine-degradation, were identified through literature curation and data mining for gene clusters and cognate protein sequences of *P. putida* KT2440 and S16. Two *Nic* homology gene clusters and one *NA*-like genetic locus were characterized by *in silico* analyses (Figure 2). Several homologous genetic loci similar to *Nic* in *P. putida* S16 were also identified in the customized *Pseudomonas* genome databases, as exemplified in Figure 2. The *Nic*-like gene clusters discovered generally encoded five enzymes that could step-by-step convert 6-hydroxy-3-succinoylpyridine (HSP) to fumaric acid, formic acid and ammonia.

### Intermediates analysis in nicotine degradation with GC-MS

Strain JY-Q was firstly cultivated in 250-ml flasks containing 100 ml sterilized BSM with an initial content of 2.0 g l^−1^ nicotine at 30°C, 180 rpm. After 6, 12, and 24 h of independent cultivation, samples were treated with benzene to extract metabolites for 10 min (4°C), centrifugation up to 10 min (12,000 × g), and then obtain the resulting supernatant prior to GC-MS analyses. Intermediates of nicotine degradation were measured and examined by GC-MS (Agilent 5975C) equipped with electron impact ionization (EI) sources. The dynamic phase was helium (He gas) at a flow rate of 1.0 ml min^−1^. GC-MS analysis was performed with temperature program (the GC oven was initially set with 60°C for 2 min, then to 280°C at a rate of 10°C min^−1^). This was followed by maintaining for 10 min at 280°C. The ion source temperature was 250°C and the interface temperature was 270°C. The quadrupole temperature was held constant at 250°C. The relative peak abundance indicated the ion number. Data acquisition processes consisted of material identification, metabolite quantification, peak merging, and also included retention time and absorption spectra that were compared with those of back-end, database-archived, standard compounds for full characterization of each run.

### Nucleotide sequence accession number

The 16S rRNA and *Pseudomonas* sp. JY-Q genomic nucleotide sequences under study have been submitted into GenBank with NCBI accession number KC963965 and CP011525, respectively.

## Results

### Remarkable nicotine-degradation and tolerance of *Pseudomonas* sp. JY-Q

The JY-Q strain was incubated in 250 ml flasks with 100 ml sterilized BSM containing 2.0 g l^−1^ nicotine at 180 rpm and 30°C. Nicotine tolerance of the JY-Q strain was evaluated at initial nicotine concentrations ranging from 1.0 to 10.0 g l^−1^ under defined optimal cultivation temperature and pH (Figure 1). In Figure 1A, the JY-Q strain can grow well at 10 g/L of nicotine (the sole carbon and nitrogen source). Meanwhile we detected the nicotine concentration decreases from 10 to 9.632 g/L (48 h), which is sufficient for the survival of JY-Q. We also detected bacterial growth from zero to 0.178 g/L (48 h) at 10 g/L of nicotine (Figures 1A,D). After cultivation for 24 h, the cells harvested from 2 ml broth were inoculated into 100 ml fresh BSM supplemented with 2.0 g l^−1^ nicotine and incubated at 25, 30, and 37°C, respectively (Figure 1B). BSM media at pH of 5.0, 6.0, 6.5, 7.0, 7.5, 8.0, and 9.0 was used to evaluate pH effect on JY-Q growth (Figure 1C, most suitable pH condition: 6.5–7.0). As shown in Figures 1B–C, and from statistical data, the most efficient temperature for nicotine degradation is the 37°C.

Both JY-Q strain growth and nicotine degradation could be detected in the medium at a maximum nicotine concentration at 10.0 g l^−1^ at an optimal pH of 6.5–7.0 and an optimal temperature of 37°C. The JY-Q strain completely degraded up to 5.0 g l^−1^ nicotine within 24 h (Table 1). However, both the degradation behavior and growth of the JY-Q strain were inhibited at nicotine concentrations of 10.0 g l^−1^ of higher in BSM medium. The nicotine concentration in TWE reaches 20 g/L so that the improvement of the JY-Q strain nicotine tolerance will be necessary.

**Table 1:** Comparison of nicotine degradation ability of reported bacterial strains.

**Bacterial strain**	**Source**	**Optimal conditions**	**Degradation efficiency**	**Tolerance level**	**References**
*Shinella* sp. HZN7	Activated sludge	30°C, pH 7.0	3.0 g l^−1^, 13 h	5.0 g l^−1^	Ma et al., 2014
*Arthrobacter* sp. HF-2	Waste-contaminated soil	30°C, pH 7.0	0.7 g l^−1^, 43 h	2.0 g l^−1^	Ruan et al., 2006
*Arthrobacter oxidans* pAO1	−^a^	–	3.4 g l^−1^, 125 h	–	Baitsch et al., 2001
*Achromobacter nicotinophagum*	Tobacco seed and soil	20–25°C, pH 6.8–7.0	1.62 g l^−1^, 56 h	–	Hylin, 1959
*Ensifer* sp. N7	Tobacco soil	28°C, pH 7.0	3.35 g l^−1^, 48 h	4.0 g l^−1^	Liu J. et al., 2015
*Arthrobacter* sp. M2012083	Tobacco waste	–	–	–	Yao et al., 2015
*Ochrobactrum* sp. SJY1	Nicotine contaminated water	30°C, pH 7.0	4.0 g l^−1^, 10 h	4.0 g l^−1^	Yu et al., 2015
*Acinetobacter* sp. TW	Tobacco waste	30°C, pH 7.0	1.0 g l^−1^, 12 h	2.53 g l^−1^	Wang et al., 2013
*Agrobacterium tumefaciens* S33	Tobacco soil	30°C, pH 7.0	1.0 g l^−1^, 6 h	5.0 g l^−1^	Wang et al., 2012
*Pseudomonas* sp. HF-1	Tobacco waste	30°C, pH 6.5−7.5	1.3 g l^−1^, 25 h	1.6 g l^−1^	Ruan et al., 2005
*Pseudomonas* sp. ZUTSKD	Waste tobacco leaf	30°C, pH 7.0	1.55 g l^−1^, 12 h	5.8 g l^−1^	Zhong et al., 2010
*Pseudomonas plecoglossicida* TND35	Tobacco soil	30°C, pH 7.0	3.0 g l^−1^, 12 h	5.0 g l^−1^	Raman et al., 2014
*Pseudomonas stutzeri* ZCJ	Tobacco leaf	37°C, pH 7.4	1.5 g l^−1^, 24 h	4.5 g l^−1^	Zhao et al., 2012
*Pseudomonas* sp. HZN6	Activated sludge	30°C, pH 7.0	0.5 g l^−1^, 12 h	–	Qiu et al., 2013
*Pseudomonas putida* S16	Tobacco soil	30°C, pH 7.0	3.0 g l^−1^, 10 h	6.0 g l^−1^	Wang et al., 2004
*Pseudomonas* sp. JY-Q	Tobacco waste extract^b^	37°C, pH 6.5−7.0	3.0 g l^−1^, 12 h 5.0 g l^−1^, 24 h	10.0 g l^−1^	Current study

a *“−”, not available*.

b *Optimal conditions similar to industrial waste properties form the tobacco and cigarette manufactures in nature*.

The 16S rRNA gene of the JY-Q strain was sequenced and deposited (NCBI GenBank, accession number: KC963965). BLASTn-based searches of GenBank (Benson et al., 2015), as well as Ribosomal Database Project library (RDP, http://rdp.cme.msu.edu/) (Cole et al., 2007) indicated that the JY-Q 16S rRNA gene exhibited high sequence identity (>99%) with reported *Pseudomonas putida* isolates.

The native nicotine-degradation ability of *Pseudomonas* sp. JY-Q was greater than most reported bacterial strains, and in particular it was more tolerant of concentrations of nicotine higher than all other bacterial strains (Table 1). Therefore, we decided to obtain genome of strain JY-Q using a combination of PacBio SMRT technologies, Illumina Hiseq 2000/Mi-Seq platforms, and filling or clarifying genomic gaps and uncertainties with PCR sequencing. Our sequencing results showed that the JY-Q genome was composed of a single circular replicon (main chromosome) with 6,178,825 base pairs. The final contig was checked for circularization and overlapping ends were trimmed. The JY-Q genomic features (Table 2), were compared with those of distantly related nicotine degradation strain *Pseudomonas putida* S16 and closely related naphthalene degradation strain *Pseudomonas putida* ND6 (Supplementary Figure S1). *Ori*Finder was employed to predict the replication origin (*ori*C) in JY-Q genome (Luo et al., 2014). The assembled genome sequences were annotated using the SecReT6-integrated CDSeasy (Li et al., 2015) suite, which included Prodigal (Hyatt et al., 2010) and Representative Bacterial Proteomes (Chen et al., 2011) for the identification of protein-coding sequences, ARAGORN (Laslett and Canback, 2004) for tRNA, and RNAmmer (Lagesen et al., 2007) for rRNA. This was followed by manual inspection using RefSeq assigned annotation. Comparison at the bacterial genomic sequence level relied on the Prokaryotic Genome Annotation Server (RAST, Rapid Annotation Server, and Tools) (Overbeek et al., 2014) and inferred phylogeny deployed CVTree (Xu and Hao, 2009). Both methods showed that the genomic sequence of strain JY-Q closely matched those of sequenced *Pseudomonas putida* strains (Supplementary Figure S1).

**Table 2:** General features of the *Pseudomonas* sp. JY-Q, the closely related strain ND6 and nicotine-degrading strain S16 genomes.

**Features**	**JY-Q**	**S16**	**ND6**
Replicon	1 chromosome	1 chromosome	1 chromosome, 1 plasmid (pND6-2)
Size (Mb)	6.17	5.98	6.09, 0.11
G+C%	61.3	62.3	61.8, 57.8
ORFs	5334	5243	5305, 121
rRNA	22	19	19
tRNA	82	67	76
Note	nicotine-degrading	nicotine-degrading	naphthalene-degrading

### Unexpected co-occurrence of three nicotine degradation-associated gene clusters in JY-Q

The *Pseudomonas* sp. JY-Q genome is composed of a single circular chromosome (6,178,825 bp in length) without any plasmids (Table 2). Pan-genome analyses were performed to provide an overview of conserved gene categorization, and strain-specific genomic groups. JY-Q genomic protein-coding sequences (CDSs) were comparatively analyzed by mGenomeSubtractor-based (BLASTn option) *in silico* subtractive hybridization (Shao et al., 2010) for presence/absence of homologs against nine GenBank-archived completely sequenced *Pseudomonas putida* genomes. In particular, comparative genomics showed 2823 genes homologous (*H* > 0.42) to these *P. putida* genomes (Supplementary Figure S2A), representing 52.9% of the total CDSs in JY-Q. The JY-Q genome was analyzed in a continuous linear bar format (100-Kb scale for each rectangle bar) with a degree of “blackness/whiteness” reflecting the relative conservation/diversity (Supplementary Figure S2B). The JY-Q genome is similar to *Pseudomonas* phylogenetics (Supplementary Figure S1) and exhibits the highest level of sequence identities to the ND6 genome (4,522 conserved genes defined with *H* > 0.42 as cut-off) (Supplementary Figure S2B). Non-conserved genes between strain JY-Q and another nicotine degradation *Pseudomonas putida* S16 are shown in Supplementary Table S1. Comparative general features of the JY-Q, ND6, and S16 genomes are shown in Table 2. Secondary metabolism gene clusters, IS elements and prophages located on the JY-Q chromosome are listed in Supplementary Tables S2–S4. MGEs and other genetic elements (such as T6SS shown in Supplementary Figure S3) were computationally identified using a designed bacterial annotation procedure (see the “Materials and Method” section).

A total of three putative nicotine-degradation associated gene clusters were identified, one referred to as *NA* (NA degrading gene cluster, flagged in Supplementary Figure S2B using “red box”) and another two referred to as *Nic*-like (nicotine degrading gene cluster, flagged in Supplementary Figure S2B using “navy box”) (Figure 2A). Comparative and phylogenetic analyses of the respective gene clusters with genetic contexts indicate their distant evolutionary origins and their different catabolic routes. The HZN6-like gene cluster clearly showed that initial nicotine degradation steps might be catalyzed by three consecutive enzymes encoded by *nox, pao*, and *sap* (Figure 2B; Qiu et al., 2013). In contrast to an average genomic G+C content of 61.3%, the G+C contents of the *NA*-like (*AA098_17770*–*AA098_17790*) and *Nic*-like (*AA098_21325*–*AA098_21340, AA098_03885*–*AA098_03900*) clusters in *Pseudomonas* sp. JY-Q are approximately 63.6, 47.9, and 50.5%, respectively, (Figure 2 and Supplementary Figure S2B). Interestingly, *AA098_21420* and *AA098_2143*5, respectively encoding Pnao and NicA2, crucial for nicotine degradation, are involved in the genomic contexts of integrase genes *AA098_21425* and *AA098_2143*0 (*Tra8* derivatives) implicated in horizontal genetic transfer potential. In contrast, no insertion was observed between *pnao* and *NicA2* in strain S16 (locus tag: *PPS_4080, PPS_4081*).

### Intermediate metabolites identification indicating existence of novel nicotine degradation pathways

Different initial reactions on the pyrrolidine ring can result in different sequential processes. Gas chromatography-mass spectrometry (GC-MS) was used to investigate the nicotine degradation intermetabolites and profile strain JY-Q in response to added nicotine to identify the functional pathways. We harvested and explored the resulting accumulating metabolites. Representative total ion current chromatograms of strain JY-Q were determined and the relative abundance of the categorized detected metabolites was determined (Supplementary Figure S4). Metabolomic analysis of nicotine degradation intermediates differed from known pyrrolidine, pyridine, and *Vpp* pathways (except for intermediates of the intact pyrrolidine pathway found in JY-Q isolate), indicating a novel mechanism for nicotine bioconversion (Supplementary Figure S4). A variety of detected intermediates (Supplementary Figure S4A), including, 3-(3,4-dihydro-2H-pyrrol-5-yl)-pyridine, 12.353 min; nicotryine, 13.097 min; and 2,3′-bipyridine, 13.713 min), had also been previously reported as nicotine metabolic products of *Pseudomonas sp*. ZUTSKD (Zhong et al., 2010), *Pseudomonas* sp. HF-1 (Wang et al., 2009), and *Shinella* sp. HZN1 (Jiang et al., 2011). The results obtained suggest an increased metabolite diversity in response to nicotine exposure and this observation requires additional in-depth investigation. GC-MS analytical results are shown in Supplementary Figure S4B and in the “Materials and Method” section.

### Genomic analysis indicates a plausible association between JY-Q mobilome and metabolism potential

In light of the metabolic diversity and remarkable nicotine tolerance of *Pseudomonas* sp. JY-Q, a secondary metabolic gene cluster candidate and other genetic features were investigated. The potential virulence factor loci and antimicrobial susceptibility profile was determined using the VRprofile pipeline (available at http://bioinfo-mml.sjtu.edu.cn/VRprofile) (Li et al., 2017) to explore the industrial and experimental application of strain JY-Q. This afforded a deeper understanding of genomic organization and genetic determinants. In addition to identifying the loci on single gene scale, flagellar, O-antigen biosynthesis, other virulence associated gene clusters within the genome of strain JY-Q were screened (Supplementary Table S2). However, in JY-Q, virulence factors, such as exotoxins and type III secretion systems were absent. Remarkably, *AA098_15435* was a homolog coding for fluoroquinolone/chloramphenicol multi-drug resistance efflux pump. *AA098_22905* was predicted to be involved in acriflavine resistance. These findings suggest another possibility for high nicotine resistance of JY-Q. The secondary metabolic product synthesis genetic loci were discriminated by antiSMASH suites (http://antismash.secondarymetabolites.org/) (Blin et al., 2017). Adjacent genes were merged into the identified PKS/NRPS gene cluster, when existing functional annotation was specific to known genes. We then refined the genetic loci through manual curation (Supplementary Table S2 and Supplementary Figure S2).

**Figure 3:**
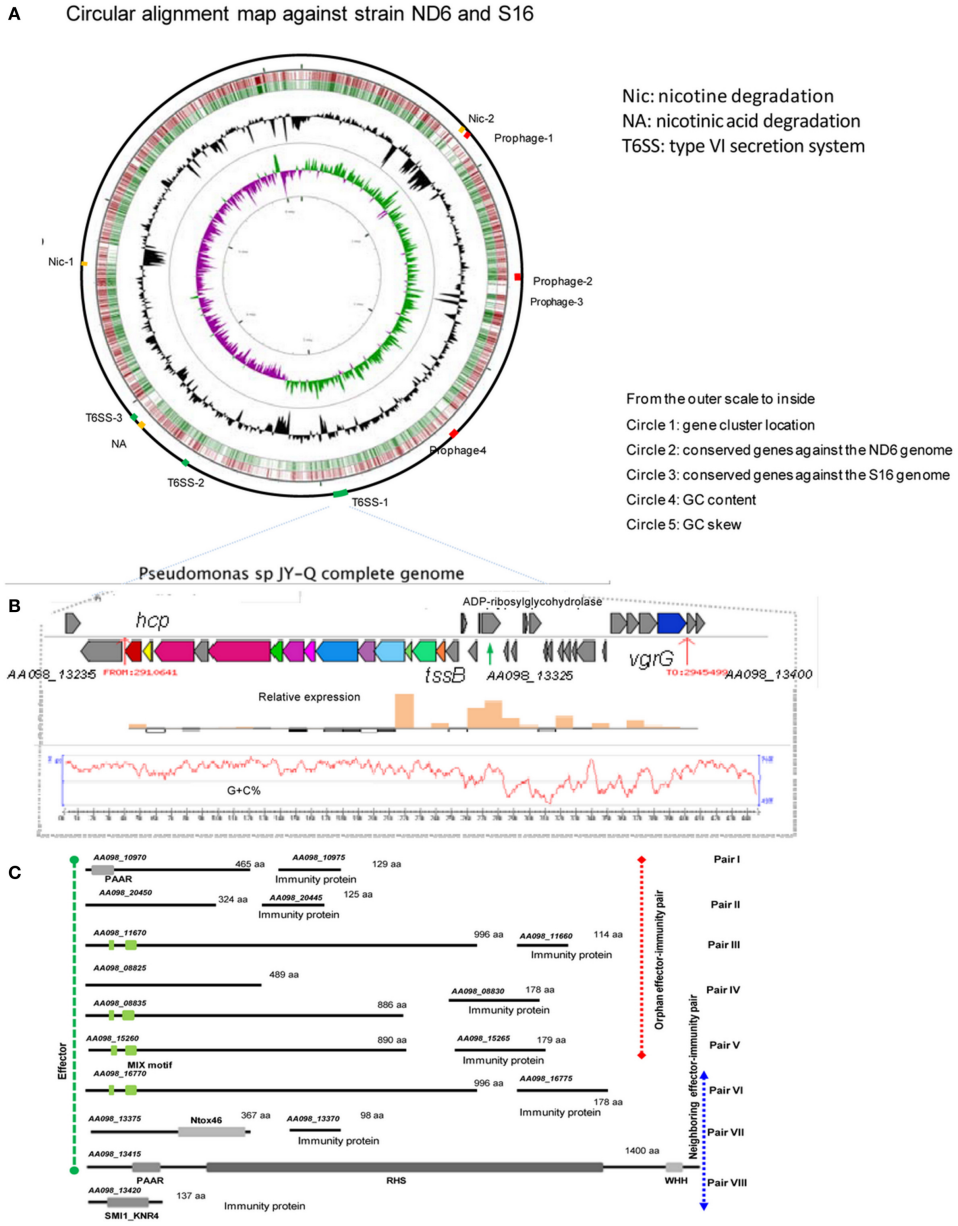
Identified T6SS gene clusters and corresponding effector-immunity protein pairs in JY-Q. **(A)** T6SS and nicotine metabolism related gene cluster genomic organization, **(B)** as well as schematics of T6SS-1 gene cluster relative expression in the JY-Q genome via transcriptomics**. (C)** Inferred T6SS related effector-immunity protein candidates faraway from (orphan pairs) or close to (neighboring pairs) T6SS gene clusters by means of comparative genomics and chaperon co-occurrence feasibility, with conserved domains drawn to scale. MIX, marker for type six effectors; WHH, a predicted nuclease of the HNH/ENDO VII superfamily; PAAR, PAAR_motif; Ntox46, bacterial toxin 46; SM1_KNR4, primary immunity proteins in bacterial toxin system.

As canonical agents for horizontal gene transfer, IS elements can cause other genetic modification resulting in a decrease of bacterial genome stability, such as genomic rearrangements, inversions, and deletions. There were a variety of IS*Pa41* and IS*Psp7* elements in the vicinity of JY-Q *Nic-like* gene clusters, suggesting a possible instability in the JY-Q genome in TWE specific niches (Supplementary Table S3). The HZN6-like gene cluster of JY-Q was also observed flanked by IS*Psp7* (AA098_04115 as transposase) (Supplementary Table S3 and Supplementary Figure S2). Four prophage-like regions were also identified in the JY-Q genome (Supplementary Table S4), together with other MGEs (Figure 3A) benefiting survival and bio-degradation activities in TWE niches.

### Distantly phylogenetic related type VI secretion gene cluster and effector protein prediction

First, three T6SS gene clusters (*AA098_13235*–*AA098_13300, AA098_16690*–*AA098_16760*, and *AA098_18490*–*AA098_18555*) were detected in the JY-Q genome by employing T6SS-HMMER in SecReT6 (Li et al., 2015) (referred to respectively as T6SS-1, T6SS-2, T6SS-3, and Supplementary Figure S3). Furthermore, composition difference and variable regions amongst T6SS clusters imply their distinctive phylogeny (Supplementary Figure S3: gene cluster alignment for T6SS-1 against T6SS-2 and T6SS-3).

Subsequent transcriptomic analysis of JY-Q in BSM with and without nicotine was next carried out. Surprisingly, we found that T6SS-1 expression was significantly induced by nicotine (Figure 3B), in contrast to T6SS-2 and T6SS-3 remained unchanged. Additional nicotine stimulated TssB-TssC and variable region expression. We speculate that these are associated with T6SS-1 contractile architecture and accessory modules.

In general, genes encoding T6SS effectors and immunity proteins are usually co-localized in genomic variable regions. Therefore, we exhaustively searched the putative effector proteins using self-customized heuristic approaches from previous reports including: (1) candidate co-occurrence of chaperone (Pfam entries: PAAR, DUF1795, DUF4123, DUF2169) and effectors; (2) evolved structural VgrG C-terminus effector domain; (3) orphan Hcp/VgrG island vicinity; (4) multiple gene cluster alignment for the variable regions (Filloux, 2013; Durand et al., 2014).

As the first result, ADP-ribosyltransferase (Gene locus tag: *AA098_13325*) and Ntox46-superfamily toxin (Gene locus tag: *AA098_13375*) were predicted to be T6SS effector candidates located within a 13.1-Kb large variable/auxiliary genomic region (Figures 3B,C and Supplementary Figure S3). A SMI1_KNR4-domain carrying protein (Gene locus tag: *AA098_13420*) suspected to be a primary immunity protein against the PAAR-RHS-WHH nuclease toxin (Gene locus tag: *AA098_13415*) was determined in the vicinity of DUF1795 (Gene locus tag: *AA098_13405* and *AA098_13410*). Four predicted MIX motif carrying effectors and paired immunity proteins were also defined, including *AA098_15260* (Figure 3C, candidate adaptors listed in Supplementary Table S5). Additional orphan effector-immunity protein pairs were *in silico* characterized through multiple comparisons of self-customized *Pseudomonas putida* genomes. In summary, eight pairs of T6SS effector-immunity determinants were identified inside and outside of T6SS gene clusters (Figure 3C) that might play roles on JY-Q capacity of surviving in high nicotine concentrations.

## Discussion

Specific genetic patterns in strain JY-Q were found by using comparative genomics, and these may be responsible for its nicotine tolerance and metabolism ability. Based on our prediction, the relatively different G+C content, genetic contexts of the JY-Q *Nic*- and *NA-*like gene clusters and the divergence of the amino acid sequences suggested that *Nic*-like gene clusters of *P. putida* JY-Q might be horizontally transferred from other bacterial strains (Figure 2 and Supplementary Figure S2B). Likewise, the *Nic-*like gene cluster has been found to be located in the largest RGP in strain S16 (Tang et al., 2012, 2013). In addition, strain KT2440 that contains the *nox*-carrying (HZN6-like gene cluster shown in Figure 2B) plasmid that can completely degrade nicotine (Qiu et al., 2013), whereas wild-type strain KT2440 is a non-nicotine-degrading bacterium. Furthermore, *NA*-like genes, in the NA degradation pathway of strain JY-Q (Figure 2A), were also characterized in the genome of the closely related strain, ND6, but absent in the S16 genome (no nicotine degradation ability is present in the ND6 of KT2440 strains). These genetic findings provide us with additional evidence for the biochemical diversity in the nicotine bioconversion pathways of the *Pseudomonas* genus, particularly in strain JY-Q. Interestingly, three putative gene clusters are co-located within three RGPs in the JY-Q genome (Figure 3A, Supplementary Figure S2). Gene sequence alignment shows partial synteny and variation (30–100% amino acid sequence identities) from the closest homology nicotine degrading gene cluster in *P. putida* S16. The co-occurrence and copy-number/sequence-composition variation of the distinct gene clusters might assist understanding the high efficiency of nicotine biotransformation mediated by strain JY-Q. The differences in the genetic composition and enzymatic components of the *NA*-like and *Nic-*like gene clusters also indicate that the nicotine and NA degradation genes evolved independently. Moreover, the three distinctly related and intact T6SS gene clusters co-occurring in the JY-Q genome is idiosyncratic, rather than functionally redundant. The T6SS core component TssB-based phylogenetics of T6SS is not fully related to bacterial taxonomy (Li et al., 2015) and together with T6SS is observed to be frequently localized onto GIs, implying that the T6SS gene clusters may be obtained from HGT events (Ho et al., 2013; Durand et al., 2014; Borgeaud et al., 2015). Surprisingly, such co-occurrence of identified gene clusters in the genome of strain JY-Q have not been previously identified in any other NCBI-deposited *Pseudomonas* genomes.

We also found T6SS expression to be implicated in nicotine tolerance during degradative metabolism. Since T6SS is related to bacterial niche resistance/adaption and quorum sensing, we suggest that T6SS machinery is associated with the striking nicotine tolerance exhibited by strain JY-Q. Bacterial T6SSs are a sophisticated apparatus that mediate antagonistic or symbiotic interplay between bacteria and/or bacteria and eukaryotic cells (Li et al., 2015). Their expression in strain JY-Q was shown by RNA-Seq to respond to changes in nicotine level. T6SS from different species might assist bacterial multiple resistance/tolerance against comparable enriched Reactive Oxygen Species (ROS), and/or osmotic pressure (Filloux, 2013). Despite known T6SS-mediated anti-bacterial and anti-eukaryotic traits, newly-emerging T6SS impacts include bacterial survival/colonization and niche resistance/fitness, however still needs to be linked to specific effectors. A better understanding on the determination of “real-life” significance of T6SS and corresponding effector proteins in different biological contexts require further research.

The relationship between T6SS and nicotine metabolism in strain JY-Q will need to be deciphered in the future. The T6SS apparatus and effector proteins have been proposed to play key roles in infection/pathogenicity, symbiosis/competitive adaption capacities, survival/growth opportunities within specific hosts and escape from macrophage cell predation. The characterization of secretion (targeting) sequence features are required to define T6SS-exported effectors remain a major research obstacle. It has recently been reported that YezP (Wang et al., 2015), TseM (Si et al., 2017), and TseF (Lin et al., 2017) are T6SS extracellular effectors attributed to metal ion translocation and ROS balance (oxidative stress and environmental stress resistance). Thus, identification of functional effectors is required for a better understanding of T6SS facilitated inter-bacterial or bacteria-host dynamics. We relied on four strategies to predict T6SS effectors through customized informatics models. Moreover, comparative genomic methods used in the current study have predicted a variety of putative secreted effector candidates delivered by T6SSs. Additional secreted proteins, harboring unknown effector domains, still remain to be characterized.

## Conclusion

A unique bacterium *Pseudomonas* sp. JY-Q capable capable of degrading nicotine from TWE was isolated and its genome was sequenced. Compared to other published strains, strain JY-Q was more resistant to nicotine and nicotine-containing agents. In this study, experimental techniques and comparative genomics provided insights into the nicotine degradation processes in this important bacterium. This *Pseudomonas* species, isolated from TWE, exhibited efficient nicotine degradation capabilities, however, the precise biodegradation mechanisms relied by this JY-Q isolate still remain further clarification.

Interestingly, the JY-Q genome contained a large number of specific auxiliary functional RGPs that were significantly different from all other sequenced industrial isolates. These included, multiple duplication of nicotine degradation (*NA*- and *Nic*-like) and T6SS gene clusters, co-localized onto the “mobilome.” Bacterial resistance against an extreme niche change represents a fantastic T6SS-mediating activity, extending our knowledge of known pathogenic virulence and anti-bacterial strategies. T6SS activities have been attributed to resistance to osmotic pressure changes, pH conditions, membrane signal transduction, oxidative stresses. Thus, T6SS response to enhance survival and/or nicotine degradation needs clarification. Here we also report strain JY-Q T6SS associated effector-immunity protein pairs, representing a self-customized comparative genomic pipeline.

The comparative genomics and identified phylogeny of strain JY-Q genome with other *Pseudomonas* representatives suggests novel nicotine degradation pathway or complex interplay of different pathways. However, details on the entire mechanism of nicotine degradation and nicotine tolerance require to be elucidated. The TWE originated strain, *Pseudomonas* sp. JY-Q, has evolved its specific expression patterns and genome architectures to accommodate its survival/thriving in a TWE niche. This research could facilitate flexible clues to the rapidly escalating demands in bioremediation of other toxins and new industrial innovation.

## Author contributions

JL and WZ conceived and designed this study. JL, SQ, LX, CZ, JW, YJ, and HH performed the data collection and analysis. JL, SQ, LX, MS, FZ, RL, and WZ discussed, wrote and finalized the manuscript.

### Conflict of interest statement

The authors declare that the research was conducted in the absence of any commercial or financial relationships that could be construed as a potential conflict of interest.
