# Supplementary material for: Comparative Genomics Reveals Specific Genetic Architectures in Nicotine Metabolism of Pseudomonas sp. JY-Q
Source: Front Microbiol. 2017 Oct 31;8:2085. doi: 10.3389/fmicb.2017.02085 (PMC5674928; doi:10.3389/fmicb.2017.02085)
Supplement: Supplementary file 1 [file DataSheet1.pdf]

**Supplementary Table S1**. *Pseudomonas* sp. JY-Q non-conserved protein-coding genes against strain S16.

| No | Synonym | Start | End | Strand | Length(aa) | Gene | Product |
|---|---|---|---|---|---|---|---|
| 1 | AA098_00065 | 11963 | 13255 | - | 430 | - | beta-lactamase |
| 2 | AA098_00070 | 13416 | 14312 | - | 298 | - | LysR family transcriptional regulator |
| 3 | AA098_00075 | 14541 | 15377 | + | 278 | - | hydrolase |
| 4 | AA098_00080 | 15540 | 17429 | + | 629 | - | hypothetical protein |
| 5 | AA098_00085 | 17446 | 18795 | + | 449 | - | hypothetical protein |
| 6 | AA098_00090 | 18886 | 20016 | + | 376 | - | hypothetical protein |
| 7 | AA098_00095 | 20019 | 22367 | + | 782 | - | RND transporter |
| 8 | AA098_00100 | 22511 | 24217 | + | 568 | - | long-chain fatty acid--CoA ligase |
| 9 | AA098_00105 | 24480 | 25388 | + | 302 | - | LysR family transcriptional regulator |
| 10 | AA098_00110 | 25455 | 27158 | - | 567 | - | choline transporter |
| 11 | AA098_00115 | 27557 | 28747 | + | 396 | - | 2Fe-2S ferredoxin |
| 12 | AA098_00120 | 28758 | 30203 | + | 481 | - | aldehyde dehydrogenase |
| 13 | AA098_00125 | 30216 | 31601 | + | 461 | - | rubredoxin-NAD(+) reductase |
| 14 | AA098_00130 | 31611 | 32939 | + | 442 | - | oxidoreductase |
| 15 | AA098_00135 | 32936 | 33307 | + | 123 | - | ethanolamine utilization protein EutQ |
| 16 | AA098_00140 | 33389 | 34495 | + | 368 | - | MFS transporter |
| 17 | AA098_00145 | 34643 | 34936 | + | 97 | - | GABA permease |
| 18 | AA098_00170 | 40500 | 40880 | - | 126 | - | glutamine synthetase |
| 19 | AA098_00180 | 42200 | 42826 | + | 208 | - | hypothetical protein |
| 20 | AA098_00410 | 92110 | 93138 | + | 342 | - | transcriptional regulator |
| 21 | AA098_00570 | 122757 | 122960 | - | 67 | - | hypothetical protein |
| 22 | AA098_00640 | 136349 | 137191 | + | 280 | - | hypothetical protein |
| 23 | AA098_00755 | 167465 | 167827 | + | 120 | - | AraC family transcriptional regulator |
| 24 | AA098_00785 | 177041 | 179116 | + | 691 | - | Sel1 domain protein repeat-containing protein |
| 25 | AA098_00930 | 206499 | 206933 | + | 144 | - | hypothetical protein |
| 26 | AA098_01330 | 298345 | 299013 | - | 222 | - | NAD(P)H dehydrogenase |
| 27 | AA098_01335 | 299152 | 300006 | + | 284 | - | LysR family transcriptional regulator |

| | | | | | | | |
|---|---|---|---|---|---|---|---|
| 28 | AA098_01360 | 305295 | 305816 | + | 173 | - | transposase |
| 29 | AA098_01365 | 305846 | 306649 | + | 267 | - | integrase |
| 30 | AA098_01570 | 346052 | 346588 | + | 178 | - | fimbrial protein |
| 31 | AA098_01655 | 370813 | 372963 | + | 716 | - | hypothetical protein |
| 32 | AA098_01680 | 379552 | 384237 | + | 1561 | - | hypothetical protein |
| 33 | AA098_01885 | 432817 | 433089 | - | 90 | tatA | preprotein translocase subunit SecA |
| 34 | AA098_01920 | 437348 | 441976 | - | 1542 | - | hypothetical protein |
| 35 | AA098_02050 | 469714 | 470205 | - | 163 | - | hypothetical protein |
| 36 | AA098_02080 | 476864 | 477667 | - | 267 | - | integrase |
| 37 | AA098_02085 | 477697 | 478218 | - | 173 | - | transposase |
| 38 | AA098_02145 | 490106 | 490381 | - | 91 | - | hypothetical protein |
| 39 | AA098_02150 | 490678 | 491166 | - | 162 | - | hypothetical protein |
| 40 | AA098_02155 | 491499 | 491897 | - | 132 | - | transposase |
| 41 | AA098_02260 | 516587 | 517270 | + | 227 | - | hypothetical protein |
| 42 | AA098_02545 | 587108 | 587575 | + | 155 | - | hypothetical protein |
| 43 | AA098_02630 | 603375 | 605006 | + | 543 | - | NAD synthetase |
| 44 | AA098_02645 | 609436 | 609795 | + | 119 | - | hypothetical protein |
| 45 | AA098_02650 | 609867 | 610163 | + | 98 | - | hypothetical protein |
| 46 | AA098_02655 | 610384 | 610677 | - | 97 | - | hypothetical protein |
| 47 | AA098_02660 | 610894 | 611289 | + | 131 | - | hypothetical protein |
| 48 | AA098_02665 | 611293 | 611955 | + | 220 | - | hypothetical protein |
| 49 | AA098_02680 | 614042 | 615100 | - | 352 | - | hypothetical protein |
| 50 | AA098_02685 | 615370 | 615576 | - | 68 | - | hypothetical protein |
| 51 | AA098_02690 | 615882 | 616445 | - | 187 | - | hypothetical protein |
| 52 | AA098_02695 | 618352 | 619656 | - | 434 | - | hypothetical protein |
| 53 | AA098_02700 | 620065 | 622320 | + | 751 | - | hypothetical protein |
| 54 | AA098_02705 | 622479 | 622982 | - | 167 | - | hypothetical protein |
| 55 | AA098_02710 | 622979 | 625090 | - | 703 | - | hypothetical protein |

| | | | | | | | |
|---|---|---|---|---|---|---|---|
| 56 | AA098_02715 | 625090 | 627441 | - | 783 | - | hypothetical protein |
| 57 | AA098_02720 | 627519 | 628781 | - | 420 | - | hypothetical protein |
| 58 | AA098_02765 | 637658 | 637906 | - | 82 | - | hypothetical protein |
| 59 | AA098_02870 | 657874 | 658677 | - | 267 | - | integrase |
| 60 | AA098_02875 | 658707 | 659228 | - | 173 | - | transposase |
| 61 | AA098_02920 | 667625 | 668344 | - | 239 | - | cobalt-precorrin-6X reductase |
| 62 | AA098_02955 | 675368 | 676051 | + | 227 | - | hypothetical protein |
| 63 | AA098_02960 | 676103 | 676792 | + | 229 | - | hypothetical protein |
| 64 | AA098_02965 | 676785 | 677441 | + | 218 | - | hypothetical protein |
| 65 | AA098_03165 | 717273 | 717599 | + | 108 | - | hypothetical protein |
| 66 | AA098_03235 | 731257 | 731628 | - | 123 | - | hypothetical protein |
| 67 | AA098_03240 | 731907 | 732344 | - | 145 | - | transposase |
| 68 | AA098_03245 | 732392 | 732760 | - | 122 | - | transposase |
| 69 | AA098_03250 | 732841 | 733110 | - | 89 | - | hypothetical protein |
| 70 | AA098_03255 | 733345 | 735051 | - | 568 | - | diguanylate cyclase |
| 71 | AA098_03265 | 736299 | 737327 | - | 342 | - | transcriptional regulator |
| 72 | AA098_03270 | 737365 | 737769 | - | 134 | - | hypothetical protein |
| 73 | AA098_03280 | 739177 | 739545 | + | 122 | - | transposase |
| 74 | AA098_03285 | 739593 | 740030 | + | 145 | - | transposase |
| 75 | AA098_03290 | 740069 | 741733 | + | 554 | - | hypothetical protein |
| 76 | AA098_03295 | 741903 | 743774 | + | 623 | - | ATPase |
| 77 | AA098_03300 | 743755 | 744690 | + | 311 | - | hypothetical protein |
| 78 | AA098_03305 | 744687 | 745955 | + | 422 | - | hypothetical protein |
| 79 | AA098_03310 | 746534 | 746788 | + | 84 | - | hypothetical protein |
| 80 | AA098_03315 | 747099 | 747386 | + | 95 | - | hypothetical protein |
| 81 | AA098_03320 | 747438 | 747860 | + | 140 | - | hypothetical protein |
| 82 | AA098_03325 | 747959 | 750493 | + | 844 | - | ATP-dependent helicase |
| 83 | AA098_03330 | 750650 | 751114 | - | 154 | - | Fis family transcriptional regulator |

| | | | | | | | |
|---|---|---|---|---|---|---|---|
| 84 | AA098_03335 | 751397 | 751738 | + | 113 | - | hypothetical protein |
| 85 | AA098_03340 | 751894 | 752457 | + | 187 | - | hypothetical protein |
| 86 | AA098_03345 | 752537 | 753265 | + | 242 | - | serine/threonine protein phosphatase |
| 87 | AA098_03350 | 753323 | 753682 | + | 119 | - | hypothetical protein |
| 88 | AA098_03355 | 754130 | 754471 | + | 113 | - | hypothetical protein |
| 89 | AA098_03360 | 755153 | 755770 | + | 205 | - | XRE family transcriptional regulator |
| 90 | AA098_03365 | 755767 | 756588 | + | 273 | - | hypothetical protein |
| 91 | AA098_03370 | 756662 | 757018 | + | 118 | - | hypothetical protein |
| 92 | AA098_03375 | 757496 | 759322 | + | 608 | - | hypothetical protein |
| 93 | AA098_03380 | 759724 | 760107 | + | 127 | - | hypothetical protein |
| 94 | AA098_03385 | 760251 | 761159 | + | 302 | - | hypothetical protein |
| 95 | AA098_03390 | 762565 | 762933 | + | 122 | - | transcriptional regulator |
| 96 | AA098_03395 | 763127 | 763357 | + | 76 | - | hypothetical protein |
| 97 | AA098_03400 | 763420 | 763629 | + | 69 | - | hypothetical protein |
| 98 | AA098_03420 | 766141 | 767628 | - | 495 | - | hypothetical protein |
| 99 | AA098_03430 | 768039 | 768488 | - | 149 | - | hypothetical protein |
| 100 | AA098_03435 | 768504 | 768839 | + | 111 | - | hypothetical protein |
| 101 | AA098_03440 | 768850 | 769170 | + | 106 | - | hypothetical protein |
| 102 | AA098_03445 | 769816 | 770286 | - | 156 | - | hypothetical protein |
| 103 | AA098_03450 | 770570 | 771946 | + | 458 | - | hypothetical protein |
| 104 | AA098_03455 | 772039 | 772398 | + | 119 | - | transposase |
| 105 | AA098_03475 | 775677 | 777143 | - | 488 | - | integrase |
| 106 | AA098_03480 | 777121 | 777747 | - | 208 | - | hypothetical protein |
| 107 | AA098_03485 | 777744 | 779846 | - | 700 | - | integrase |
| 108 | AA098_03490 | 779830 | 782190 | - | 786 | - | hypothetical protein |
| 109 | AA098_03500 | 784488 | 785006 | - | 172 | - | hypothetical protein |
| 110 | AA098_03555 | 795182 | 796180 | - | 332 | - | fimbrial protein |
| 111 | AA098_03560 | 796207 | 798585 | - | 792 | - | usher CupC3 |

| | | | | | | | |
|---|---|---|---|---|---|---|---|
| 112 | AA098_03565 | 798831 | 799553 | - | 240 | - | molecular chaperone |
| 113 | AA098_03570 | 799611 | 800195 | - | 194 | - | fimbrial protein |
| 114 | AA098_03575 | 800858 | 801505 | - | 215 | - | LuxR family transcriptional regulator |
| 115 | AA098_03580 | 801570 | 804791 | - | 1073 | - | chemotaxis protein CheY |
| 116 | AA098_03585 | 804871 | 806067 | - | 398 | - | diguanylate phosphodiesterase |
| 117 | AA098_03590 | 806054 | 808885 | - | 943 | - | histidine kinase |
| 118 | AA098_03645 | 819826 | 820029 | + | 67 | - | lipoprotein |
| 119 | AA098_03650 | 820046 | 820498 | + | 150 | - | hypothetical protein |
| 120 | AA098_03655 | 820977 | 821729 | + | 250 | - | hypothetical protein |
| 121 | AA098_03660 | 822217 | 824610 | + | 797 | - | ligand-gated channel |
| 122 | AA098_03665 | 824772 | 825509 | + | 245 | - | hypothetical protein |
| 123 | AA098_03670 | 825611 | 826486 | - | 291 | - | transcriptional regulator |
| 124 | AA098_03675 | 826606 | 826866 | + | 86 | - | hypothetical protein |
| 125 | AA098_03680 | 827171 | 827851 | + | 226 | - | hypothetical protein |
| 126 | AA098_03685 | 828933 | 831755 | + | 940 | - | hypothetical protein |
| 127 | AA098_03690 | 831755 | 834217 | + | 820 | - | hypothetical protein |
| 128 | AA098_03695 | 834220 | 835104 | + | 294 | - | hypothetical protein |
| 129 | AA098_03705 | 838168 | 838926 | + | 252 | - | hypothetical protein |
| 130 | AA098_03710 | 838923 | 843359 | + | 1478 | - | hypothetical protein |
| 131 | AA098_03715 | 843369 | 843896 | + | 175 | - | PLD-like domain protein |
| 132 | AA098_03720 | 843893 | 846076 | + | 727 | - | hypothetical protein |
| 133 | AA098_03725 | 846212 | 846601 | - | 129 | - | 6-pyruvoyl tetrahydrobiopterin synthase |
| 134 | AA098_03730 | 846652 | 847284 | - | 210 | - | 7-cyano-7-deazaguanine reductase |
| 135 | AA098_03735 | 847281 | 847985 | - | 234 | - | 7-cyano-7-deazaguanine synthase |
| 136 | AA098_03740 | 848014 | 849069 | - | 351 | - | hypothetical protein |
| 137 | AA098_03745 | 849066 | 850364 | - | 432 | - | hypothetical protein |
| 138 | AA098_03750 | 850361 | 851482 | - | 373 | - | hypothetical protein |
| 139 | AA098_03755 | 851479 | 852414 | - | 311 | - | hypothetical protein |

| | | | | | | | |
|---|---|---|---|---|---|---|---|
| 140 | AA098_03760 | 852587 | 853651 | + | 354 | - | hypothetical protein |
| 141 | AA098_03765 | 853773 | 854120 | + | 115 | - | hypothetical protein |
| 142 | AA098_03780 | 854887 | 855129 | - | 80 | - | hypothetical protein |
| 143 | AA098_03790 | 856578 | 856877 | + | 99 | - | hypothetical protein |
| 144 | AA098_03795 | 856870 | 857478 | + | 202 | - | amino acid transporter LysE |
| 145 | AA098_03800 | 857507 | 857785 | + | 92 | - | TnpA protein |
| 146 | AA098_03805 | 858285 | 858740 | + | 151 | - | transposase |
| 147 | AA098_03815 | 859743 | 860774 | + | 343 | - | cytochrome oxidase I |
| 148 | AA098_03820 | 861246 | 861473 | - | 75 | - | preprotein translocase subunit TatA |
| 149 | AA098_03830 | 863401 | 863856 | + | 151 | - | transposase |
| 150 | AA098_03845 | 864781 | 866298 | + | 505 | - | transposase |
| 151 | AA098_03860 | 868087 | 869592 | - | 501 | - | DNA polymerase |
| 152 | AA098_03870 | 871101 | 873470 | + | 789 | - | carbon monoxide dehydrogenase |
| 153 | AA098_03875 | 873457 | 874287 | + | 276 | - | hypothetical protein |
| 154 | AA098_03880 | 875571 | 876908 | + | 445 | - | major facilitator transporter |
| 155 | AA098_03885 | 877115 | 878296 | + | 393 | - | para-nitrophenol 4-monooxygenase |
| 156 | AA098_03890 | 879618 | 880367 | + | 249 | - | Asp/Glu racemase |
| 157 | AA098_03895 | 880367 | 881149 | + | 260 | - | alpha/beta hydrolase |
| 158 | AA098_03900 | 881151 | 882182 | + | 343 | - | leucyl aminopeptidase |
| 159 | AA098_03905 | 883199 | 884524 | + | 441 | - | hypothetical protein |
| 160 | AA098_03910 | 884902 | 886791 | + | 629 | - | chemotaxis protein |
| 161 | AA098_03915 | 887278 | 888306 | + | 342 | - | transcriptional regulator |
| 162 | AA098_03920 | 888459 | 889034 | - | 191 | - | recombinase |
| 163 | AA098_03925 | 889261 | 890235 | + | 324 | - | integrase |
| 164 | AA098_03930 | 890784 | 891149 | + | 121 | - | hypothetical protein |
| 165 | AA098_03935 | 891519 | 891980 | - | 153 | - | hypothetical protein |
| 166 | AA098_03940 | 892391 | 893059 | - | 222 | - | hypothetical protein |
| 167 | AA098_03945 | 893133 | 893570 | - | 145 | - | transposase |

| | | | | | | | |
|---|---|---|---|---|---|---|---|
| 168 | AA098_03950 | 893618 | 893986 | - | 122 | - | transposase |
| 169 | AA098_03955 | 894067 | 894336 | - | 89 | - | hypothetical protein |
| 170 | AA098_03960 | 894571 | 896277 | - | 568 | - | diguanylate cyclase |
| 171 | AA098_03965 | 896314 | 897879 | - | 521 | - | chemotaxis protein |
| 172 | AA098_03970 | 898115 | 898483 | + | 122 | - | transposase |
| 173 | AA098_03975 | 898531 | 898968 | + | 145 | - | transposase |
| 174 | AA098_03995 | 903374 | 904879 | + | 501 | - | DNA polymerase |
| 175 | AA098_04005 | 905614 | 906510 | + | 298 | - | LysR family transcriptional regulator |
| 176 | AA098_04015 | 909172 | 909774 | + | 200 | - | molybdopterin-guanine dinucleotide biosynthesis protein MobA |
| 177 | AA098_04020 | 909781 | 910812 | + | 343 | - | cytochrome oxidase I |
| 178 | AA098_04025 | 911284 | 911511 | - | 75 | - | preprotein translocase subunit TatA |
| 179 | AA098_04030 | 911863 | 912891 | + | 342 | - | transcriptional regulator |
| 180 | AA098_04040 | 913974 | 914816 | - | 280 | - | transposase |
| 181 | AA098_04045 | 914873 | 915196 | - | 107 | - | transposase |
| 182 | AA098_04055 | 915961 | 916284 | - | 107 | - | transposase |
| 183 | AA098_04060 | 916411 | 917091 | + | 226 | - | hypothetical protein |
| 184 | AA098_04070 | 918896 | 919186 | - | 96 | - | hypothetical protein |
| 185 | AA098_04075 | 919206 | 919565 | - | 119 | - | transposase |
| 186 | AA098_04080 | 919603 | 919815 | + | 70 | - | hypothetical protein |
| 187 | AA098_04085 | 920929 | 921315 | - | 128 | - | hypothetical protein |
| 188 | AA098_04090 | 921541 | 922974 | - | 477 | - | aldehyde dehydrogenase |
| 189 | AA098_04100 | 924939 | 926375 | - | 478 | - | hypothetical protein |
| 190 | AA098_04105 | 926434 | 926772 | - | 112 | - | hypothetical protein |
| 191 | AA098_04110 | 927078 | 928082 | - | 334 | - | hypothetical protein |
| 192 | AA098_04115 | 928356 | 929384 | - | 342 | - | transcriptional regulator |
| 193 | AA098_04120 | 929448 | 930281 | + | 277 | - | hypothetical protein |
| 194 | AA098_04125 | 930426 | 931481 | - | 351 | - | ATPase AAA |
| 195 | AA098_04130 | 931481 | 931696 | - | 71 | - | AlpA family transcriptional regulator |

| | | | | | | | |
|---|---|---|---|---|---|---|---|
| 196 | AA098_04135 | 932177 | 937078 | - | 1633 | - | damage-inducible protein |
| 197 | AA098_04140 | 937219 | 938424 | - | 401 | - | integrase |
| 198 | AA098_04190 | 949204 | 950007 | - | 267 | - | integrase |
| 199 | AA098_04195 | 950037 | 950558 | - | 173 | - | transposase |
| 200 | AA098_04305 | 974601 | 974999 | + | 132 | - | ribosome-binding factor A |
| 201 | AA098_04490 | 1016935 | 1017546 | + | 203 | - | sulfite oxidase |
| 202 | AA098_04585 | 1042319 | 1043083 | + | 254 | - | membrane protein |
| 203 | AA098_04655 | 1058307 | 1058462 | + | 51 | - | cyanide insensitive terminal oxidase, subunit III |
| 204 | AA098_04665 | 1059789 | 1060496 | - | 235 | - | LuxR family transcriptional regulator |
| 205 | AA098_04715 | 1071402 | 1072226 | + | 274 | - | 5-methyltetrahydropteroyltriglutamate--homocysteine methyltransferase |
| 206 | AA098_04755 | 1078645 | 1079580 | + | 311 | - | MerR family transcriptional regulator |
| 207 | AA098_04765 | 1080242 | 1080670 | - | 142 | - | histidine kinase |
| 208 | AA098_04795 | 1084779 | 1088003 | + | 1074 | - | hypothetical protein |
| 209 | AA098_04965 | 1120841 | 1121293 | + | 150 | - | hypothetical protein |
| 210 | AA098_05390 | 1214684 | 1215043 | + | 119 | - | pilus assembly protein PilZ |
| 211 | AA098_05525 | 1248806 | 1249420 | + | 204 | - | 3-beta hydroxysteroid dehydrogenase |
| 212 | AA098_05625 | 1270781 | 1270993 | - | 70 | - | helicase |
| 213 | AA098_05630 | 1270986 | 1272467 | - | 493 | - | diguanylate cyclase |
| 214 | AA098_05635 | 1272680 | 1273129 | + | 149 | - | lipoprotein |
| 215 | AA098_05650 | 1275125 | 1276807 | - | 560 | - | 5'-nucleotidase |
| 216 | AA098_05795 | 1307390 | 1308340 | + | 316 | - | transposase |
| 217 | AA098_05855 | 1324633 | 1324836 | + | 67 | - | Fe-S protein |
| 218 | AA098_05915 | 1334767 | 1336572 | - | 601 | - | sodium:proton antiporter |
| 219 | AA098_05940 | 1341422 | 1341841 | + | 139 | - | histidine kinase |
| 220 | AA098_05945 | 1341921 | 1342202 | + | 93 | - | hypothetical protein |
| 221 | AA098_05970 | 1346969 | 1347511 | - | 180 | - | hypothetical protein |
| 222 | AA098_05975 | 1347549 | 1348472 | - | 307 | - | hypothetical protein |
| 223 | AA098_05980 | 1348469 | 1348795 | - | 108 | - | hypothetical protein |

| | | | | | | | |
|---|---|---|---|---|---|---|---|
| 224 | AA098_05990 | 1349796 | 1350074 | - | 92 | - | hypothetical protein |
| 225 | AA098_06445 | 1452188 | 1453255 | + | 355 | - | hypothetical protein |
| 226 | AA098_06470 | 1457003 | 1457212 | + | 69 | - | hypothetical protein |
| 227 | AA098_06600 | 1480541 | 1481881 | + | 446 | - | cytochrome C |
| 228 | AA098_06605 | 1481886 | 1482377 | + | 163 | - | sorbitol dehydrogenase |
| 229 | AA098_06610 | 1482374 | 1484632 | + | 752 | - | dehydrogenase |
| 230 | AA098_06730 | 1505656 | 1505916 | - | 86 | - | hypothetical protein |
| 231 | AA098_06755 | 1510338 | 1510757 | + | 139 | - | Sel1 domain-containing protein repeat-containing protein |
| 232 | AA098_06775 | 1518076 | 1518807 | + | 243 | - | GntR family transcriptional regulator |
| 233 | AA098_06780 | 1518902 | 1520176 | + | 424 | - | MFS transporter |
| 234 | AA098_06785 | 1520201 | 1521034 | + | 277 | - | membrane protein |
| 235 | AA098_06795 | 1523074 | 1523391 | - | 105 | - | molecular chaperone |
| 236 | AA098_06830 | 1526412 | 1527215 | - | 267 | - | integrase |
| 237 | AA098_06835 | 1527245 | 1527766 | - | 173 | - | transposase |
| 238 | AA098_06850 | 1534946 | 1535308 | + | 120 | - | nitrite reductase |
| 239 | AA098_06865 | 1537348 | 1538616 | - | 422 | - | hypothetical protein |
| 240 | AA098_06995 | 1564816 | 1565181 | + | 121 | - | hypothetical protein |
| 241 | AA098_07020 | 1570697 | 1571176 | + | 159 | - | adhesin |
| 242 | AA098_07025 | 1571267 | 1573777 | + | 836 | - | pilus assembly protein PapC |
| 243 | AA098_07030 | 1573894 | 1574628 | + | 244 | - | pilus assembly protein |
| 244 | AA098_07035 | 1574625 | 1575821 | + | 398 | - | hypothetical protein |
| 245 | AA098_07040 | 1575851 | 1576477 | + | 208 | - | LuxR family transcriptional regulator |
| 246 | AA098_07045 | 1576487 | 1579729 | + | 1080 | - | histidine kinase |
| 247 | AA098_07055 | 1580827 | 1581312 | + | 161 | - | hypothetical protein |
| 248 | AA098_07060 | 1581382 | 1581876 | + | 164 | - | hypothetical protein |
| 249 | AA098_07065 | 1581952 | 1584420 | + | 822 | - | pilus assembly protein PapC |
| 250 | AA098_07070 | 1584472 | 1585245 | + | 257 | - | pilus assembly protein |
| 251 | AA098_07075 | 1585257 | 1586420 | + | 387 | - | hypothetical protein |

| | | | | | | | |
|---|---|---|---|---|---|---|---|
| 252 | AA098_07080 | 1586494 | 1586646 | - | 50 | - | membrane protein |
| 253 | AA098_07085 | 1586938 | 1587117 | + | 59 | - | carbon storage regulator |
| 254 | AA098_07110 | 1592408 | 1593598 | - | 396 | - | integrase |
| 255 | AA098_07120 | 1594310 | 1595044 | - | 244 | - | serine/threonine protein phosphatase |
| 256 | AA098_07125 | 1595041 | 1595361 | - | 106 | - | hypothetical protein |
| 257 | AA098_07130 | 1595358 | 1596092 | - | 244 | - | hypothetical protein |
| 258 | AA098_07135 | 1596229 | 1596588 | - | 119 | - | hypothetical protein |
| 259 | AA098_07140 | 1596776 | 1597003 | - | 75 | - | hypothetical protein |
| 260 | AA098_07145 | 1596993 | 1597184 | - | 63 | - | carbon storage regulator CsrA |
| 261 | AA098_07150 | 1597234 | 1598067 | - | 277 | - | transposase |
| 262 | AA098_07155 | 1598064 | 1598312 | - | 82 | - | hypothetical protein |
| 263 | AA098_07160 | 1598386 | 1599171 | - | 261 | - | prophage antirepressor |
| 264 | AA098_07165 | 1599279 | 1599551 | - | 90 | - | hypothetical protein |
| 265 | AA098_07170 | 1599589 | 1599981 | - | 130 | - | LuxR family transcriptional regulator |
| 266 | AA098_07180 | 1601440 | 1601715 | + | 91 | - | hypothetical protein |
| 267 | AA098_07185 | 1601712 | 1602017 | + | 101 | - | hypothetical protein |
| 268 | AA098_07190 | 1602014 | 1602250 | + | 78 | - | hypothetical protein |
| 269 | AA098_07195 | 1602247 | 1602546 | + | 99 | - | hypothetical protein |
| 270 | AA098_07200 | 1602534 | 1603280 | + | 248 | - | hypothetical protein |
| 271 | AA098_07205 | 1603277 | 1603588 | + | 103 | - | hypothetical protein |
| 272 | AA098_07210 | 1603585 | 1604148 | + | 187 | - | hypothetical protein |
| 273 | AA098_07215 | 1604145 | 1604375 | + | 76 | - | phage-like protein |
| 274 | AA098_07220 | 1604372 | 1605139 | + | 255 | - | hypothetical protein |
| 275 | AA098_07225 | 1605126 | 1605944 | + | 272 | - | DNA replication protein DnaC |
| 276 | AA098_07230 | 1605941 | 1607353 | + | 470 | - | helicase DnaB |
| 277 | AA098_07235 | 1607340 | 1607852 | + | 170 | - | hypothetical protein |
| 278 | AA098_07240 | 1607849 | 1608148 | + | 99 | - | hypothetical protein |
| 279 | AA098_07250 | 1609096 | 1609344 | + | 82 | - | hypothetical protein |

| | | | | | | | |
|---|---|---|---|---|---|---|---|
| 280 | AA098_07255 | 1609742 | 1609930 | - | 62 | - | hypothetical protein |
| 281 | AA098_07260 | 1610347 | 1610580 | - | 77 | - | hypothetical protein |
| 282 | AA098_07265 | 1610866 | 1611198 | + | 110 | - | holin |
| 283 | AA098_07270 | 1611265 | 1611495 | - | 76 | - | hypothetical protein |
| 284 | AA098_07275 | 1612075 | 1612380 | + | 101 | - | HNH endonuclease |
| 285 | AA098_07280 | 1612526 | 1613023 | + | 165 | - | terminase |
| 286 | AA098_07285 | 1613026 | 1614693 | + | 555 | - | terminase |
| 287 | AA098_07290 | 1614751 | 1616700 | + | 649 | - | phage capsid protein |
| 288 | AA098_07295 | 1616754 | 1616972 | + | 72 | - | hypothetical protein |
| 289 | AA098_07300 | 1616987 | 1618354 | + | 455 | - | poly(3-hydroxybutyrate) depolymerase |
| 290 | AA098_07305 | 1618341 | 1619267 | + | 308 | - | phage portal protein |
| 291 | AA098_07310 | 1619264 | 1619587 | + | 107 | - | hypothetical protein |
| 292 | AA098_07315 | 1619590 | 1619928 | + | 112 | - | head-tail adaptor protein |
| 293 | AA098_07320 | 1619921 | 1620406 | + | 161 | - | hypothetical protein |
| 294 | AA098_07325 | 1620403 | 1620771 | + | 122 | - | hypothetical protein |
| 295 | AA098_07330 | 1620834 | 1621325 | + | 163 | - | phage major tail protein |
| 296 | AA098_07335 | 1621329 | 1621805 | + | 158 | - | phage tail assembly protein |
| 297 | AA098_07340 | 1622206 | 1623234 | - | 342 | - | transcriptional regulator |
| 298 | AA098_07345 | 1623226 | 1623999 | + | 257 | - | hypothetical protein |
| 299 | AA098_07350 | 1624038 | 1626494 | + | 818 | - | tail tape measure protein |
| 300 | AA098_07355 | 1626491 | 1626973 | + | 160 | - | hypothetical protein |
| 301 | AA098_07360 | 1627033 | 1627278 | - | 81 | - | hypothetical protein |
| 302 | AA098_07365 | 1627357 | 1627776 | + | 139 | - | hypothetical protein |
| 303 | AA098_07370 | 1627748 | 1630846 | + | 1032 | - | hypothetical protein |
| 304 | AA098_07375 | 1630861 | 1631319 | + | 152 | - | membrane protein |
| 305 | AA098_07380 | 1631431 | 1633680 | + | 749 | - | hypothetical protein |
| 306 | AA098_07385 | 1633731 | 1634759 | - | 342 | - | transcriptional regulator |
| 307 | AA098_07390 | 1635084 | 1635572 | + | 162 | - | glycoside hydrolase |

| | | | | | | | |
|---|---|---|---|---|---|---|---|
| 308 | AA098_07395 | 1635569 | 1636090 | + | 173 | - | hypothetical protein |
| 309 | AA098_07400 | 1636125 | 1636541 | - | 138 | - | hypothetical protein |
| 310 | AA098_07525 | 1668864 | 1669907 | + | 347 | - | hypothetical protein |
| 311 | AA098_07530 | 1669956 | 1671206 | + | 416 | - | hypothetical protein |
| 312 | AA098_07535 | 1671255 | 1672256 | + | 333 | - | hemolytic protein HlpA |
| 313 | AA098_07540 | 1672260 | 1673285 | + | 341 | - | hypothetical protein |
| 314 | AA098_07545 | 1673352 | 1674563 | + | 403 | - | hypothetical protein |
| 315 | AA098_07550 | 1674556 | 1675590 | + | 344 | - | UDP-glucose 4-epimerase |
| 316 | AA098_07555 | 1675595 | 1676713 | + | 372 | - | capsular biosynthesis protein |
| 317 | AA098_07560 | 1676736 | 1677866 | + | 376 | - | UDP-N-acetylglucosamine 2-epimerase |
| 318 | AA098_07565 | 1677881 | 1679131 | + | 416 | - | glycosyl transferase |
| 319 | AA098_07570 | 1679131 | 1680096 | + | 321 | - | NAD-dependent dehydratase |
| 320 | AA098_07575 | 1680093 | 1681103 | + | 336 | - | glycosyl transferase |
| 321 | AA098_07580 | 1681169 | 1683166 | + | 665 | - | membrane protein |
| 322 | AA098_07585 | 1683311 | 1684381 | + | 356 | - | spore coat protein |
| 323 | AA098_07590 | 1684378 | 1685283 | + | 301 | - | dTDP-4-dehydrorhamnose reductase |
| 324 | AA098_07595 | 1685280 | 1686161 | + | 293 | - | glucose-1-phosphate thymidylyltransferase |
| 325 | AA098_07600 | 1686998 | 1694536 | + | 2512 | - | beta-ketoacyl synthase |
| 326 | AA098_07605 | 1694546 | 1695898 | + | 450 | - | 8-amino-7-oxononanoate synthase |
| 327 | AA098_07610 | 1695908 | 1696363 | - | 151 | - | transposase |
| 328 | AA098_07615 | 1696722 | 1697765 | - | 347 | - | phytanoyl-CoA dioxygenase |
| 329 | AA098_07620 | 1697769 | 1699913 | - | 714 | - | capsular biosynthesis protein |
| 330 | AA098_07625 | 1699913 | 1700479 | - | 188 | - | GCN5 family acetyltransferase |
| 331 | AA098_07630 | 1700484 | 1702031 | - | 515 | - | sulfatase |
| 332 | AA098_07635 | 1702039 | 1702815 | - | 258 | - | short-chain dehydrogenase |
| 333 | AA098_07640 | 1702812 | 1704131 | - | 439 | - | capsule biosynthesis protein CapA |
| 334 | AA098_07645 | 1704071 | 1706092 | - | 673 | - | capsular biosynthesis protein |
| 335 | AA098_07650 | 1706394 | 1707266 | - | 290 | - | phosphotransferase |

| | | | | | | | |
|---|---|---|---|---|---|---|---|
| 336 | AA098_07660 | 1710279 | 1711211 | + | 310 | - | arabinose 5-phosphate isomerase |
| 337 | AA098_07665 | 1711368 | 1712669 | + | 433 | - | hypothetical protein |
| 338 | AA098_07670 | 1712725 | 1713288 | - | 187 | - | 3-deoxy-D-manno-octulosonate 8-phosphate phosphatase |
| 339 | AA098_07675 | 1714030 | 1714872 | - | 280 | - | 2-dehydro-3-deoxyphosphooctonate aldolase |
| 340 | AA098_07685 | 1718557 | 1719648 | - | 363 | - | sugar transporter |
| 341 | AA098_07690 | 1719641 | 1720747 | - | 368 | - | ABC transporter permease |
| 342 | AA098_07695 | 1720801 | 1721454 | - | 217 | - | ABC transporter ATP-binding protein |
| 343 | AA098_07705 | 1722703 | 1723350 | - | 215 | - | phosphate ABC transporter permease |
| 344 | AA098_07710 | 1724003 | 1724806 | + | 267 | - | hypothetical protein |
| 345 | AA098_07715 | 1725005 | 1725280 | - | 91 | - | hypothetical protein |
| 346 | AA098_07740 | 1730013 | 1730756 | - | 247 | - | hypothetical protein |
| 347 | AA098_07830 | 1746407 | 1747357 | + | 316 | - | transposase |
| 348 | AA098_07905 | 1761741 | 1762625 | + | 294 | - | LysR family transcriptional regulator |
| 349 | AA098_07910 | 1762665 | 1763414 | - | 249 | - | oxidoreductase |
| 350 | AA098_08035 | 1787128 | 1788120 | - | 330 | - | hypothetical protein |
| 351 | AA098_08070 | 1790419 | 1791483 | - | 354 | - | hypothetical protein |
| 352 | AA098_08075 | 1791526 | 1794033 | - | 835 | - | ferrous iron transporter B |
| 353 | AA098_08080 | 1794269 | 1799482 | - | 1737 | - | hypothetical protein |
| 354 | AA098_08085 | 1799506 | 1800783 | - | 425 | - | hypothetical protein |
| 355 | AA098_08090 | 1801040 | 1802884 | + | 614 | - | hypothetical protein |
| 356 | AA098_08095 | 1802944 | 1803840 | + | 298 | - | hypothetical protein |
| 357 | AA098_08105 | 1804938 | 1806110 | + | 390 | - | hypothetical protein |
| 358 | AA098_08110 | 1806099 | 1807127 | - | 342 | - | transcriptional regulator |
| 359 | AA098_08115 | 1807152 | 1807355 | - | 67 | - | hypothetical protein |
| 360 | AA098_08120 | 1807392 | 1807598 | + | 68 | - | hypothetical protein |
| 361 | AA098_08125 | 1807595 | 1809880 | + | 761 | - | hypothetical protein |
| 362 | AA098_08130 | 1810000 | 1811028 | + | 342 | - | transcriptional regulator |
| 363 | AA098_08135 | 1811031 | 1811552 | + | 173 | - | hypothetical protein |

| | | | | | | | |
|---|---|---|---|---|---|---|---|
| 364 | AA098_08140 | 1811629 | 1815618 | + | 1329 | - | hypothetical protein |
| 365 | AA098_08145 | 1815823 | 1817847 | + | 674 | - | hypothetical protein |
| 366 | AA098_08150 | 1818034 | 1820175 | + | 713 | - | membrane protein |
| 367 | AA098_08155 | 1820235 | 1821731 | + | 498 | - | hypothetical protein |
| 368 | AA098_08160 | 1821737 | 1822372 | - | 211 | - | pilus assembly protein |
| 369 | AA098_08165 | 1822537 | 1823034 | - | 165 | - | fimbrial protein |
| 370 | AA098_08220 | 1834241 | 1834426 | + | 61 | - | hypothetical protein |
| 371 | AA098_08345 | 1859517 | 1859918 | + | 133 | - | cupin |
| 372 | AA098_08470 | 1883672 | 1884850 | + | 392 | - | integrase |
| 373 | AA098_08565 | 1903002 | 1904645 | - | 547 | - | hypothetical protein |
| 374 | AA098_08570 | 1904671 | 1905945 | - | 424 | - | hypothetical protein |
| 375 | AA098_08695 | 1936749 | 1938059 | + | 436 | - | type IV secretion protein Rhs |
| 376 | AA098_08720 | 1942964 | 1945108 | - | 714 | - | HAD family hydrolase |
| 377 | AA098_08725 | 1945505 | 1945768 | + | 87 | - | hypothetical protein |
| 378 | AA098_08755 | 1952076 | 1952324 | - | 82 | - | hypothetical protein |
| 379 | AA098_08765 | 1953010 | 1954107 | + | 365 | - | RND transporter MFP subunit |
| 380 | AA098_08770 | 1954104 | 1957187 | + | 1027 | - | acriflavine resistance protein B |
| 381 | AA098_08775 | 1957241 | 1957978 | - | 245 | - | GntR family transcriptional regulator |
| 382 | AA098_08780 | 1958072 | 1959586 | - | 504 | - | DSBA oxidoreductase |
| 383 | AA098_08785 | 1959576 | 1960721 | - | 381 | - | hemolysin D |
| 384 | AA098_08790 | 1960928 | 1962208 | - | 426 | - | multidrug transporter |
| 385 | AA098_08795 | 1962450 | 1963160 | - | 236 | - | AraC family transcriptional regulator |
| 386 | AA098_08800 | 1963330 | 1963716 | - | 128 | - | cupin |
| 387 | AA098_08805 | 1963825 | 1964649 | - | 274 | - | AraC family transcriptional regulator |
| 388 | AA098_08810 | 1964669 | 1965112 | - | 147 | - | GNAT family acetyltransferase |
| 389 | AA098_08825 | 1967514 | 1968983 | - | 489 | - | hypothetical protein |
| 390 | AA098_08835 | 1970141 | 1972801 | - | 886 | - | hypothetical protein |
| 391 | AA098_08975 | 2011590 | 2011964 | - | 124 | - | membrane protein |

| | | | | | | | |
|---|---|---|---|---|---|---|---|
| 392 | AA098_08995 | 2014231 | 2014662 | + | 143 | - | peptidase M15 |
| 393 | AA098_09025 | 2023119 | 2023982 | - | 287 | - | transposase |
| 394 | AA098_09030 | 2023979 | 2024272 | - | 97 | - | transposase |
| 395 | AA098_09245 | 2075117 | 2075644 | + | 175 | - | copper-binding protein |
| 396 | AA098_09300 | 2085460 | 2085768 | + | 102 | - | chorismate mutase |
| 397 | AA098_09310 | 2086519 | 2086710 | + | 63 | - | hypothetical protein |
| 398 | AA098_09315 | 2087337 | 2087900 | + | 187 | - | TetR family transcriptional regulator |
| 399 | AA098_09320 | 2087897 | 2088874 | + | 325 | - | hypothetical protein |
| 400 | AA098_09325 | 2088950 | 2089816 | - | 288 | - | oxaloacetate decarboxylase |
| 401 | AA098_09330 | 2089949 | 2090557 | - | 202 | - | fumarate hydrolyase |
| 402 | AA098_09345 | 2093006 | 2093932 | + | 308 | - | LysR family transcriptional regulator |
| 403 | AA098_09350 | 2094309 | 2094794 | + | 161 | - | conserved secreted protein with internal repeat protein |
| 404 | AA098_09360 | 2095693 | 2096586 | - | 297 | - | LysR family transcriptional regulator |
| 405 | AA098_09390 | 2101875 | 2102396 | + | 173 | - | ProQ activator of osmoprotectant transporter prop |
| 406 | AA098_09500 | 2124061 | 2124360 | - | 99 | - | copper resistance protein CopB |
| 407 | AA098_09535 | 2131899 | 2136401 | + | 1500 | - | hypothetical protein |
| 408 | AA098_09540 | 2136486 | 2140931 | + | 1481 | - | leucine-rich repeat-containing protein |
| 409 | AA098_09570 | 2147232 | 2147435 | + | 67 | - | hypothetical protein |
| 410 | AA098_09580 | 2148216 | 2148410 | + | 64 | - | hypothetical protein |
| 411 | AA098_09605 | 2150085 | 2150573 | - | 162 | - | monovalent cation/H+ antiporter subunit E |
| 412 | AA098_09640 | 2157934 | 2158926 | + | 330 | - | AraC family transcriptional regulator |
| 413 | AA098_09645 | 2159008 | 2159388 | + | 126 | - | antibiotic biosynthesis monooxygenase |
| 414 | AA098_09705 | 2172477 | 2172572 | - | 31 | - | membrane protein |
| 415 | AA098_09720 | 2176696 | 2177235 | + | 179 | - | phosphoglycerate mutase |
| 416 | AA098_09785 | 2189265 | 2189579 | - | 104 | - | Cro/CI family transcriptional regulator |
| 417 | AA098_09910 | 2214524 | 2215321 | + | 265 | - | acyl-CoA thioesterase |
| 418 | AA098_10075 | 2250444 | 2250656 | - | 70 | - | DNA-binding protein |
| 419 | AA098_10080 | 2250881 | 2251198 | - | 105 | - | hypothetical protein |

| | | | | | | | |
|---|---|---|---|---|---|---|---|
| 420 | AA098_10085 | 2251252 | 2251734 | - | 160 | - | methyltransferase |
| 421 | AA098_10090 | 2251731 | 2252126 | - | 131 | - | hypothetical protein |
| 422 | AA098_10100 | 2252664 | 2253038 | - | 124 | - | hypothetical protein |
| 423 | AA098_10105 | 2253123 | 2253311 | - | 62 | - | hypothetical protein |
| 424 | AA098_10115 | 2254186 | 2254911 | - | 241 | - | hypothetical protein |
| 425 | AA098_10120 | 2254908 | 2255153 | - | 81 | - | hypothetical protein |
| 426 | AA098_10125 | 2255217 | 2255816 | - | 199 | - | hypothetical protein |
| 427 | AA098_10135 | 2256985 | 2257533 | - | 182 | - | single-stranded DNA-binding protein |
| 428 | AA098_10140 | 2257542 | 2258213 | - | 223 | - | exodeoxyribonuclease X |
| 429 | AA098_10145 | 2258210 | 2259076 | - | 288 | - | ATPase AAA |
| 430 | AA098_10150 | 2259354 | 2259548 | - | 64 | - | hypothetical protein |
| 431 | AA098_10155 | 2259545 | 2259775 | - | 76 | - | hypothetical protein |
| 432 | AA098_10160 | 2259772 | 2260026 | - | 84 | - | hypothetical protein |
| 433 | AA098_10165 | 2260047 | 2260232 | - | 61 | - | hypothetical protein |
| 434 | AA098_10170 | 2260229 | 2260588 | - | 119 | - | hypothetical protein |
| 435 | AA098_10175 | 2260887 | 2261081 | - | 64 | - | hypothetical protein |
| 436 | AA098_10180 | 2261108 | 2261674 | - | 188 | - | hypothetical protein |
| 437 | AA098_10185 | 2261753 | 2262271 | - | 172 | - | hypothetical protein |
| 438 | AA098_10190 | 2262648 | 2262956 | - | 102 | - | hypothetical protein |
| 439 | AA098_10195 | 2263013 | 2263249 | - | 78 | - | hypothetical protein |
| 440 | AA098_10200 | 2263354 | 2263575 | - | 73 | - | hypothetical protein |
| 441 | AA098_10205 | 2263652 | 2264128 | - | 158 | - | hypothetical protein |
| 442 | AA098_10210 | 2264115 | 2265260 | - | 381 | - | peptidase |
| 443 | AA098_10215 | 2265476 | 2265772 | - | 98 | - | prophage PSPPH03, Cro/CI family transcriptional regulator |
| 444 | AA098_10220 | 2265793 | 2266020 | - | 75 | - | prophage PssSM-02 |
| 445 | AA098_10225 | 2266017 | 2266238 | - | 73 | - | peptidase |
| 446 | AA098_10230 | 2266241 | 2266459 | - | 72 | - | hypothetical protein |
| 447 | AA098_10240 | 2266685 | 2267308 | - | 207 | - | cI repressor protein |

| | | | | | | | |
|---|---|---|---|---|---|---|---|
| 448 | AA098_10245 | 2267596 | 2267793 | + | 65 | - | repressor |
| 449 | AA098_10255 | 2268032 | 2268211 | + | 59 | - | hypothetical protein |
| 450 | AA098_10260 | 2268299 | 2269162 | + | 287 | - | transporter |
| 451 | AA098_10265 | 2269159 | 2269641 | + | 160 | - | hypothetical protein |
| 452 | AA098_10270 | 2269638 | 2269874 | + | 78 | - | hypothetical protein |
| 453 | AA098_10275 | 2269871 | 2270647 | + | 258 | - | hypothetical protein |
| 454 | AA098_10280 | 2270644 | 2271405 | + | 253 | - | Replication protein P |
| 455 | AA098_10285 | 2271551 | 2271778 | + | 75 | - | hypothetical protein |
| 456 | AA098_10290 | 2271778 | 2272275 | + | 165 | - | hypothetical protein |
| 457 | AA098_10295 | 2272340 | 2272735 | + | 131 | - | hypothetical protein |
| 458 | AA098_10305 | 2273295 | 2273606 | + | 103 | - | hypothetical protein |
| 459 | AA098_10310 | 2273597 | 2273899 | + | 100 | - | hypothetical protein |
| 460 | AA098_10315 | 2273896 | 2274255 | + | 119 | - | endodeoxyribonuclease RusA |
| 461 | AA098_10320 | 2274395 | 2274940 | + | 181 | - | phage-like protein |
| 462 | AA098_10330 | 2275460 | 2275783 | + | 107 | - | peptidase M48, Ste24p |
| 463 | AA098_10335 | 2275785 | 2276048 | + | 87 | - | hypothetical protein |
| 464 | AA098_10340 | 2276260 | 2276712 | + | 150 | - | proteasome subunit beta |
| 465 | AA098_10360 | 2279100 | 2280518 | + | 472 | - | prophage PSSB64-02, structural protein |
| 466 | AA098_10365 | 2280519 | 2281565 | + | 348 | - | head morphogenesis protein |
| 467 | AA098_10370 | 2281683 | 2282402 | + | 239 | - | hypothetical protein |
| 468 | AA098_10375 | 2282413 | 2283360 | + | 315 | - | major capsid protein |
| 469 | AA098_10380 | 2283404 | 2284021 | + | 205 | - | hypothetical protein |
| 470 | AA098_10385 | 2284061 | 2284453 | + | 130 | - | protein singed |
| 471 | AA098_10390 | 2284456 | 2284839 | + | 127 | - | glutamate 5-kinase |
| 472 | AA098_10395 | 2284839 | 2285219 | + | 126 | - | phage protein |
| 473 | AA098_10400 | 2285216 | 2285638 | + | 140 | - | prophage PSSB64-02 |
| 474 | AA098_10405 | 2285704 | 2286867 | + | 387 | - | hypothetical protein |
| 475 | AA098_10410 | 2286946 | 2287407 | + | 153 | - | prophage PSSB64-02 |

| | | | | | | | |
|---|---|---|---|---|---|---|---|
| 476 | AA098_10415 | 2287437 | 2287682 | + | 81 | - | hypothetical protein |
| 477 | AA098_10425 | 2288528 | 2289514 | + | 328 | - | hypothetical protein |
| 478 | AA098_10430 | 2289511 | 2290011 | + | 166 | - | hypothetical protein |
| 479 | AA098_10435 | 2290102 | 2290386 | + | 94 | - | hypothetical protein |
| 480 | AA098_10440 | 2290422 | 2290745 | + | 107 | - | hypothetical protein |
| 481 | AA098_10445 | 2290807 | 2294994 | + | 1395 | - | tail tape measure protein |
| 482 | AA098_10450 | 2295016 | 2295438 | + | 140 | - | hypothetical protein |
| 483 | AA098_10455 | 2295426 | 2295641 | - | 71 | - | hypothetical protein |
| 484 | AA098_10480 | 2300193 | 2302442 | + | 749 | - | hypothetical protein |
| 485 | AA098_10485 | 2303129 | 2303617 | + | 162 | - | glycoside hydrolase |
| 486 | AA098_10495 | 2304119 | 2305309 | - | 396 | - | integrase |
| 487 | AA098_10505 | 2306845 | 2307153 | + | 102 | - | peptidase |
| 488 | AA098_10580 | 2322429 | 2322962 | + | 177 | - | spore coat protein |
| 489 | AA098_10620 | 2331323 | 2332495 | - | 390 | - | MFS transporter |
| 490 | AA098_10630 | 2333524 | 2337711 | + | 1395 | - | leucine-rich repeat-containing protein |
| 491 | AA098_10685 | 2351592 | 2351819 | - | 75 | - | hypothetical protein |
| 492 | AA098_10730 | 2358603 | 2359499 | - | 298 | - | LysR family transcriptional regulator |
| 493 | AA098_10735 | 2359617 | 2360807 | + | 396 | - | MFS transporter |
| 494 | AA098_10745 | 2363313 | 2367734 | + | 1473 | - | leucine-rich repeat-containing protein |
| 495 | AA098_10750 | 2367832 | 2372322 | + | 1496 | - | hypothetical protein |
| 496 | AA098_10850 | 2391702 | 2392199 | + | 165 | - | acetyltransferase |
| 497 | AA098_10855 | 2392356 | 2392790 | - | 144 | - | blue copper protein |
| 498 | AA098_10860 | 2392808 | 2393596 | - | 262 | - | histidinol phosphatase |
| 499 | AA098_10900 | 2402009 | 2402554 | + | 181 | - | 2'-5' RNA ligase |
| 500 | AA098_10970 | 2415713 | 2417110 | + | 465 | - | hypothetical protein |
| 501 | AA098_10975 | 2417103 | 2417492 | + | 129 | - | hypothetical protein |
| 502 | AA098_11055 | 2430516 | 2431208 | - | 230 | - | deoxyribonuclease |
| 503 | AA098_11060 | 2431215 | 2431457 | - | 80 | - | hypothetical protein |

| | | | | | | | |
|---|---|---|---|---|---|---|---|
| 504 | AA098_11065 | 2431678 | 2432502 | - | 274 | - | formate transporter |
| 505 | AA098_11070 | 2432567 | 2433007 | - | 146 | - | cyanate hydratase |
| 506 | AA098_11075 | 2433209 | 2435083 | + | 624 | - | Fis family transcriptional regulator |
| 507 | AA098_11120 | 2443573 | 2443785 | - | 70 | - | hypothetical protein |
| 508 | AA098_11185 | 2453461 | 2454498 | - | 345 | - | diguanylate cyclase |
| 509 | AA098_11190 | 2454568 | 2454786 | + | 72 | - | hypothetical protein |
| 510 | AA098_11195 | 2454866 | 2455351 | + | 161 | - | membrane protein |
| 511 | AA098_11200 | 2455397 | 2456113 | + | 238 | - | dehydrogenase |
| 512 | AA098_11210 | 2457254 | 2457517 | - | 87 | - | hypothetical protein |
| 513 | AA098_11215 | 2457669 | 2457911 | - | 80 | - | hypothetical protein |
| 514 | AA098_11220 | 2458387 | 2458605 | - | 72 | - | hypothetical protein |
| 515 | AA098_11225 | 2458782 | 2459498 | + | 238 | - | hypothetical protein |
| 516 | AA098_11230 | 2459647 | 2460060 | + | 137 | - | thiol-disulfide oxidoreductase |
| 517 | AA098_11235 | 2460045 | 2460605 | + | 186 | - | GTP cyclohydrolase |
| 518 | AA098_11240 | 2460641 | 2461099 | + | 152 | - | hypothetical protein |
| 519 | AA098_11245 | 2461123 | 2462208 | - | 361 | - | 4-oxalomesaconate tautomerase |
| 520 | AA098_11250 | 2462211 | 2462927 | - | 238 | - | hypothetical protein |
| 521 | AA098_11255 | 2462924 | 2463658 | - | 244 | - | GlcNAc-PI de-N-acetylase |
| 522 | AA098_11260 | 2463818 | 2465011 | + | 397 | - | LysR family transcriptional regulator |
| 523 | AA098_11265 | 2465026 | 2466273 | - | 415 | - | porin |
| 524 | AA098_11270 | 2466405 | 2467667 | - | 420 | - | protocatechuate 3,4-dioxygenase |
| 525 | AA098_11275 | 2467689 | 2469038 | - | 449 | - | 4-hydroxybenzoate transporter |
| 526 | AA098_11285 | 2470378 | 2472789 | - | 803 | - | ligand-gated channel protein |
| 527 | AA098_11290 | 2473043 | 2473699 | + | 218 | - | phosphohydrolase |
| 528 | AA098_11295 | 2473728 | 2474891 | - | 387 | - | ATP-binding protein |
| 529 | AA098_11300 | 2475038 | 2475529 | + | 163 | - | integral membrane protein |
| 530 | AA098_11320 | 2479352 | 2479837 | + | 161 | - | hypothetical protein |
| 531 | AA098_11325 | 2479862 | 2480839 | - | 325 | - | 3-phosphoglycerate dehydrogenase |

| | | | | | | | |
|---|---|---|---|---|---|---|---|
| 532 | AA098_11345 | 2484055 | 2484357 | + | 100 | - | hypothetical protein |
| 533 | AA098_11350 | 2484437 | 2485159 | + | 240 | - | hypothetical protein |
| 534 | AA098_11370 | 2487837 | 2488076 | + | 79 | - | allantoate amidohydrolase |
| 535 | AA098_11375 | 2488067 | 2488342 | - | 91 | - | hypothetical protein |
| 536 | AA098_11390 | 2492283 | 2492462 | - | 59 | - | hypothetical protein |
| 537 | AA098_11465 | 2508441 | 2511212 | - | 923 | - | antibiotic biosynthesis protein |
| 538 | AA098_11480 | 2514825 | 2519624 | - | 1599 | - | leucine-rich repeat-containing protein |
| 539 | AA098_11545 | 2531956 | 2532330 | - | 124 | - | hypothetical protein |
| 540 | AA098_11550 | 2532530 | 2533120 | - | 196 | - | heme oxygenase |
| 541 | AA098_11645 | 2558046 | 2558303 | - | 85 | - | PAAR repeat-containing protein |
| 542 | AA098_11650 | 2558300 | 2559193 | - | 297 | - | hypothetical protein |
| 543 | AA098_11655 | 2559198 | 2560655 | - | 485 | - | hypothetical protein |
| 544 | AA098_11660 | 2560764 | 2561108 | - | 114 | - | hypothetical protein |
| 545 | AA098_11665 | 2561109 | 2561336 | + | 75 | - | hypothetical protein |
| 546 | AA098_11670 | 2561662 | 2564652 | - | 996 | - | hypothetical protein |
| 547 | AA098_11675 | 2564985 | 2565269 | - | 94 | - | hypothetical protein |
| 548 | AA098_11680 | 2565434 | 2565862 | + | 142 | - | membrane protein |
| 549 | AA098_11685 | 2565859 | 2567151 | + | 430 | - | sorbosone dehydrogenase |
| 550 | AA098_11700 | 2570313 | 2571077 | - | 254 | - | hypothetical protein |
| 551 | AA098_11705 | 2571394 | 2571903 | - | 169 | - | MarR family transcriptional regulator |
| 552 | AA098_11710 | 2572099 | 2572617 | + | 172 | - | membrane protein |
| 553 | AA098_11725 | 2574092 | 2574742 | - | 216 | - | hypothetical protein |
| 554 | AA098_11860 | 2610097 | 2611041 | + | 314 | - | sugar ABC transporter substrate-binding protein |
| 555 | AA098_11870 | 2613595 | 2614533 | - | 312 | - | LysR family transcriptional regulator |
| 556 | AA098_11875 | 2614682 | 2615509 | + | 275 | - | 2-pyrone-4,6-dicarboxylate hydrolase |
| 557 | AA098_11880 | 2615687 | 2616427 | + | 246 | - | 3-oxoacyl-ACP reductase |
| 558 | AA098_11930 | 2627332 | 2627943 | - | 203 | - | amino acid transporter LysE |
| 559 | AA098_11975 | 2636841 | 2637074 | + | 77 | - | hypothetical protein |

| | | | | | | | |
|---|---|---|---|---|---|---|---|
| 560 | AA098_12025 | 2649429 | 2650607 | + | 392 | - | integrase |
| 561 | AA098_12035 | 2651707 | 2653773 | - | 688 | - | chemotaxis protein CheY |
| 562 | AA098_12060 | 2658948 | 2659466 | + | 172 | - | methyltransferase |
| 563 | AA098_12095 | 2666660 | 2667712 | - | 350 | - | ornithine cyclodeaminase |
| 564 | AA098_12125 | 2672678 | 2673568 | + | 296 | - | hypothetical protein |
| 565 | AA098_12140 | 2675608 | 2676078 | + | 156 | - | FAD/FMN-containing dehydrogenase |
| 566 | AA098_12150 | 2676436 | 2676762 | - | 108 | - | hypothetical protein |
| 567 | AA098_12165 | 2678395 | 2679114 | - | 239 | - | lipoprotein |
| 568 | AA098_12170 | 2679196 | 2679447 | - | 83 | - | hypothetical protein |
| 569 | AA098_12185 | 2682104 | 2683060 | - | 318 | - | AraC family transcriptional regulator |
| 570 | AA098_12190 | 2683209 | 2685293 | - | 694 | - | 5-oxoprolinase |
| 571 | AA098_12195 | 2685307 | 2687052 | - | 581 | - | hydantoinase |
| 572 | AA098_12220 | 2692242 | 2692604 | - | 120 | - | hypothetical protein |
| 573 | AA098_12385 | 2731068 | 2731304 | + | 78 | - | hypothetical protein |
| 574 | AA098_12390 | 2731826 | 2732401 | - | 191 | - | hypothetical protein |
| 575 | AA098_12395 | 2732770 | 2734539 | - | 589 | - | hypothetical protein |
| 576 | AA098_12400 | 2734634 | 2735476 | - | 280 | - | hypothetical protein |
| 577 | AA098_12405 | 2735473 | 2736048 | - | 191 | - | hypothetical protein |
| 578 | AA098_12430 | 2746732 | 2747409 | + | 225 | - | peptidase S8 and S53 subtilisin kexin sedolisin |
| 579 | AA098_12500 | 2762782 | 2763015 | - | 77 | - | hypothetical protein |
| 580 | AA098_12570 | 2777098 | 2777556 | - | 152 | - | hypothetical protein |
| 581 | AA098_12690 | 2803880 | 2804728 | - | 282 | - | leucyl-tRNA synthetase |
| 582 | AA098_12700 | 2806111 | 2807667 | - | 518 | - | dolichyl-phosphate-mannose-protein mannosyltransferase |
| 583 | AA098_12705 | 2807672 | 2807944 | - | 90 | - | Lipid A biosynthesis, N-terminal |
| 584 | AA098_12710 | 2807941 | 2808684 | - | 247 | - | dolichol-phosphate mannosyltransferase |
| 585 | AA098_12715 | 2808719 | 2809693 | - | 324 | - | protein CapI |
| 586 | AA098_12725 | 2811263 | 2812309 | + | 348 | - | SAM-dependent methyltransferase |
| 587 | AA098_12760 | 2819625 | 2820671 | + | 348 | - | dienelactone hydrolase |

| | | | | | | | |
|---|---|---|---|---|---|---|---|
| 588 | AA098_12775 | 2823621 | 2824040 | - | 139 | - | integrase |
| 589 | AA098_12780 | 2824216 | 2824689 | - | 157 | - | peroxiredoxin |
| 590 | AA098_12785 | 2825249 | 2825518 | - | 89 | - | hypothetical protein |
| 591 | AA098_12790 | 2825554 | 2825811 | + | 85 | - | hypothetical protein |
| 592 | AA098_12800 | 2826870 | 2828234 | - | 454 | - | chemotaxis protein CheY |
| 593 | AA098_12805 | 2828432 | 2829430 | + | 332 | - | cytochrome B6 |
| 594 | AA098_12815 | 2831236 | 2832639 | + | 467 | - | ATPase |
| 595 | AA098_12820 | 2832712 | 2833188 | - | 158 | - | GCN5 family acetyltransferase |
| 596 | AA098_12825 | 2833400 | 2834101 | - | 233 | - | membrane protein |
| 597 | AA098_12830 | 2834171 | 2835136 | - | 321 | - | thiamine biosynthesis protein ApbE |
| 598 | AA098_12835 | 2835126 | 2837306 | - | 726 | - | nitric oxide synthase |
| 599 | AA098_12840 | 2837332 | 2837802 | - | 156 | - | Tat pathway signal protein |
| 600 | AA098_12845 | 2837894 | 2838217 | - | 107 | - | hypothetical protein |
| 601 | AA098_12850 | 2838350 | 2839009 | + | 219 | - | transcriptional regulator |
| 602 | AA098_12855 | 2839006 | 2840340 | + | 444 | - | histidine kinase |
| 603 | AA098_12870 | 2842952 | 2843428 | - | 158 | - | 50S ribosomal protein L21 |
| 604 | AA098_12875 | 2843476 | 2844060 | - | 194 | - | TetR family transcriptional regulator |
| 605 | AA098_12880 | 2844173 | 2845063 | - | 296 | - | LysR family transcriptional regulator |
| 606 | AA098_12890 | 2846355 | 2846681 | + | 108 | - | antibiotic biosynthesis monooxygenase |
| 607 | AA098_12895 | 2846994 | 2847749 | - | 251 | - | hypothetical protein |
| 608 | AA098_12900 | 2847929 | 2848840 | - | 303 | - | magnesium transporter |
| 609 | AA098_12905 | 2848844 | 2850424 | - | 526 | - | carboxylate--amine ligase |
| 610 | AA098_12910 | 2850519 | 2851613 | + | 364 | - | GNAT family acetyltransferase |
| 611 | AA098_12915 | 2851705 | 2851959 | - | 84 | - | glutaredoxin |
| 612 | AA098_12925 | 2852621 | 2853442 | + | 273 | - | AraC family transcriptional regulator |
| 613 | AA098_12935 | 2854491 | 2855492 | + | 333 | - | quinone oxidoreductase |
| 614 | AA098_12940 | 2855723 | 2856238 | - | 171 | - | hypothetical protein |
| 615 | AA098_12955 | 2857730 | 2858209 | + | 159 | - | MerR family transcriptional regulator |

| | | | | | | | |
|---|---|---|---|---|---|---|---|
| 616 | AA098_12960 | 2858225 | 2859151 | - | 308 | - | acetoacetate decarboxylase (ADC) |
| 617 | AA098_12965 | 2859266 | 2860069 | - | 267 | - | thiamine biosynthesis protein ThiJ |
| 618 | AA098_12975 | 2862200 | 2862808 | + | 202 | - | short-chain dehydrogenase |
| 619 | AA098_12980 | 2863151 | 2864044 | - | 297 | - | LysR family transcriptional regulator |
| 620 | AA098_12985 | 2864126 | 2865178 | - | 350 | - | alpha/beta hydrolase |
| 621 | AA098_12990 | 2865228 | 2866292 | - | 354 | - | alpha/beta hydrolase |
| 622 | AA098_12995 | 2866294 | 2867067 | - | 257 | - | carboxymuconolactone decarboxylase |
| 623 | AA098_13000 | 2867297 | 2867764 | - | 155 | - | cupin |
| 624 | AA098_13005 | 2867824 | 2868816 | - | 330 | - | aldehyde oxidase |
| 625 | AA098_13010 | 2868841 | 2869431 | - | 196 | - | flavodoxin |
| 626 | AA098_13015 | 2869428 | 2870417 | - | 329 | - | aldehyde oxidase |
| 627 | AA098_13020 | 2870762 | 2871661 | - | 299 | - | LysR family transcriptional regulator |
| 628 | AA098_13025 | 2871755 | 2872702 | + | 315 | - | 2-dehydropantoate 2-reductase |
| 629 | AA098_13030 | 2872699 | 2873097 | + | 132 | - | glyoxalase |
| 630 | AA098_13035 | 2873094 | 2873549 | + | 151 | - | acyl dehydratase |
| 631 | AA098_13040 | 2873546 | 2874778 | + | 410 | - | CoA-transferase |
| 632 | AA098_13045 | 2874775 | 2875593 | + | 272 | aroE | shikimate dehydrogenase |
| 633 | AA098_13050 | 2875598 | 2876035 | + | 145 | - | 3-dehydroquinate dehydratase |
| 634 | AA098_13055 | 2876176 | 2877573 | + | 465 | - | major facilitator transporter |
| 635 | AA098_13060 | 2877965 | 2878222 | + | 85 | - | hypothetical protein |
| 636 | AA098_13065 | 2878275 | 2878967 | - | 230 | - | RNA polymerase subunit sigma-70 |
| 637 | AA098_13070 | 2878964 | 2879512 | - | 182 | - | RNA polymerase sigma factor |
| 638 | AA098_13075 | 2879616 | 2880143 | - | 175 | - | hypothetical protein |
| 639 | AA098_13080 | 2880361 | 2880648 | + | 95 | - | hypothetical protein |
| 640 | AA098_13095 | 2882154 | 2882354 | - | 66 | - | hypothetical protein |
| 641 | AA098_13110 | 2883455 | 2884255 | + | 266 | - | lipopolysaccharide biosynthesis protein |
| 642 | AA098_13120 | 2884831 | 2885073 | - | 80 | - | hypothetical protein |
| 643 | AA098_13130 | 2886028 | 2886753 | + | 241 | - | serine/threonine protein phosphatase |

| | | | | | | | |
|---|---|---|---|---|---|---|---|
| 644 | AA098_13215 | 2904920 | 2905228 | - | 102 | - | hypothetical protein |
| 645 | AA098_13310 | 2931583 | 2931789 | + | 68 | - | hypothetical protein |
| 646 | AA098_13315 | 2931936 | 2932487 | - | 183 | - | hypothetical protein |
| 647 | AA098_13325 | 2932791 | 2933906 | + | 371 | - | ADP-ribosylglycohydrolase |
| 648 | AA098_13335 | 2934669 | 2934944 | - | 91 | - | hypothetical protein |
| 649 | AA098_13345 | 2935737 | 2936435 | + | 232 | - | hypothetical protein |
| 650 | AA098_13350 | 2936603 | 2936800 | - | 65 | - | hypothetical protein |
| 651 | AA098_13355 | 2936827 | 2937138 | - | 103 | - | hypothetical protein |
| 652 | AA098_13365 | 2937883 | 2938308 | - | 141 | - | hypothetical protein |
| 653 | AA098_13380 | 2940275 | 2940499 | - | 74 | - | hypothetical protein |
| 654 | AA098_13385 | 2940789 | 2941805 | + | 338 | - | hypothetical protein |
| 655 | AA098_13390 | 2941806 | 2942501 | + | 231 | - | hypothetical protein |
| 656 | AA098_13395 | 2942574 | 2943602 | + | 342 | - | transcriptional regulator |
| 657 | AA098_13420 | 2950923 | 2951336 | + | 137 | - | glucan biosynthesis protein |
| 658 | AA098_13425 | 2951821 | 2952573 | + | 250 | - | RHS family protein |
| 659 | AA098_13430 | 2952586 | 2952933 | + | 115 | - | hypothetical protein |
| 660 | AA098_13445 | 2960341 | 2960550 | - | 69 | - | hypothetical protein |
| 661 | AA098_13455 | 2961931 | 2962341 | + | 136 | - | hypothetical protein |
| 662 | AA098_13460 | 2963595 | 2964317 | + | 240 | - | hypothetical protein |
| 663 | AA098_13470 | 2966668 | 2967333 | + | 221 | - | cytoplasmic protein |
| 664 | AA098_13500 | 2972933 | 2973805 | - | 290 | - | AraC family transcriptional regulator |
| 665 | AA098_13505 | 2973982 | 2974809 | + | 275 | - | 2-deoxy-D-gluconate 3-dehydrogenase |
| 666 | AA098_13510 | 2974806 | 2976521 | + | 571 | - | 3-oxosteroid 1-dehydrogenase |
| 667 | AA098_13515 | 2976521 | 2976847 | + | 108 | - | NIPSNAP family containing protein |
| 668 | AA098_13520 | 2976892 | 2978277 | + | 461 | - | 4-hydroxybenzoate transporter |
| 669 | AA098_13575 | 2990086 | 2990628 | + | 180 | - | DNA-binding protein |
| 670 | AA098_13635 | 3005533 | 3006057 | + | 174 | - | serine acetyltransferase |
| 671 | AA098_13660 | 3010490 | 3011242 | + | 250 | - | glycosyl transferase |

| | | | | | | | |
|---|---|---|---|---|---|---|---|
| 672 | AA098_13745 | 3031176 | 3032558 | - | 460 | - | hypothetical protein |
| 673 | AA098_13760 | 3035673 | 3035879 | + | 68 | - | hypothetical protein |
| 674 | AA098_13820 | 3048060 | 3049157 | - | 365 | - | N-ethylmaleimide reductase |
| 675 | AA098_13850 | 3055923 | 3056822 | - | 299 | - | LysR family transcriptional regulator |
| 676 | AA098_13885 | 3066943 | 3067608 | + | 221 | - | hypothetical protein |
| 677 | AA098_13890 | 3067837 | 3069102 | + | 421 | - | cytosine permease |
| 678 | AA098_13895 | 3069099 | 3070316 | + | 405 | - | Fis family transcriptional regulator |
| 679 | AA098_13905 | 3071576 | 3072523 | - | 315 | - | ornithine cyclodeaminase |
| 680 | AA098_13910 | 3072520 | 3073509 | - | 329 | - | hypothetical protein |
| 681 | AA098_13915 | 3073603 | 3074208 | - | 201 | - | hypothetical protein |
| 682 | AA098_13920 | 3074397 | 3075341 | - | 314 | - | Pdr/VanB family oxidoreductase |
| 683 | AA098_13925 | 3075338 | 3076768 | - | 476 | - | amidohydrolase |
| 684 | AA098_13930 | 3076807 | 3077535 | - | 242 | - | ABC transporter ATP-binding protein |
| 685 | AA098_13935 | 3077621 | 3078424 | - | 267 | - | nitrate ABC transporter ATPase |
| 686 | AA098_13940 | 3078535 | 3079560 | - | 341 | - | Rieske (2Fe-2S) protein |
| 687 | AA098_13945 | 3079599 | 3080624 | - | 341 | - | bicyclomycin resistance protein |
| 688 | AA098_13950 | 3081305 | 3082087 | + | 260 | - | membrane protein |
| 689 | AA098_13955 | 3082280 | 3083863 | - | 527 | - | sulfurtransferase |
| 690 | AA098_13960 | 3083860 | 3084468 | - | 202 | - | cysteine dioxygenase |
| 691 | AA098_13965 | 3084765 | 3085703 | + | 312 | - | sulfonate ABC transporter substrate-binding protein |
| 692 | AA098_13970 | 3085762 | 3087081 | + | 439 | - | monooxygenase |
| 693 | AA098_13975 | 3087179 | 3088279 | - | 366 | - | alkanesulfonate monooxygenase |
| 694 | AA098_13980 | 3088420 | 3090039 | - | 539 | - | ABC transporter ATP-binding protein |
| 695 | AA098_13985 | 3090036 | 3090944 | - | 302 | - | ABC transporter permease |
| 696 | AA098_13990 | 3090946 | 3091896 | - | 316 | - | peptide ABC transporter permease |
| 697 | AA098_13995 | 3091893 | 3093512 | - | 539 | - | ABC transporter substrate-binding protein |
| 698 | AA098_14000 | 3093586 | 3094380 | - | 264 | - | aldolase |
| 699 | AA098_14005 | 3094397 | 3095341 | - | 314 | - | dihydrofolate reductase |

| | | | | | | | |
|---|---|---|---|---|---|---|---|
| 700 | AA098_14010 | 3095338 | 3096471 | - | 377 | - | acyl-CoA dehydrogenase |
| 701 | AA098_14015 | 3096666 | 3097556 | - | 296 | - | LysR family transcriptional regulator |
| 702 | AA098_14020 | 3097757 | 3098722 | + | 321 | - | sulfonate ABC transporter substrate-binding protein |
| 703 | AA098_14025 | 3098739 | 3099695 | + | 318 | - | ABC transporter substrate-binding protein |
| 704 | AA098_14035 | 3101150 | 3101401 | - | 83 | - | hypothetical protein |
| 705 | AA098_14050 | 3103675 | 3104178 | - | 167 | - | acetyltransferase |
| 706 | AA098_14085 | 3110188 | 3110370 | + | 60 | - | stress-induced protein |
| 707 | AA098_14090 | 3110429 | 3110626 | + | 65 | - | hypothetical protein |
| 708 | AA098_14095 | 3110645 | 3111976 | + | 443 | - | hypothetical protein |
| 709 | AA098_14100 | 3111976 | 3112929 | + | 317 | - | SAM-dependent methyltransferase |
| 710 | AA098_14130 | 3116882 | 3117637 | - | 251 | - | acetylglucosaminylphosphatidylinositol deacetylase |
| 711 | AA098_14150 | 3121808 | 3122032 | + | 74 | - | metallothionein |
| 712 | AA098_14170 | 3125249 | 3126301 | - | 350 | - | hypothetical protein |
| 713 | AA098_14180 | 3126979 | 3127377 | - | 132 | - | DNA mismatch repair protein MutT |
| 714 | AA098_14280 | 3145920 | 3147158 | - | 412 | - | membrane protein |
| 715 | AA098_14285 | 3147143 | 3147964 | - | 273 | - | Crp/Fnr family transcriptional regulator |
| 716 | AA098_14290 | 3147967 | 3148215 | - | 82 | - | hypothetical protein |
| 717 | AA098_14295 | 3148504 | 3149430 | + | 308 | - | universal stress protein |
| 718 | AA098_14300 | 3149460 | 3150008 | - | 182 | - | GNAT family acetyltransferase |
| 719 | AA098_14305 | 3150191 | 3151117 | + | 308 | - | universal stress protein |
| 720 | AA098_14310 | 3151108 | 3152475 | + | 455 | - | mRNA 3'-end processing factor |
| 721 | AA098_14315 | 3152634 | 3153140 | + | 168 | - | hypothetical protein |
| 722 | AA098_14320 | 3153205 | 3153897 | + | 230 | - | phosphoribosyl transferase |
| 723 | AA098_14325 | 3154047 | 3155414 | - | 455 | - | carboxylic ester hydrolase |
| 724 | AA098_14330 | 3155474 | 3156043 | - | 189 | - | GNAT family acetyltransferase |
| 725 | AA098_14335 | 3156268 | 3156999 | + | 243 | - | transcriptional regulator |
| 726 | AA098_14340 | 3156968 | 3157702 | - | 244 | - | hypothetical protein |
| 727 | AA098_14345 | 3157893 | 3158453 | + | 186 | - | heat-shock protein |

| | | | | | | | |
|---|---|---|---|---|---|---|---|
| 728 | AA098_14350 | 3158783 | 3161158 | + | 791 | - | phosphoketolase |
| 729 | AA098_14355 | 3161166 | 3161591 | - | 141 | - | ion transporter |
| 730 | AA098_14360 | 3161620 | 3163461 | - | 613 | - | cell division protein FtsH |
| 731 | AA098_14370 | 3164323 | 3164577 | + | 84 | - | outer membrane lipoprotein OprI |
| 732 | AA098_14375 | 3164584 | 3166863 | + | 759 | - | ATPase P |
| 733 | AA098_14380 | 3167025 | 3167915 | + | 296 | - | universal stress protein |
| 734 | AA098_14385 | 3168134 | 3169012 | + | 292 | - | universal stress protein |
| 735 | AA098_14390 | 3169014 | 3169586 | - | 190 | - | phosphoribosyltransferase |
| 736 | AA098_14395 | 3169908 | 3171101 | + | 397 | - | sodium:proton antiporter |
| 737 | AA098_14400 | 3171334 | 3171771 | + | 145 | - | YeeE/YedE |
| 738 | AA098_14405 | 3171773 | 3172201 | + | 142 | - | membrane protein |
| 739 | AA098_14410 | 3173936 | 3174616 | + | 226 | - | hypothetical protein |
| 740 | AA098_14415 | 3174742 | 3176634 | + | 630 | - | hypothetical protein |
| 741 | AA098_14420 | 3176644 | 3177636 | + | 330 | - | hypothetical protein |
| 742 | AA098_14425 | 3178140 | 3178334 | - | 64 | - | hypothetical protein |
| 743 | AA098_14435 | 3180058 | 3180375 | + | 105 | - | hypothetical protein |
| 744 | AA098_14440 | 3180378 | 3180782 | + | 134 | - | hypothetical protein |
| 745 | AA098_14445 | 3180868 | 3181599 | + | 243 | - | serine/threonine protein phosphatase |
| 746 | AA098_14450 | 3181661 | 3182056 | + | 131 | - | hypothetical protein |
| 747 | AA098_14455 | 3182074 | 3182367 | - | 97 | - | hypothetical protein |
| 748 | AA098_14460 | 3182549 | 3182842 | + | 97 | - | hypothetical protein |
| 749 | AA098_14465 | 3183292 | 3183486 | - | 64 | - | hypothetical protein |
| 750 | AA098_14470 | 3183625 | 3183918 | + | 97 | - | hypothetical protein |
| 751 | AA098_14475 | 3184130 | 3184354 | + | 74 | - | hypothetical protein |
| 752 | AA098_14480 | 3184453 | 3184803 | - | 116 | - | hypothetical protein |
| 753 | AA098_14485 | 3184872 | 3185312 | + | 146 | - | histidine kinase |
| 754 | AA098_14490 | 3185404 | 3185682 | - | 92 | - | amino acid transporter |
| 755 | AA098_14495 | 3185764 | 3186228 | - | 154 | - | hemerythrin |

| | | | | | | | |
|---|---|---|---|---|---|---|---|
| 756 | AA098_14500 | 3186334 | 3188088 | - | 584 | - | glycosyl hydrolase |
| 757 | AA098_14505 | 3188346 | 3188579 | + | 77 | - | hypothetical protein |
| 758 | AA098_14510 | 3188603 | 3189046 | - | 147 | - | membrane protein |
| 759 | AA098_14515 | 3189057 | 3189509 | - | 150 | - | hypothetical protein |
| 760 | AA098_14520 | 3189575 | 3190078 | - | 167 | - | damage-inducible protein CinA |
| 761 | AA098_14525 | 3190462 | 3191319 | - | 285 | - | dehydrogenase |
| 762 | AA098_14530 | 3191515 | 3192039 | + | 174 | - | membrane protein |
| 763 | AA098_14535 | 3192098 | 3192637 | - | 179 | - | glutamine amidotransferase |
| 764 | AA098_14540 | 3192721 | 3194049 | - | 442 | - | short-chain dehydrogenase |
| 765 | AA098_14545 | 3194083 | 3194316 | - | 77 | - | hypothetical protein |
| 766 | AA098_14550 | 3194330 | 3195532 | - | 400 | - | alcohol dehydrogenase |
| 767 | AA098_14555 | 3196449 | 3196682 | + | 77 | - | hypothetical protein |
| 768 | AA098_14560 | 3196728 | 3197930 | - | 400 | - | histidine kinase |
| 769 | AA098_14565 | 3197980 | 3198735 | - | 251 | - | sigma factor sigB regulation protein rsbQ |
| 770 | AA098_14570 | 3198956 | 3199432 | + | 158 | - | hypothetical protein |
| 771 | AA098_14575 | 3199572 | 3199862 | + | 96 | - | hypothetical protein |
| 772 | AA098_14580 | 3199901 | 3200137 | - | 78 | - | hypothetical protein |
| 773 | AA098_14595 | 3203735 | 3204025 | + | 96 | - | hypothetical protein |
| 774 | AA098_14600 | 3204151 | 3204543 | - | 130 | - | stress-induced protein |
| 775 | AA098_14605 | 3204725 | 3204931 | - | 68 | - | hypothetical protein |
| 776 | AA098_14610 | 3205375 | 3207819 | + | 814 | - | hypothetical protein |
| 777 | AA098_14615 | 3207865 | 3208263 | - | 132 | - | hypothetical protein |
| 778 | AA098_14620 | 3208281 | 3209867 | - | 528 | - | phospholipase |
| 779 | AA098_14630 | 3211480 | 3211710 | + | 76 | - | hypothetical protein |
| 780 | AA098_14635 | 3211770 | 3212462 | - | 230 | - | membrane protein |
| 781 | AA098_14640 | 3212756 | 3213124 | + | 122 | - | transcriptional regulator |
| 782 | AA098_14645 | 3213941 | 3214174 | + | 77 | - | hypothetical protein |
| 783 | AA098_14650 | 3214221 | 3214607 | - | 128 | - | DNA-binding protein |

| | | | | | | | |
|---|---|---|---|---|---|---|---|
| 784 | AA098_14655 | 3214799 | 3215047 | + | 82 | - | hypothetical protein |
| 785 | AA098_14660 | 3215237 | 3216595 | + | 452 | - | ATPase |
| 786 | AA098_14665 | 3216616 | 3217791 | + | 391 | - | restriction endonuclease |
| 787 | AA098_14670 | 3218254 | 3219168 | + | 304 | - | hypothetical protein |
| 788 | AA098_14675 | 3219287 | 3219517 | - | 76 | - | hypothetical protein |
| 789 | AA098_14680 | 3219899 | 3220165 | - | 88 | - | hypothetical protein |
| 790 | AA098_14685 | 3220301 | 3220510 | + | 69 | - | hypothetical protein |
| 791 | AA098_14690 | 3221506 | 3224310 | + | 934 | - | hypothetical protein |
| 792 | AA098_14695 | 3224583 | 3225524 | - | 313 | - | recombinase |
| 793 | AA098_14700 | 3225729 | 3226721 | + | 330 | - | integrase |
| 794 | AA098_14705 | 3226993 | 3230916 | + | 1307 | - | hypothetical protein |
| 795 | AA098_14710 | 3230913 | 3232997 | + | 694 | - | hypothetical protein |
| 796 | AA098_14715 | 3233703 | 3235307 | - | 534 | - | hypothetical protein |
| 797 | AA098_14720 | 3235590 | 3237005 | - | 471 | - | helicase UvrD |
| 798 | AA098_14725 | 3236995 | 3238725 | - | 576 | - | chromosome segregation protein SMC |
| 799 | AA098_14730 | 3239041 | 3239382 | - | 113 | - | DNA-binding protein |
| 800 | AA098_14735 | 3239733 | 3241700 | + | 655 | - | hypothetical protein |
| 801 | AA098_14745 | 3243899 | 3244906 | + | 335 | - | hypothetical protein |
| 802 | AA098_14750 | 3245041 | 3245352 | - | 103 | - | hypothetical protein |
| 803 | AA098_14755 | 3245376 | 3246311 | - | 311 | - | hypothetical protein |
| 804 | AA098_14760 | 3247039 | 3247368 | + | 109 | - | hypothetical protein |
| 805 | AA098_14765 | 3247352 | 3247651 | + | 99 | - | hypothetical protein |
| 806 | AA098_14770 | 3248550 | 3248771 | - | 73 | - | hypothetical protein |
| 807 | AA098_14780 | 3249876 | 3250061 | - | 61 | - | hypothetical protein |
| 808 | AA098_14785 | 3250400 | 3251500 | + | 366 | - | NADH:flavin oxidoreductase |
| 809 | AA098_14790 | 3251631 | 3252554 | - | 307 | - | LysR family transcriptional regulator |
| 810 | AA098_14820 | 3258100 | 3259470 | - | 456 | - | alpha-ketoglutarate permease |
| 811 | AA098_14825 | 3259745 | 3260668 | - | 307 | - | LysR family transcriptional regulator |

| | | | | | | | |
|---|---|---|---|---|---|---|---|
| 812 | AA098_14830 | 3260883 | 3262175 | - | 430 | - | C4-dicarboxylate ABC transporter |
| 813 | AA098_14835 | 3262664 | 3263194 | + | 176 | - | phosphonopyruvate decarboxylase |
| 814 | AA098_14840 | 3263182 | 3263778 | + | 198 | - | aldehyde dehydrogenase |
| 815 | AA098_14845 | 3263850 | 3265013 | + | 387 | - | acyl-CoA dehydrogenase |
| 816 | AA098_14850 | 3265069 | 3266265 | + | 398 | - | CoA-transferase |
| 817 | AA098_14860 | 3267066 | 3267995 | + | 309 | - | electron transfer flavoprotein subunit beta |
| 818 | AA098_14865 | 3268009 | 3268851 | + | 280 | - | transposase |
| 819 | AA098_14870 | 3268848 | 3269663 | + | 271 | - | host specificity protein |
| 820 | AA098_14875 | 3269848 | 3270426 | + | 192 | - | hypothetical protein |
| 821 | AA098_14880 | 3270502 | 3271545 | - | 347 | - | 3-phosphoglycerate dehydrogenase |
| 822 | AA098_14885 | 3271558 | 3272925 | - | 455 | - | alpha-ketoglutarate permease |
| 823 | AA098_14890 | 3273293 | 3274405 | - | 370 | - | MFS transporter |
| 824 | AA098_14895 | 3274734 | 3275396 | - | 220 | - | diguanylate cyclase |
| 825 | AA098_14900 | 3275450 | 3276799 | - | 449 | - | DgoT |
| 826 | AA098_14905 | 3276951 | 3277850 | + | 299 | - | LysR family transcriptional regulator |
| 827 | AA098_14910 | 3277953 | 3278618 | + | 221 | - | dimethylmenaquinone methyltransferase |
| 828 | AA098_14915 | 3278777 | 3279676 | - | 299 | - | GntR family transcriptional regulator |
| 829 | AA098_14920 | 3279790 | 3280980 | + | 396 | - | CoA-transferase |
| 830 | AA098_14925 | 3280998 | 3281963 | + | 321 | - | hydroxymethylglutaryl-CoA lyase |
| 831 | AA098_14930 | 3281960 | 3282349 | + | 129 | - | lactoylglutathione lyase |
| 832 | AA098_14935 | 3282364 | 3283305 | + | 313 | - | 2-hydroxyacid dehydrogenase |
| 833 | AA098_14940 | 3283343 | 3284641 | + | 432 | - | C4-dicarboxylate transporter |
| 834 | AA098_14945 | 3284849 | 3286057 | - | 402 | - | aspartate aminotransferase |
| 835 | AA098_14950 | 3286054 | 3286683 | - | 209 | - | dimethylmenaquinone methyltransferase |
| 836 | AA098_14955 | 3286680 | 3287345 | - | 221 | - | diguanylate cyclase |
| 837 | AA098_14960 | 3287347 | 3288009 | - | 220 | - | diguanylate cyclase |
| 838 | AA098_14965 | 3288117 | 3289013 | + | 298 | - | LysR family transcriptional regulator |
| 839 | AA098_14970 | 3289104 | 3289913 | + | 269 | - | alpha/beta hydrolase |

| | | | | | | | |
|---|---|---|---|---|---|---|---|
| 840 | AA098_14975 | 3289997 | 3291433 | + | 478 | - | sodium:alanine symporter |
| 841 | AA098_14980 | 3291752 | 3292504 | - | 250 | - | oxidoreductase |
| 842 | AA098_14985 | 3292608 | 3293516 | + | 302 | - | LysR family transcriptional regulator |
| 843 | AA098_14990 | 3293908 | 3294351 | + | 147 | - | metal transporter |
| 844 | AA098_14995 | 3294378 | 3296057 | - | 559 | - | sulfate transporter |
| 845 | AA098_15000 | 3296575 | 3297786 | + | 403 | - | hemolysin D |
| 846 | AA098_15005 | 3297783 | 3300992 | + | 1069 | - | RND transporter |
| 847 | AA098_15010 | 3300994 | 3302427 | + | 477 | - | RND transporter |
| 848 | AA098_15015 | 3302780 | 3304138 | + | 452 | - | hypothetical protein |
| 849 | AA098_15020 | 3304225 | 3305337 | + | 370 | - | hypothetical protein |
| 850 | AA098_15025 | 3305723 | 3307063 | + | 446 | - | putrescine transporter |
| 851 | AA098_15030 | 3307112 | 3308020 | + | 302 | - | ABC transporter substrate-binding protein |
| 852 | AA098_15035 | 3308244 | 3308606 | - | 120 | - | transcriptional regulator |
| 853 | AA098_15040 | 3309106 | 3309519 | - | 137 | - | peptidyl-tRNA hydrolase |
| 854 | AA098_15045 | 3309711 | 3309842 | - | 43 | - | DNA-directed DNA polymerase |
| 855 | AA098_15055 | 3310302 | 3310484 | + | 60 | - | hypothetical protein |
| 856 | AA098_15060 | 3310630 | 3311574 | + | 314 | - | GTP cyclohydrolase |
| 857 | AA098_15065 | 3311883 | 3313973 | + | 696 | - | TonB-dependent receptor |
| 858 | AA098_15070 | 3313988 | 3314710 | + | 240 | - | nickel ABC transporter substrate-binding protein |
| 859 | AA098_15075 | 3314913 | 3316229 | - | 438 | - | sodium:dicarboxylate symporter |
| 860 | AA098_15080 | 3316388 | 3316801 | - | 137 | - | ring-cleaving dioxygenase |
| 861 | AA098_15085 | 3316903 | 3317796 | + | 297 | - | LysR family transcriptional regulator |
| 862 | AA098_15090 | 3318199 | 3318588 | + | 129 | - | hypothetical protein |
| 863 | AA098_15095 | 3318660 | 3320813 | + | 717 | - | TonB-dependent receptor |
| 864 | AA098_15100 | 3321188 | 3321595 | + | 135 | - | hypothetical protein |
| 865 | AA098_15105 | 3321677 | 3323728 | + | 683 | - | TonB-dependent receptor |
| 866 | AA098_15110 | 3324206 | 3325264 | + | 352 | - | diguanylate cyclase |
| 867 | AA098_15120 | 3326771 | 3329818 | - | 1015 | - | hypothetical protein |

| | | | | | | | |
|---|---|---|---|---|---|---|---|
| 868 | AA098_15125 | 3329962 | 3330402 | + | 146 | - | AsnC family transcriptional regulator |
| 869 | AA098_15135 | 3331287 | 3333830 | + | 847 | - | chemotaxis protein CheY |
| 870 | AA098_15150 | 3336652 | 3337311 | - | 219 | - | alpha/beta hydrolase |
| 871 | AA098_15160 | 3338826 | 3339677 | + | 283 | - | LysR family transcriptional regulator |
| 872 | AA098_15165 | 3339774 | 3340769 | + | 331 | - | oxidoreductase |
| 873 | AA098_15170 | 3340762 | 3342024 | - | 420 | - | histidine kinase |
| 874 | AA098_15175 | 3342021 | 3342722 | - | 233 | - | chemotaxis protein CheY |
| 875 | AA098_15185 | 3345414 | 3345797 | + | 127 | - | hypothetical protein |
| 876 | AA098_15235 | 3356267 | 3356956 | + | 229 | - | phosphohistidine phosphatase |
| 877 | AA098_15240 | 3356959 | 3357795 | + | 278 | - | phosphatidic acid phosphatase |
| 878 | AA098_15255 | 3361052 | 3361930 | + | 292 | - | hypothetical protein |
| 879 | AA098_15260 | 3361945 | 3364617 | + | 890 | - | hypothetical protein |
| 880 | AA098_15335 | 3379509 | 3379988 | + | 159 | - | GNAT family acetyltransferase |
| 881 | AA098_15395 | 3391735 | 3392037 | + | 100 | - | hypothetical protein |
| 882 | AA098_15455 | 3408296 | 3410359 | - | 687 | - | histidine kinase |
| 883 | AA098_15465 | 3411369 | 3412175 | + | 268 | - | hypothetical protein |
| 884 | AA098_15505 | 3420698 | 3421852 | - | 384 | - | hypothetical protein |
| 885 | AA098_15595 | 3440878 | 3441633 | - | 251 | - | aldolase |
| 886 | AA098_15655 | 3453149 | 3453718 | + | 189 | - | N-acetylglucosamine-6-sulfatase |
| 887 | AA098_15660 | 3454016 | 3454453 | + | 145 | - | hypothetical protein |
| 888 | AA098_15820 | 3491847 | 3493562 | + | 571 | - | hypothetical protein |
| 889 | AA098_15825 | 3493728 | 3497510 | + | 1260 | - | hypothetical protein |
| 890 | AA098_15830 | 3497507 | 3498124 | + | 205 | - | hypothetical protein |
| 891 | AA098_15835 | 3498121 | 3499359 | + | 412 | - | hypothetical protein |
| 892 | AA098_15840 | 3499347 | 3500189 | + | 280 | - | hypothetical protein |
| 893 | AA098_15845 | 3500555 | 3501568 | - | 337 | - | hypothetical protein |
| 894 | AA098_15850 | 3501640 | 3501834 | - | 64 | - | hypothetical protein |
| 895 | AA098_15855 | 3502110 | 3502868 | - | 252 | - | hypothetical protein |

| | | | | | | | |
|---|---|---|---|---|---|---|---|
| 896 | AA098_15860 | 3503017 | 3503334 | + | 105 | - | AbrB family transcriptional regulator |
| 897 | AA098_15875 | 3504195 | 3505013 | - | 272 | - | dihydrodipicolinate reductase |
| 898 | AA098_15880 | 3505961 | 3506539 | + | 192 | - | NADPH-quinone reductase |
| 899 | AA098_15885 | 3506706 | 3507089 | + | 127 | - | MerR family transcriptional regulator |
| 900 | AA098_15890 | 3507159 | 3509768 | - | 869 | - | transporter |
| 901 | AA098_15895 | 3509802 | 3510638 | - | 278 | - | sigma E regulatory protein, MucB/RseB |
| 902 | AA098_15900 | 3510689 | 3511972 | - | 427 | - | hypothetical protein |
| 903 | AA098_15905 | 3511975 | 3512739 | - | 254 | - | short-chain dehydrogenase |
| 904 | AA098_15910 | 3512736 | 3513473 | - | 245 | - | 3-oxoacyl-ACP reductase |
| 905 | AA098_15915 | 3513483 | 3516353 | - | 956 | - | pyridoxalphosphate dependent aminotransferase |
| 906 | AA098_15920 | 3516392 | 3517603 | - | 403 | - | beta-ketoacyl synthase |
| 907 | AA098_15925 | 3517600 | 3518898 | - | 432 | - | 3-oxoacyl-ACP synthase |
| 908 | AA098_15930 | 3518895 | 3519809 | - | 304 | - | beta-ketoacyl synthase |
| 909 | AA098_15935 | 3519979 | 3521253 | - | 424 | - | 3-oxoacyl-ACP synthase |
| 910 | AA098_15940 | 3521265 | 3521834 | - | 189 | - | acyl carrier protein |
| 911 | AA098_15950 | 3522944 | 3523366 | + | 140 | - | hypothetical protein |
| 912 | AA098_15955 | 3523413 | 3524600 | + | 395 | - | monooxygenase |
| 913 | AA098_15960 | 3524609 | 3525712 | + | 367 | - | Fis family transcriptional regulator |
| 914 | AA098_15965 | 3525925 | 3527250 | - | 441 | - | ABC transporter permease |
| 915 | AA098_15970 | 3527318 | 3528385 | - | 355 | - | ABC transporter permease |
| 916 | AA098_15975 | 3528398 | 3529279 | - | 293 | - | ABC transporter permease |
| 917 | AA098_15980 | 3529255 | 3530088 | - | 277 | - | ABC transporter |
| 918 | AA098_15985 | 3530098 | 3531228 | - | 376 | - | long-chain fatty acid--CoA ligase |
| 919 | AA098_15990 | 3531240 | 3532331 | - | 363 | - | alkanesulfonate monooxygenase |
| 920 | AA098_15995 | 3532358 | 3532918 | - | 186 | - | FMN reductase |
| 921 | AA098_16000 | 3533257 | 3534495 | + | 412 | - | monooxygenase |
| 922 | AA098_16005 | 3534561 | 3535547 | - | 328 | - | branched-chain amino acid ABC transporter permease |
| 923 | AA098_16010 | 3535549 | 3536526 | - | 325 | - | ABC transporter permease |

| | | | | | | | |
|---|---|---|---|---|---|---|---|
| 924 | AA098_16015 | 3536510 | 3538048 | - | 512 | - | sugar ABC transporter |
| 925 | AA098_16020 | 3538045 | 3539073 | - | 342 | - | sugar ABC transporter substrate-binding protein |
| 926 | AA098_16025 | 3539070 | 3540041 | - | 323 | - | sugar ABC transporter substrate-binding protein |
| 927 | AA098_16030 | 3540038 | 3540424 | - | 128 | - | hypothetical protein |
| 928 | AA098_16035 | 3540434 | 3541666 | - | 410 | - | acyl-CoA dehydrogenase |
| 929 | AA098_16040 | 3542012 | 3543361 | - | 449 | - | porin |
| 930 | AA098_16045 | 3543645 | 3544421 | - | 258 | - | branched-chain amino acid ABC transporter ATP-binding protein |
| 931 | AA098_16050 | 3544538 | 3545872 | - | 444 | - | ABC transporter permease |
| 932 | AA098_16055 | 3545932 | 3547272 | - | 446 | - | ABC transporter permease |
| 933 | AA098_16060 | 3547351 | 3548415 | - | 354 | - | ABC transporter permease |
| 934 | AA098_16065 | 3548420 | 3549301 | - | 293 | - | ABC transporter permease |
| 935 | AA098_16070 | 3549298 | 3550098 | - | 266 | - | ABC transporter |
| 936 | AA098_16075 | 3550065 | 3551036 | - | 323 | - | hypothetical protein |
| 937 | AA098_16080 | 3551057 | 3552466 | - | 469 | - | 5,10-methylene tetrahydromethanopterin reductase |
| 938 | AA098_16085 | 3552710 | 3553699 | + | 329 | - | universal stress protein |
| 939 | AA098_16090 | 3553795 | 3554754 | + | 319 | - | ribose-phosphate pyrophosphokinase |
| 940 | AA098_16095 | 3554936 | 3555544 | + | 202 | - | acetyltransferase |
| 941 | AA098_16100 | 3555541 | 3555900 | + | 119 | - | cupin |
| 942 | AA098_16105 | 3555968 | 3557605 | + | 545 | - | mechanosensitive ion channel protein MscS |
| 943 | AA098_16120 | 3559402 | 3559824 | + | 140 | - | transcriptional regulator |
| 944 | AA098_16155 | 3566010 | 3566315 | - | 101 | - | metal ABC transporter ATPase |
| 945 | AA098_16160 | 3566312 | 3566848 | - | 178 | - | lipoprotein |
| 946 | AA098_16165 | 3566851 | 3567387 | - | 178 | - | periplasmic protein |
| 947 | AA098_16170 | 3567548 | 3568039 | + | 163 | - | hypothetical protein |
| 948 | AA098_16175 | 3568118 | 3568888 | + | 256 | - | C-factor |
| 949 | AA098_16180 | 3568984 | 3569676 | + | 230 | - | membrane protein |
| 950 | AA098_16185 | 3569769 | 3570308 | + | 179 | - | glutamine amidotransferase |
| 951 | AA098_16190 | 3570365 | 3570889 | - | 174 | - | membrane protein |

| | | | | | | | |
|---|---|---|---|---|---|---|---|
| 952 | AA098_16195 | 3571084 | 3571941 | + | 285 | - | dehydrogenase |
| 953 | AA098_16200 | 3572229 | 3572456 | - | 75 | - | hypothetical protein |
| 954 | AA098_16205 | 3572922 | 3573269 | + | 115 | - | ArsR family transcriptional regulator |
| 955 | AA098_16210 | 3573291 | 3574574 | + | 427 | - | arsenical pump membrane protein |
| 956 | AA098_16215 | 3574602 | 3575072 | + | 156 | - | ArsC family transcriptional regulator |
| 957 | AA098_16225 | 3575800 | 3577164 | - | 454 | - | histidine kinase |
| 958 | AA098_16240 | 3578464 | 3579270 | - | 268 | - | short-chain dehydrogenase |
| 959 | AA098_16250 | 3579946 | 3581415 | - | 489 | - | long-chain acyl-CoA synthetase |
| 960 | AA098_16255 | 3581405 | 3582085 | - | 226 | - | thermostable hemolysin |
| 961 | AA098_16265 | 3582996 | 3583238 | - | 80 | - | hypothetical protein |
| 962 | AA098_16270 | 3583305 | 3583562 | - | 85 | - | hypothetical protein |
| 963 | AA098_16275 | 3583691 | 3584875 | - | 394 | - | amidohydrolase |
| 964 | AA098_16280 | 3584877 | 3586178 | - | 433 | - | MFS transporter |
| 965 | AA098_16285 | 3586552 | 3587682 | + | 376 | - | porin |
| 966 | AA098_16290 | 3587659 | 3588597 | - | 312 | - | LysR family transcriptional regulator |
| 967 | AA098_16295 | 3588684 | 3589184 | - | 166 | - | peroxiredoxin |
| 968 | AA098_16300 | 3589416 | 3590399 | + | 327 | - | hypothetical protein |
| 969 | AA098_16305 | 3590426 | 3591460 | + | 344 | - | 5-methyltetrahydropteroyltriglutamate--homocysteine methyltransferase |
| 970 | AA098_16310 | 3591486 | 3591995 | + | 169 | - | flavin reductase |
| 971 | AA098_16315 | 3592094 | 3593023 | + | 309 | - | XRE family transcriptional regulator |
| 972 | AA098_16320 | 3593090 | 3593977 | - | 295 | - | LysR family transcriptional regulator |
| 973 | AA098_16325 | 3594073 | 3595515 | + | 480 | - | aldehyde dehydrogenase |
| 974 | AA098_16330 | 3595529 | 3596515 | + | 328 | - | peptidase M19 |
| 975 | AA098_16335 | 3596571 | 3598121 | + | 516 | - | BCCT transporter |
| 976 | AA098_16340 | 3598148 | 3599842 | + | 564 | - | hypothetical protein |
| 977 | AA098_16345 | 3599845 | 3600540 | + | 231 | - | lactate dehydrogenase |
| 978 | AA098_16350 | 3600568 | 3600939 | + | 123 | - | endoribonuclease |
| 979 | AA098_16355 | 3601373 | 3602782 | + | 469 | - | hypothetical protein |

| | | | | | | | |
|---|---|---|---|---|---|---|---|
| 980 | AA098_16360 | 3602786 | 3603736 | + | 316 | - | hypothetical protein |
| 981 | AA098_16365 | 3603733 | 3604512 | + | 259 | - | transglutaminase |
| 982 | AA098_16370 | 3604586 | 3605308 | + | 240 | - | peptidase |
| 983 | AA098_16375 | 3605723 | 3607066 | + | 447 | - | permease DsdX |
| 984 | AA098_16380 | 3607101 | 3608450 | + | 449 | - | D-serine dehydratase |
| 985 | AA098_16385 | 3608486 | 3609424 | + | 312 | - | transcriptional regulator |
| 986 | AA098_16390 | 3609454 | 3610821 | - | 455 | - | GntR family transcriptional regulator |
| 987 | AA098_16395 | 3610972 | 3612258 | + | 428 | - | 3-hydroxy-3-methylglutaryl-CoA reductase |
| 988 | AA098_16400 | 3612255 | 3613175 | + | 306 | - | hydroxymethylglutaryl-CoA lyase |
| 989 | AA098_16405 | 3613278 | 3613730 | + | 150 | - | hypothetical protein |
| 990 | AA098_16410 | 3613727 | 3615112 | + | 461 | - | membrane protein |
| 991 | AA098_16420 | 3616580 | 3617194 | - | 204 | - | hypothetical protein |
| 992 | AA098_16435 | 3620739 | 3621011 | - | 90 | - | pyrroloquinoline quinone biosynthesis protein PqqD |
| 993 | AA098_16465 | 3627800 | 3628261 | - | 153 | - | cytochrome C |
| 994 | AA098_16510 | 3637486 | 3638007 | + | 173 | - | transposase |
| 995 | AA098_16515 | 3638037 | 3638840 | + | 267 | - | integrase |
| 996 | AA098_16575 | 3650627 | 3650860 | - | 77 | - | hypothetical protein |
| 997 | AA098_16580 | 3650932 | 3651357 | - | 141 | - | glutathione metabolism protein |
| 998 | AA098_16585 | 3651354 | 3651668 | - | 104 | - | hypothetical protein |
| 999 | AA098_16590 | 3651775 | 3652593 | + | 272 | - | Cro/Cl family transcriptional regulator |
| 1000 | AA098_16595 | 3652583 | 3653368 | - | 261 | - | hydratase |
| 1001 | AA098_16605 | 3654895 | 3656037 | - | 380 | - | 4-hydroxybutyrate dehydrogenase |
| 1002 | AA098_16610 | 3656180 | 3657121 | + | 313 | - | LysR family transcriptional regulator |
| 1003 | AA098_16615 | 3657215 | 3657673 | - | 152 | - | AsnC family transcriptional regulator |
| 1004 | AA098_16620 | 3657799 | 3658515 | + | 238 | - | branched-chain amino acid ABC transporter permease |
| 1005 | AA098_16625 | 3658512 | 3658814 | + | 100 | - | membrane protein |
| 1006 | AA098_16640 | 3661469 | 3661846 | - | 125 | - | hypothetical protein |
| 1007 | AA098_16645 | 3661868 | 3664582 | - | 904 | - | magnesium ABC transporter ATPase |

| | | | | | | | |
|---|---|---|---|---|---|---|---|
| 1008 | AA098_16665 | 3670556 | 3671014 | + | 152 | - | acetyltransferase |
| 1009 | AA098_16675 | 3672372 | 3673094 | - | 240 | - | hypothetical protein |
| 1010 | AA098_16680 | 3673112 | 3674575 | - | 487 | - | peptidase |
| 1011 | AA098_16690 | 3676809 | 3680426 | - | 1205 | - | type VI secretion protein VasK |
| 1012 | AA098_16695 | 3680423 | 3681886 | - | 487 | - | membrane protein |
| 1013 | AA098_16700 | 3681874 | 3682317 | - | 147 | - | hypothetical protein |
| 1014 | AA098_16705 | 3682569 | 3683597 | - | 342 | - | transcriptional regulator |
| 1015 | AA098_16710 | 3683967 | 3684470 | + | 167 | - | type VI secretion protein |
| 1016 | AA098_16715 | 3684488 | 3685978 | + | 496 | - | type VI secretion protein |
| 1017 | AA098_16720 | 3685975 | 3686385 | + | 136 | - | hypothetical protein |
| 1018 | AA098_16725 | 3686389 | 3688155 | + | 588 | - | type VI secretion protein |
| 1019 | AA098_16730 | 3688119 | 3689135 | + | 338 | - | type VI secretion protein |
| 1020 | AA098_16735 | 3689132 | 3689701 | + | 189 | - | hypothetical protein |
| 1021 | AA098_16740 | 3689698 | 3690495 | + | 265 | - | hypothetical protein |
| 1022 | AA098_16745 | 3690551 | 3691888 | + | 445 | - | type VI secretion protein |
| 1023 | AA098_16755 | 3692886 | 3693401 | + | 171 | - | major exported protein |
| 1024 | AA098_16765 | 3695641 | 3696564 | + | 307 | - | hypothetical protein |
| 1025 | AA098_16770 | 3696557 | 3699547 | + | 996 | - | hypothetical protein |
| 1026 | AA098_16775 | 3699904 | 3700440 | + | 178 | - | hypothetical protein |
| 1027 | AA098_16780 | 3700928 | 3701485 | + | 185 | - | hypothetical protein |
| 1028 | AA098_16835 | 3713279 | 3714622 | - | 447 | - | porin |
| 1029 | AA098_16890 | 3724014 | 3724553 | - | 179 | - | transcriptional regulator |
| 1030 | AA098_16900 | 3726180 | 3727805 | + | 541 | - | chemotaxis protein |
| 1031 | AA098_16980 | 3743085 | 3744404 | - | 439 | - | porin |
| 1032 | AA098_16985 | 3744509 | 3745237 | - | 242 | - | nitrobenzoate reductase |
| 1033 | AA098_16990 | 3745266 | 3746618 | - | 450 | - | major facilitator transporter |
| 1034 | AA098_16995 | 3746885 | 3747910 | - | 341 | - | AraC family transcriptional regulator |
| 1035 | AA098_17000 | 3748142 | 3748597 | + | 151 | - | dehydratase |

| | | | | | | | |
|---|---|---|---|---|---|---|---|
| 1036 | AA098_17030 | 3755470 | 3756477 | + | 335 | - | AraC family transcriptional regulator |
| 1037 | AA098_17035 | 3756602 | 3757936 | - | 444 | - | MFS transporter |
| 1038 | AA098_17070 | 3767302 | 3767796 | - | 164 | - | acetyltransferase |
| 1039 | AA098_17195 | 3797264 | 3798331 | + | 355 | - | Rieske (2Fe-2S) protein |
| 1040 | AA098_17200 | 3798344 | 3799294 | + | 316 | - | Vanillate O-demethylase oxidoreductase |
| 1041 | AA098_17205 | 3799301 | 3800014 | - | 237 | - | GntR family transcriptional regulator |
| 1042 | AA098_17210 | 3800160 | 3801413 | - | 417 | - | porin |
| 1043 | AA098_17215 | 3801516 | 3802856 | - | 446 | - | MFS transporter |
| 1044 | AA098_17230 | 3806264 | 3806581 | + | 105 | - | hypothetical protein |
| 1045 | AA098_17260 | 3811928 | 3812266 | + | 112 | - | hypothetical protein |
| 1046 | AA098_17350 | 3830486 | 3830728 | + | 80 | - | hypothetical protein |
| 1047 | AA098_17360 | 3831768 | 3832121 | + | 117 | - | hypothetical protein |
| 1048 | AA098_17370 | 3832488 | 3832802 | - | 104 | - | chemotaxis protein |
| 1049 | AA098_17380 | 3833830 | 3834153 | - | 107 | - | hypothetical protein |
| 1050 | AA098_17385 | 3834388 | 3835062 | + | 224 | - | alginate lyase |
| 1051 | AA098_17390 | 3835176 | 3835409 | - | 77 | - | hypothetical protein |
| 1052 | AA098_17395 | 3836574 | 3836909 | + | 111 | - | hypothetical protein |
| 1053 | AA098_17400 | 3837056 | 3837373 | - | 105 | - | hypothetical protein |
| 1054 | AA098_17570 | 3867973 | 3868731 | + | 252 | - | molybdate ABC transporter substrate-binding protein |
| 1055 | AA098_17595 | 3872074 | 3872427 | - | 117 | - | chemotaxis protein CheY |
| 1056 | AA098_17620 | 3876122 | 3876316 | - | 64 | - | hypothetical protein |
| 1057 | AA098_17630 | 3877854 | 3878060 | + | 68 | - | hypothetical protein |
| 1058 | AA098_17635 | 3878337 | 3878573 | + | 78 | - | hypothetical protein |
| 1059 | AA098_17640 | 3878605 | 3878976 | - | 123 | - | MerR family transcriptional regulator |
| 1060 | AA098_17645 | 3879049 | 3880146 | + | 365 | - | NAD-dependent dehydratase |
| 1061 | AA098_17650 | 3880273 | 3880572 | - | 99 | - | hypothetical protein |
| 1062 | AA098_17655 | 3880708 | 3880905 | - | 65 | - | hypothetical protein |
| 1063 | AA098_17660 | 3881335 | 3881529 | + | 64 | - | hypothetical protein |

| | | | | | | | |
|---|---|---|---|---|---|---|---|
| 1064 | AA098_17675 | 3884865 | 3885197 | + | 110 | - | hypothetical protein |
| 1065 | AA098_17725 | 3892152 | 3892331 | - | 59 | - | hypothetical protein |
| 1066 | AA098_17755 | 3898946 | 3899131 | + | 61 | - | hypothetical protein |
| 1067 | AA098_17760 | 3899303 | 3900595 | - | 430 | - | porin |
| 1068 | AA098_17765 | 3900733 | 3902046 | - | 437 | - | MFS transporter permease |
| 1069 | AA098_17770 | 3902255 | 3902896 | - | 213 | - | isochorismatase |
| 1070 | AA098_17775 | 3902912 | 3903664 | - | 250 | - | Asp/Glu racemase |
| 1071 | AA098_17780 | 3903678 | 3904484 | - | 268 | - | alpha/beta hydrolase |
| 1072 | AA098_17785 | 3904481 | 3905629 | - | 382 | - | 6-hydroxynicotinate 3-monooxygenase |
| 1073 | AA098_17790 | 3905951 | 3907003 | + | 350 | - | 2,5-dihydroxypyridine 5,6-dioxygenase |
| 1074 | AA098_17795 | 3907074 | 3907550 | + | 158 | - | MarR family transcriptional regulator |
| 1075 | AA098_17800 | 3908270 | 3908743 | + | 157 | - | (2Fe-2S)-binding protein |
| 1076 | AA098_17805 | 3908740 | 3912303 | + | 1187 | - | aldehyde dehydrogenase |
| 1077 | AA098_17810 | 3912307 | 3912945 | - | 212 | - | TetR family transcriptional regulator |
| 1078 | AA098_17815 | 3913232 | 3915397 | - | 721 | - | chemotaxis protein |
| 1079 | AA098_17820 | 3915789 | 3916484 | + | 231 | - | 3-oxoadipate CoA-transferase |
| 1080 | AA098_17825 | 3916493 | 3917134 | + | 213 | - | 3-oxoadipate CoA-transferase |
| 1081 | AA098_17840 | 3921093 | 3921449 | - | 118 | - | hypothetical protein |
| 1082 | AA098_17845 | 3921521 | 3922810 | - | 429 | - | ammonia channel protein |
| 1083 | AA098_17870 | 3927679 | 3928281 | + | 200 | - | XRE family transcriptional regulator |
| 1084 | AA098_17875 | 3928529 | 3929395 | + | 288 | - | formyltetrahydrofolate deformylase |
| 1085 | AA098_17880 | 3929392 | 3930297 | + | 301 | - | methenyltetrahydrofolate cyclohydrolase |
| 1086 | AA098_17885 | 3930279 | 3931520 | + | 413 | - | sarcosine oxidase subunit beta |
| 1087 | AA098_17890 | 3931531 | 3931830 | + | 99 | - | sarcosine oxidase subunit delta |
| 1088 | AA098_17900 | 3934726 | 3935307 | + | 193 | - | sarcosine oxidase |
| 1089 | AA098_17905 | 3935417 | 3936196 | + | 259 | - | IclR family transcriptional regulator |
| 1090 | AA098_17915 | 3936673 | 3937149 | - | 158 | - | AsnC family transcriptional regulator |
| 1091 | AA098_17920 | 3937287 | 3938153 | - | 288 | - | LysR family transcriptional regulator |

| | | | | | | | |
|---|---|---|---|---|---|---|---|
| 1092 | AA098_17925 | 3938273 | 3939187 | + | 304 | - | oxidoreductase |
| 1093 | AA098_17930 | 3939617 | 3940651 | + | 344 | - | acetylpolyamine aminohydrolase |
| 1094 | AA098_17935 | 3940699 | 3941637 | - | 312 | - | amidinotransferase |
| 1095 | AA098_17940 | 3941672 | 3942724 | - | 350 | - | ornithine cyclodeaminase |
| 1096 | AA098_17950 | 3943701 | 3945116 | + | 471 | - | amino acid transporter |
| 1097 | AA098_17955 | 3945127 | 3945363 | + | 78 | - | transcriptional regulator |
| 1098 | AA098_18040 | 3961450 | 3962808 | - | 452 | - | amidase |
| 1099 | AA098_18045 | 3963107 | 3964111 | + | 334 | - | GntR family transcriptional regulator |
| 1100 | AA098_18050 | 3964698 | 3965570 | + | 290 | - | xylose isomerase |
| 1101 | AA098_18055 | 3965655 | 3966845 | - | 396 | - | ABC transporter permease |
| 1102 | AA098_18060 | 3966842 | 3968377 | - | 511 | - | ABC transporter ATP-binding protein |
| 1103 | AA098_18065 | 3968433 | 3969500 | - | 355 | - | sugar ABC transporter substrate-binding protein |
| 1104 | AA098_18070 | 3969594 | 3970679 | - | 361 | - | oxidoreductase |
| 1105 | AA098_18075 | 3970731 | 3971726 | - | 331 | - | myo-inositol 2-dehydrogenase |
| 1106 | AA098_18080 | 3971770 | 3972636 | - | 288 | - | xylose isomerase |
| 1107 | AA098_18085 | 3972635 | 3972814 | + | 59 | - | hypothetical protein |
| 1108 | AA098_18090 | 3972955 | 3973869 | + | 304 | - | LysR family transcriptional regulator |
| 1109 | AA098_18095 | 3973974 | 3974357 | + | 127 | - | ketosteroid isomerase |
| 1110 | AA098_18100 | 3974427 | 3975497 | + | 356 | - | Maleylacetate reductase |
| 1111 | AA098_18105 | 3975556 | 3976470 | - | 304 | - | LysR family transcriptional regulator |
| 1112 | AA098_18110 | 3976627 | 3977499 | + | 290 | - | hydroxyquinol 1,2-dioxygenase |
| 1113 | AA098_18115 | 3977559 | 3977864 | + | 101 | - | stress responsive protein |
| 1114 | AA098_18120 | 3978529 | 3979887 | - | 452 | - | porin |
| 1115 | AA098_18125 | 3980112 | 3981569 | - | 485 | - | IolT |
| 1116 | AA098_18350 | 4031385 | 4032335 | + | 316 | - | transposase |
| 1117 | AA098_18400 | 4047146 | 4047412 | + | 88 | - | hypothetical protein |
| 1118 | AA098_18440 | 4062379 | 4063236 | + | 285 | - | hypothetical protein |
| 1119 | AA098_18490 | 4074391 | 4078017 | - | 1208 | - | type VI secretion protein VasK |

| | | | | | | | |
|---|---|---|---|---|---|---|---|
| 1120 | AA098_18495 | 4078014 | 4079480 | - | 488 | - | membrane protein |
| 1121 | AA098_18500 | 4079468 | 4079893 | - | 141 | - | hypothetical protein |
| 1122 | AA098_18505 | 4080375 | 4080878 | + | 167 | - | type VI secretion protein |
| 1123 | AA098_18510 | 4080896 | 4082383 | + | 495 | - | type VI secretion protein |
| 1124 | AA098_18515 | 4082380 | 4082790 | + | 136 | - | hypothetical protein |
| 1125 | AA098_18520 | 4082794 | 4084560 | + | 588 | - | type VI secretion protein |
| 1126 | AA098_18525 | 4084524 | 4085540 | + | 338 | - | type VI secretion protein |
| 1127 | AA098_18530 | 4085537 | 4085749 | + | 70 | - | hypothetical protein |
| 1128 | AA098_18535 | 4085746 | 4086543 | + | 265 | - | hypothetical protein |
| 1129 | AA098_18540 | 4086593 | 4087930 | + | 445 | - | type VI secretion protein |
| 1130 | AA098_18545 | 4087932 | 4088801 | + | 289 | - | type VI secretion system protein |
| 1131 | AA098_18550 | 4088935 | 4089450 | + | 171 | - | major exported protein |
| 1132 | AA098_18560 | 4091557 | 4091988 | + | 143 | - | hypothetical protein |
| 1133 | AA098_18570 | 4097438 | 4097863 | + | 141 | - | hypothetical protein |
| 1134 | AA098_18575 | 4097860 | 4098051 | + | 63 | - | hypothetical protein |
| 1135 | AA098_18580 | 4098376 | 4099053 | + | 225 | - | DSBA oxidoreductase |
| 1136 | AA098_18585 | 4099414 | 4100751 | - | 445 | - | porin |
| 1137 | AA098_18590 | 4101048 | 4101488 | + | 146 | - | hypothetical protein |
| 1138 | AA098_18595 | 4101493 | 4102848 | + | 451 | - | acetyl-CoA carboxylase |
| 1139 | AA098_18600 | 4102851 | 4103264 | + | 137 | - | hypothetical protein |
| 1140 | AA098_18605 | 4103269 | 4104036 | + | 255 | - | hypothetical protein |
| 1141 | AA098_18610 | 4104179 | 4104925 | + | 248 | - | oxidoreductase |
| 1142 | AA098_18615 | 4105021 | 4105722 | + | 233 | - | ABC transporter permease |
| 1143 | AA098_18620 | 4105729 | 4106487 | + | 252 | - | amino acid ABC transporter permease |
| 1144 | AA098_18630 | 4107284 | 4108117 | + | 277 | - | ABC transporter |
| 1145 | AA098_18635 | 4108277 | 4109479 | + | 400 | - | aspartate aminotransferase |
| 1146 | AA098_18640 | 4109543 | 4110514 | - | 323 | - | allophanate hydrolase |
| 1147 | AA098_18645 | 4110511 | 4111212 | - | 233 | - | allophanate hydrolase |

| | | | | | | | |
|---|---|---|---|---|---|---|---|
| 1148 | AA098_18650 | 4111241 | 4112752 | - | 503 | - | DSBA oxidoreductase |
| 1149 | AA098_18655 | 4113013 | 4113927 | + | 304 | - | nitrogen assimilation transcriptional regulator |
| 1150 | AA098_18660 | 4114035 | 4114259 | + | 74 | - | hypothetical protein |
| 1151 | AA098_18690 | 4119547 | 4119936 | - | 129 | - | Cro/Cl family transcriptional regulator |
| 1152 | AA098_18700 | 4120498 | 4120884 | - | 128 | - | MarR family transcriptional regulator |
| 1153 | AA098_18755 | 4133069 | 4134778 | - | 569 | - | peptidase S8 and S53 subtilisin kexin sedolisin |
| 1154 | AA098_18760 | 4134832 | 4136007 | - | 391 | - | hypothetical protein |
| 1155 | AA098_18850 | 4155537 | 4155914 | + | 125 | - | transcriptional regulator |
| 1156 | AA098_18940 | 4174923 | 4175390 | + | 155 | - | DNA-binding protein |
| 1157 | AA098_18955 | 4179108 | 4180343 | - | 411 | - | amine oxidase |
| 1158 | AA098_19015 | 4193235 | 4193609 | - | 124 | - | hypothetical protein |
| 1159 | AA098_19105 | 4212574 | 4214013 | + | 479 | - | RND transporter |
| 1160 | AA098_19110 | 4214090 | 4215109 | + | 339 | - | siderophore biosynthesis protein |
| 1161 | AA098_19120 | 4216988 | 4221196 | + | 1402 | - | peptide synthetase |
| 1162 | AA098_19125 | 4221282 | 4222112 | + | 276 | - | chromophore maturation protein PvdO |
| 1163 | AA098_19130 | 4222170 | 4224662 | - | 830 | - | ligand-gated channel protein |
| 1164 | AA098_19135 | 4224971 | 4228147 | - | 1058 | - | thioester reductase |
| 1165 | AA098_19140 | 4228144 | 4238442 | - | 3432 | - | peptide synthase |
| 1166 | AA098_19145 | 4238455 | 4241844 | - | 1129 | - | peptide synthetase |
| 1167 | AA098_19150 | 4241844 | 4257536 | - | 5230 | - | peptide synthetase |
| 1168 | AA098_19160 | 4258978 | 4259520 | + | 180 | - | thiamine pyrophosphate-binding protein |
| 1169 | AA098_19235 | 4276471 | 4276905 | - | 144 | - | hypothetical protein |
| 1170 | AA098_19275 | 4284635 | 4285036 | - | 133 | - | antitoxin |
| 1171 | AA098_19280 | 4285039 | 4285335 | - | 98 | - | mRNA interferase MqsR |
| 1172 | AA098_19310 | 4291245 | 4292534 | + | 429 | - | class V aminotransferase |
| 1173 | AA098_19315 | 4292599 | 4293720 | + | 373 | - | cupin |
| 1174 | AA098_19340 | 4298828 | 4299316 | + | 162 | - | hypothetical protein |
| 1175 | AA098_19345 | 4299335 | 4299952 | + | 205 | - | glutamyl-tRNA amidotransferase |

| | | | | | | | |
|---|---|---|---|---|---|---|---|
| 1176 | AA098_19395 | 4311528 | 4312265 | + | 245 | - | hypothetical protein |
| 1177 | AA098_19400 | 4312265 | 4312966 | + | 233 | - | ABC transporter |
| 1178 | AA098_19575 | 4359710 | 4359943 | - | 77 | - | hypothetical protein |
| 1179 | AA098_19590 | 4362791 | 4363171 | - | 126 | - | hypothetical protein |
| 1180 | AA098_19595 | 4363258 | 4364769 | - | 503 | - | MFS transporter |
| 1181 | AA098_19600 | 4364965 | 4365231 | - | 88 | - | methyltransferase |
| 1182 | AA098_19605 | 4365194 | 4365871 | + | 225 | - | mechanosensitive ion channel protein MscS |
| 1183 | AA098_19610 | 4365850 | 4366002 | + | 50 | - | mechanosensitive ion channel protein MscS |
| 1184 | AA098_19625 | 4368076 | 4368513 | + | 145 | - | AsnC family transcriptional regulator |
| 1185 | AA098_19670 | 4376367 | 4376558 | - | 63 | - | hypothetical protein |
| 1186 | AA098_19675 | 4376712 | 4377890 | - | 392 | - | integrase |
| 1187 | AA098_19735 | 4387636 | 4387974 | + | 112 | - | flagellar biosynthesis protein FlhB |
| 1188 | AA098_19740 | 4388042 | 4390753 | + | 903 | - | mannosyltransferase |
| 1189 | AA098_19915 | 4424357 | 4424878 | + | 173 | - | transposase |
| 1190 | AA098_19920 | 4424908 | 4425711 | + | 267 | - | integrase |
| 1191 | AA098_19930 | 4427440 | 4427733 | - | 97 | - | flagellar assembly protein FliT |
| 1192 | AA098_19950 | 4430087 | 4431835 | - | 582 | - | flagellin |
| 1193 | AA098_19995 | 4442048 | 4442239 | - | 63 | - | hypothetical protein |
| 1194 | AA098_20055 | 4451728 | 4453050 | - | 440 | - | glutamate--ammonia ligase |
| 1195 | AA098_20095 | 4460656 | 4461834 | + | 392 | - | integrase |
| 1196 | AA098_20105 | 4462490 | 4463872 | + | 460 | - | GABA permease |
| 1197 | AA098_20115 | 4464143 | 4464736 | - | 197 | - | ribose-phosphate pyrophosphokinase |
| 1198 | AA098_20255 | 4491977 | 4496176 | + | 1399 | - | hypothetical protein |
| 1199 | AA098_20460 | 4539927 | 4540199 | + | 90 | - | hypothetical protein |
| 1200 | AA098_20585 | 4570064 | 4570468 | + | 134 | - | acetyltransferase |
| 1201 | AA098_20600 | 4572076 | 4572255 | - | 59 | - | hypothetical protein |
| 1202 | AA098_20855 | 4624447 | 4625064 | + | 205 | - | amino acid transporter LysE |
| 1203 | AA098_20860 | 4625165 | 4626373 | - | 402 | - | hypothetical protein |

| | | | | | | | |
|---|---|---|---|---|---|---|---|
| 1204 | AA098_20865 | 4626384 | 4628294 | - | 636 | - | hypothetical protein |
| 1205 | AA098_20870 | 4628303 | 4629163 | - | 286 | - | hypothetical protein |
| 1206 | AA098_20875 | 4629806 | 4630069 | + | 87 | - | hypothetical protein |
| 1207 | AA098_20880 | 4630206 | 4630478 | + | 90 | - | hypothetical protein |
| 1208 | AA098_20890 | 4631405 | 4631797 | + | 130 | - | hypothetical protein |
| 1209 | AA098_20945 | 4640047 | 4640850 | - | 267 | - | integrase |
| 1210 | AA098_20950 | 4640880 | 4641401 | - | 173 | - | transposase |
| 1211 | AA098_21075 | 4664350 | 4664556 | - | 68 | - | hypothetical protein |
| 1212 | AA098_21145 | 4680191 | 4681873 | - | 560 | - | hypothetical protein |
| 1213 | AA098_21150 | 4681870 | 4682715 | - | 281 | - | hypothetical protein |
| 1214 | AA098_21230 | 4700770 | 4701048 | - | 92 | - | hypothetical protein |
| 1215 | AA098_21235 | 4701192 | 4701665 | + | 157 | - | hypothetical protein |
| 1216 | AA098_21240 | 4702366 | 4703625 | + | 419 | - | hypothetical protein |
| 1217 | AA098_21245 | 4703609 | 4705396 | + | 595 | - | thiamine biosynthesis protein ThiF |
| 1218 | AA098_21410 | 4746489 | 4747994 | + | 501 | - | DNA polymerase |
| 1219 | AA098_21425 | 4752571 | 4753599 | + | 342 | - | transcriptional regulator |
| 1220 | AA098_21430 | 4754041 | 4755069 | + | 342 | - | transcriptional regulator |
| 1221 | AA098_21465 | 4764921 | 4765949 | - | 342 | - | transcriptional regulator |
| 1222 | AA098_21495 | 4774296 | 4775171 | - | 291 | - | hypothetical protein |
| 1223 | AA098_21500 | 4775285 | 4778098 | - | 937 | - | phosphoesterase |
| 1224 | AA098_21505 | 4778652 | 4780301 | + | 549 | - | hypothetical protein |
| 1225 | AA098_21510 | 4780450 | 4781868 | - | 472 | - | hypothetical protein |
| 1226 | AA098_21515 | 4782031 | 4783017 | - | 328 | - | ATP-binding protein |
| 1227 | AA098_21520 | 4783430 | 4784338 | - | 302 | - | recombinase |
| 1228 | AA098_21525 | 4784512 | 4785489 | + | 325 | - | TnpT protein |
| 1229 | AA098_21530 | 4785825 | 4786253 | + | 142 | - | hypothetical protein |
| 1230 | AA098_21535 | 4786638 | 4786901 | - | 87 | - | hypothetical protein |
| 1231 | AA098_21540 | 4787966 | 4788844 | - | 292 | - | hypothetical protein |

| | | | | | | | |
|---|---|---|---|---|---|---|---|
| 1232 | AA098_21545 | 4789522 | 4789893 | + | 123 | - | hypothetical protein |
| 1233 | AA098_21555 | 4791978 | 4792766 | + | 262 | - | hypothetical protein |
| 1234 | AA098_21560 | 4794053 | 4795267 | + | 404 | - | hypothetical protein |
| 1235 | AA098_21565 | 4796355 | 4796594 | - | 79 | - | bifunctional antitoxin/transcriptional repressor RelB |
| 1236 | AA098_21575 | 4799302 | 4800108 | + | 268 | - | NAD-dependent dehydratase |
| 1237 | AA098_21580 | 4800131 | 4801012 | + | 293 | - | gluconolactonase |
| 1238 | AA098_21585 | 4801077 | 4802048 | + | 323 | - | C4-dicarboxylate ABC transporter |
| 1239 | AA098_21590 | 4802115 | 4802642 | + | 175 | - | C4-dicarboxylate ABC transporter |
| 1240 | AA098_21595 | 4802643 | 4803923 | + | 426 | - | membrane protein |
| 1241 | AA098_21600 | 4803978 | 4804901 | + | 307 | - | membrane protein |
| 1242 | AA098_21605 | 4804926 | 4805795 | + | 289 | - | aldose epimerase |
| 1243 | AA098_21630 | 4810380 | 4811369 | - | 329 | - | asparaginase |
| 1244 | AA098_21675 | 4821707 | 4822603 | + | 298 | - | membrane protein |
| 1245 | AA098_21860 | 4861068 | 4861970 | - | 300 | - | pyridoxal-5'-phosphate-dependent protein |
| 1246 | AA098_21870 | 4863175 | 4863963 | - | 262 | - | synthetase |
| 1247 | AA098_21875 | 4863960 | 4864664 | - | 234 | - | serine acetyltransferase |
| 1248 | AA098_21910 | 4875273 | 4875923 | + | 216 | - | DEAD/DEAH box helicase |
| 1249 | AA098_21950 | 4882519 | 4883121 | + | 200 | - | electron transporter RnfG |
| 1250 | AA098_21965 | 4884386 | 4884562 | + | 58 | - | leucyl-tRNA synthetase |
| 1251 | AA098_22065 | 4903798 | 4908120 | - | 1440 | - | hypothetical protein |
| 1252 | AA098_22115 | 4917437 | 4917850 | - | 137 | - | DNA mismatch repair protein MutT |
| 1253 | AA098_22155 | 4931620 | 4932006 | - | 128 | - | type II secretion pathway protein XcpZ |
| 1254 | AA098_22160 | 4932003 | 4933091 | - | 362 | - | general secretion pathway protein GspL |
| 1255 | AA098_22175 | 4934028 | 4934459 | - | 143 | - | general secretion pathway protein GspH |
| 1256 | AA098_22260 | 4956228 | 4959185 | + | 985 | - | hypothetical protein |
| 1257 | AA098_22280 | 4963058 | 4963813 | - | 251 | - | permease |
| 1258 | AA098_22385 | 4988387 | 4989337 | - | 316 | - | transposase |
| 1259 | AA098_22440 | 5002173 | 5002541 | + | 122 | - | acetyl-CoA carboxylase |

| | | | | | | | |
|---|---|---|---|---|---|---|---|
| 1260 | AA098_22455 | 5005597 | 5006238 | - | 213 | - | hypothetical protein |
| 1261 | AA098_22460 | 5006228 | 5006812 | - | 194 | - | RNA polymerase sigma factor |
| 1262 | AA098_22465 | 5007037 | 5007324 | + | 95 | - | membrane protein |
| 1263 | AA098_22470 | 5007341 | 5008174 | + | 277 | - | hypothetical protein |
| 1264 | AA098_22475 | 5008171 | 5008944 | + | 257 | - | hypothetical protein |
| 1265 | AA098_22480 | 5008937 | 5009404 | + | 155 | - | DoxX family protein |
| 1266 | AA098_22565 | 5027907 | 5028383 | - | 158 | - | ketosteroid isomerase |
| 1267 | AA098_22575 | 5029787 | 5030437 | - | 216 | - | GntR family transcriptional regulator |
| 1268 | AA098_22580 | 5030577 | 5031761 | + | 394 | - | hypothetical protein |
| 1269 | AA098_22685 | 5047788 | 5048681 | - | 297 | - | divalent cation transporter |
| 1270 | AA098_22695 | 5049306 | 5049767 | - | 153 | - | hypothetical protein |
| 1271 | AA098_22795 | 5072671 | 5073558 | - | 295 | - | acyl-CoA dehydrogenase |
| 1272 | AA098_22875 | 5090669 | 5091151 | + | 160 | - | hypothetical protein |
| 1273 | AA098_22880 | 5091166 | 5091552 | + | 128 | - | hypothetical protein |
| 1274 | AA098_22940 | 5104471 | 5105391 | - | 306 | - | hypothetical protein |
| 1275 | AA098_22945 | 5105570 | 5106817 | + | 415 | - | hypothetical protein |
| 1276 | AA098_22970 | 5110811 | 5111389 | - | 192 | - | glutamine amidotransferase |
| 1277 | AA098_22980 | 5112600 | 5113181 | - | 193 | - | hypothetical protein |
| 1278 | AA098_22985 | 5113226 | 5113522 | - | 98 | - | amidase |
| 1279 | AA098_23055 | 5133128 | 5133691 | + | 187 | - | hypothetical protein |
| 1280 | AA098_23295 | 5183350 | 5183997 | + | 215 | - | hypothetical protein |
| 1281 | AA098_23305 | 5184658 | 5184954 | - | 98 | - | addiction module antitoxin |
| 1282 | AA098_23310 | 5184951 | 5185244 | - | 97 | - | addiction module antitoxin RelB |
| 1283 | AA098_23330 | 5187983 | 5188792 | - | 269 | - | phosphonate ABC transporter |
| 1284 | AA098_23350 | 5192052 | 5192438 | - | 128 | - | hypothetical protein |
| 1285 | AA098_23355 | 5192459 | 5192998 | - | 179 | - | hypothetical protein |
| 1286 | AA098_23370 | 5194459 | 5195586 | - | 375 | - | hypothetical protein |
| 1287 | AA098_23420 | 5206640 | 5230720 | + | 8026 | - | adhesin |

| | | | | | | | |
|---|---|---|---|---|---|---|---|
| 1288 | AA098_23425 | 5230784 | 5232220 | + | 478 | - | channel protein TolC |
| 1289 | AA098_23430 | 5232217 | 5234385 | + | 722 | - | ATP-binding protein |
| 1290 | AA098_23435 | 5234382 | 5235566 | + | 394 | - | secretion protein HlyD |
| 1291 | AA098_23450 | 5237067 | 5237801 | + | 244 | - | Fe-S oxidoreductase |
| 1292 | AA098_23475 | 5242130 | 5242351 | + | 73 | - | hypothetical protein |
| 1293 | AA098_23585 | 5266868 | 5267092 | + | 74 | - | hypothetical protein |
| 1294 | AA098_23605 | 5272366 | 5272665 | + | 99 | - | hypothetical protein |
| 1295 | AA098_23655 | 5283099 | 5283284 | - | 61 | - | LITAF-like zinc ribbon domain containing protein |
| 1296 | AA098_23670 | 5285121 | 5285327 | + | 68 | - | hypothetical protein |
| 1297 | AA098_23740 | 5296826 | 5297725 | + | 299 | - | MerR family transcriptional regulator |
| 1298 | AA098_23750 | 5299155 | 5299853 | + | 232 | - | short-chain dehydrogenase |
| 1299 | AA098_23825 | 5313911 | 5314633 | - | 240 | - | short-chain dehydrogenase |
| 1300 | AA098_23830 | 5314649 | 5315497 | - | 282 | - | thiamine biosynthesis protein ThiJ |
| 1301 | AA098_23835 | 5315614 | 5316537 | + | 307 | - | LuxR family transcriptional regulator |
| 1302 | AA098_23870 | 5324303 | 5325118 | - | 271 | - | polyphosphate kinase |
| 1303 | AA098_23915 | 5335666 | 5336181 | + | 171 | - | RNA polymerase sigma factor |
| 1304 | AA098_23920 | 5336168 | 5337124 | + | 318 | - | iron dicitrate transport regulator FecR |
| 1305 | AA098_23935 | 5340022 | 5340945 | + | 307 | - | peptide ABC transporter substrate-binding protein |
| 1306 | AA098_23955 | 5342947 | 5343522 | - | 191 | - | peptidase M15 |
| 1307 | AA098_24120 | 5379586 | 5380971 | + | 461 | - | gluconate transporter |
| 1308 | AA098_24125 | 5381068 | 5381766 | - | 232 | - | hypothetical protein |
| 1309 | AA098_24135 | 5382812 | 5383051 | - | 79 | - | hypothetical protein |
| 1310 | AA098_24140 | 5383623 | 5384444 | + | 273 | - | hypothetical protein |
| 1311 | AA098_24145 | 5385792 | 5386229 | - | 145 | - | DNA-binding protein |
| 1312 | AA098_24160 | 5390212 | 5391240 | + | 342 | - | transcriptional regulator |
| 1313 | AA098_24170 | 5394623 | 5394913 | - | 96 | - | hypothetical protein |
| 1314 | AA098_24280 | 5409756 | 5410520 | + | 254 | - | GntR family transcriptional regulator |
| 1315 | AA098_24285 | 5410611 | 5411813 | + | 400 | - | branched-chain amino acid ABC transporter substrate-binding protein |

| | | | | | | | |
|---|---|---|---|---|---|---|---|
| 1316 | AA098_24290 | 5411879 | 5412739 | + | 286 | - | ABC transporter permease |
| 1317 | AA098_24295 | 5412741 | 5413760 | + | 339 | - | ABC transporter permease |
| 1318 | AA098_24300 | 5413760 | 5414512 | + | 250 | - | ABC transporter |
| 1319 | AA098_24305 | 5414512 | 5415207 | + | 231 | - | ABC transporter |
| 1320 | AA098_24310 | 5415321 | 5416598 | + | 425 | - | allantoate amidohydrolase |
| 1321 | AA098_24315 | 5416600 | 5418009 | + | 469 | - | amidase |
| 1322 | AA098_24330 | 5419911 | 5420375 | - | 154 | - | hypothetical protein |
| 1323 | AA098_24335 | 5420372 | 5421010 | - | 212 | - | pilus assembly protein PilW |
| 1324 | AA098_24340 | 5421007 | 5421189 | - | 60 | - | pilus assembly protein PilV |
| 1325 | AA098_24345 | 5421299 | 5421781 | + | 160 | - | type IV pili biogenesis protein FimT |
| 1326 | AA098_24510 | 5459336 | 5460187 | + | 283 | - | acyl dehydratase |
| 1327 | AA098_24540 | 5463460 | 5464236 | + | 258 | - | LuxR family transcriptional regulator |
| 1328 | AA098_24570 | 5475942 | 5476373 | + | 143 | - | hypothetical protein |
| 1329 | AA098_24580 | 5478473 | 5478844 | - | 123 | - | translation initiation factor Sui1 |
| 1330 | AA098_24745 | 5517513 | 5518286 | + | 257 | - | cystathionine gamma-lyase |
| 1331 | AA098_24750 | 5518779 | 5519243 | + | 154 | - | cation-binding protein |
| 1332 | AA098_24755 | 5519420 | 5521609 | - | 729 | - | TonB-dependent receptor |
| 1333 | AA098_24760 | 5521791 | 5522483 | + | 230 | - | transcriptional regulator |
| 1334 | AA098_24765 | 5522483 | 5523811 | + | 442 | - | histidine kinase |
| 1335 | AA098_24770 | 5523912 | 5524805 | + | 297 | - | esterase |
| 1336 | AA098_24775 | 5524827 | 5525807 | - | 326 | - | zeta toxin family protein |
| 1337 | AA098_24800 | 5530819 | 5532660 | + | 613 | - | TonB-dependent receptor |
| 1338 | AA098_24830 | 5535925 | 5536893 | - | 322 | - | thiamine monophosphate kinase |
| 1339 | AA098_24910 | 5548195 | 5549400 | - | 401 | - | major facilitator transporter |
| 1340 | AA098_24935 | 5552998 | 5553843 | + | 281 | - | hypothetical protein |
| 1341 | AA098_25320 | 5621909 | 5622397 | - | 162 | - | hypothetical protein |
| 1342 | AA098_25325 | 5622730 | 5623128 | - | 132 | - | transposase |
| 1343 | AA098_25395 | 5634116 | 5634448 | + | 110 | - | membrane protein |

| | | | | | | | |
|---|---|---|---|---|---|---|---|
| 1344 | AA098_25405 | 5635627 | 5636031 | - | 134 | - | plasmid maintenance protein |
| 1345 | AA098_25410 | 5636031 | 5636261 | - | 76 | - | antitoxin |
| 1346 | AA098_25425 | 5638438 | 5638935 | + | 165 | - | DNA repair protein RadC |
| 1347 | AA098_25430 | 5639405 | 5639983 | - | 192 | - | hypothetical protein |
| 1348 | AA098_25435 | 5639987 | 5641495 | - | 502 | - | transposase |
| 1349 | AA098_25440 | 5641531 | 5641875 | - | 114 | - | isocitrate lyase |
| 1350 | AA098_25445 | 5641872 | 5642198 | - | 108 | - | transposase |
| 1351 | AA098_25450 | 5642328 | 5642516 | - | 62 | - | hypothetical protein |
| 1352 | AA098_25460 | 5643835 | 5645901 | - | 688 | - | DEAD/DEAH box helicase |
| 1353 | AA098_25465 | 5645960 | 5648725 | - | 921 | - | lactate dehydrogenase |
| 1354 | AA098_25475 | 5649726 | 5650379 | - | 217 | - | transposase |
| 1355 | AA098_25480 | 5650683 | 5650883 | - | 66 | - | transcriptional regulator |
| 1356 | AA098_25485 | 5651466 | 5652179 | + | 237 | - | hypothetical protein |
| 1357 | AA098_25490 | 5652263 | 5653462 | - | 399 | - | integrase |
| 1358 | AA098_25595 | 5678219 | 5678551 | + | 110 | - | sulfurtransferase |
| 1359 | AA098_25665 | 5696792 | 5697346 | - | 184 | - | serine acetyltransferase |
| 1360 | AA098_25685 | 5700940 | 5703240 | + | 766 | - | peptidase M16 |
| 1361 | AA098_25750 | 5717811 | 5718491 | - | 226 | - | dethiobiotin synthetase |
| 1362 | AA098_25780 | 5723140 | 5723904 | + | 254 | - | ModE family transcriptional regulator |
| 1363 | AA098_25840 | 5738416 | 5738601 | + | 61 | - | hypothetical protein |
| 1364 | AA098_25945 | 5767341 | 5768033 | + | 230 | - | YD repeat protein |
| 1365 | AA098_25995 | 5781039 | 5781341 | - | 100 | - | ZapA |
| 1366 | AA098_26050 | 5795837 | 5796253 | + | 138 | - | hypothetical protein |
| 1367 | AA098_26055 | 5796392 | 5797084 | + | 230 | - | hypothetical protein |
| 1368 | AA098_26060 | 5797156 | 5797773 | + | 205 | - | hypothetical protein |
| 1369 | AA098_26065 | 5797777 | 5798724 | - | 315 | - | AraC family transcriptional regulator |
| 1370 | AA098_26085 | 5801815 | 5802285 | + | 156 | - | 4-hydroxybenzoyl-CoA thioesterase |
| 1371 | AA098_26090 | 5802554 | 5803582 | + | 342 | - | transcriptional regulator |

| | | | | | | | |
|---|---|---|---|---|---|---|---|
| 1372 | AA098_26095 | 5803571 | 5805253 | - | 560 | - | ricin B lectin |
| 1373 | AA098_26180 | 5821728 | 5821955 | - | 75 | - | hypothetical protein |
| 1374 | AA098_26235 | 5830924 | 5832243 | + | 439 | - | integrase |
| 1375 | AA098_26240 | 5832620 | 5832889 | + | 89 | - | hypothetical protein |
| 1376 | AA098_26245 | 5833173 | 5834153 | + | 326 | - | integrase |
| 1377 | AA098_26250 | 5834639 | 5835985 | + | 448 | - | reverse transcriptase |
| 1378 | AA098_26255 | 5835978 | 5838026 | + | 682 | - | reverse transcriptase |
| 1379 | AA098_26260 | 5838527 | 5840137 | - | 536 | - | diguanylate cyclase |
| 1380 | AA098_26265 | 5840318 | 5840524 | + | 68 | - | hypothetical protein |
| 1381 | AA098_26575 | 5910181 | 5911911 | + | 576 | - | dipeptidase |
| 1382 | AA098_26615 | 5917076 | 5917663 | - | 195 | - | membrane protein |
| 1383 | AA098_26625 | 5919624 | 5920103 | + | 159 | - | hypothetical protein |
| 1384 | AA098_26630 | 5920155 | 5920733 | + | 192 | - | hypothetical protein |
| 1385 | AA098_26690 | 5930895 | 5931197 | + | 100 | - | hypothetical protein |
| 1386 | AA098_26900 | 6007029 | 6007346 | + | 105 | - | holliday junction resolvasome, helicase subunit |
| 1387 | AA098_26980 | 6024524 | 6024994 | - | 156 | - | hypothetical protein |
| 1388 | AA098_27180 | 6064780 | 6066402 | + | 540 | - | hypothetical protein |
| 1389 | AA098_27190 | 6069899 | 6070579 | - | 226 | - | hypothetical protein |
| 1390 | AA098_27330 | 6095262 | 6095462 | - | 66 | - | hypothetical protein |
| 1391 | AA098_27435 | 6117355 | 6117762 | + | 135 | - | integral membrane-like protein |
| 1392 | AA098_27440 | 6118451 | 6118900 | + | 149 | - | hypothetical protein |
| 1393 | AA098_27445 | 6120569 | 6121096 | + | 175 | - | hypothetical protein |
| 1394 | AA098_27450 | 6121198 | 6121521 | - | 107 | - | hypothetical protein |
| 1395 | AA098_27455 | 6121639 | 6122313 | - | 224 | - | transcriptional regulator |
| 1396 | AA098_27480 | 6130541 | 6131569 | + | 342 | - | transcriptional regulator |
| 1397 | AA098_27485 | 6131606 | 6131929 | + | 107 | - | hypothetical protein |
| 1398 | AA098_27490 | 6132132 | 6134129 | + | 665 | - | ATPase |
| 1399 | AA098_27495 | 6134222 | 6134446 | - | 74 | - | hypothetical protein |

| | | | | | | | |
|---|---|---|---|---|---|---|---|
| 1400 | AA098_27500 | 6134574 | 6135599 | - | 341 | - | hypothetical protein |
| 1401 | AA098_27505 | 6135898 | 6137205 | - | 435 | - | porin |
| 1402 | AA098_27510 | 6137416 | 6138384 | - | 322 | - | LysR family transcriptional regulator |
| 1403 | AA098_27515 | 6138642 | 6139061 | + | 139 | - | sugar translocase |
| 1404 | AA098_27520 | 6139027 | 6140010 | + | 327 | - | ribonuclease III |
| 1405 | AA098_27525 | 6140004 | 6141458 | + | 484 | - | sugar transferase |
| 1406 | AA098_27535 | 6142029 | 6142616 | + | 195 | - | hypothetical protein |
| 1407 | AA098_27555 | 6146625 | 6146951 | + | 108 | - | hypothetical protein |
| 1408 | AA098_27560 | 6146984 | 6147334 | - | 116 | - | hypothetical protein |
| 1409 | AA098_27570 | 6148550 | 6148756 | - | 68 | - | hypothetical protein |
| 1410 | AA098_27580 | 6151373 | 6151579 | - | 68 | - | hypothetical protein |
| 1411 | AA098_27585 | 6151594 | 6151803 | - | 69 | - | hypothetical protein |
| 1412 | AA098_27590 | 6152024 | 6152947 | - | 307 | - | hypothetical protein |
| 1413 | AA098_27595 | 6153258 | 6153506 | - | 82 | - | hypothetical protein |
| 1414 | AA098_27600 | 6154130 | 6154801 | - | 223 | - | hypothetical protein |
| 1415 | AA098_27605 | 6154810 | 6155652 | - | 280 | - | hypothetical protein |
| 1416 | AA098_27610 | 6156298 | 6156657 | - | 119 | - | transcriptional regulator |
| 1417 | AA098_27615 | 6156714 | 6158630 | - | 638 | - | hypothetical protein |
| 1418 | AA098_27620 | 6158706 | 6159665 | - | 319 | - | ATPase AAA |
| 1419 | AA098_27625 | 6159662 | 6161620 | - | 652 | - | integrase |
| 1420 | AA098_27630 | 6161601 | 6162239 | - | 212 | - | TnsA endonuclease |
