# Supplementary material for: Comparative Genomics Reveals Specific Genetic Architectures in Nicotine Metabolism of Pseudomonas sp. JY-Q
Source: Front Microbiol. 2017 Oct 31;8:2085. doi: 10.3389/fmicb.2017.02085 (PMC5674928; doi:10.3389/fmicb.2017.02085)
Supplement: Supplementary file 2 [file DataSheet2.pdf]

Supplementary file

Supplementary Table S1. *Pseudomonas* sp. JY-Q non-conserved protein-coding genes against strain S16. (See Appendix)

Supplementary Table S2. JY-Q potential secondary metabolite, virulence and antibiotic-resistance features.

NO.	Type	Coordinate start (bp)	Coordinate end (bp)	Size (kb)	Putative function
1	Chemotaxis	457745	467359	9.5	Bacterial motility
2	Virulence	1121332	1137453	16.2	Alginate biosynthesis
3	T1PKS	1666998	1714536	47.5	O-antigen biosynthesis
4	Bacteriocin	2368843	2379676	10.8	-
5	NRPS	4196988	4277536	80.5	Pyoverdine biosynthesis
6	NRPS	4293009	4345962	52.9	Pyoverdine biosynthesis
7	Flagellum	4425794	4445231	19.4	Falgellar assembly
8	Bacteriocin	5002341	5013174	10.8	-

Supplementary Table S3. Insertion sequence types, genome coordinates and sequences of target site duplication if applicable.

IS type	Genome coordinate		Orientation[a]	Locus tag of transposase gene(s)	Sequence of Target Site Duplication	
	Start	End			Flanking region left	Flanking region right
IS*Pa41*	772529	773696	+	AA098_03465	GCGCAGTTATTGATGGAGCGCACGGTCGATAAGGGCCAGATCGTCGATCTC**AAAG**	**AAAG**AGCAGGTCAAGCTACTGCGCGACCGTCTGTTCAGTCGCAAGTCCGAGCAGA
	4724138	4725305	+	AA098_21345	AATCGCAGTGACGCCGGTCACACGACGGTCCCGGATGTAGCAAATAATCAC**CTAG**	**CTAG**GGGGTGCTGGCCACCCACTTATACCTGCAAGATTTTGAAGAACGTCTAATG
	4727315	4728482	-	AA098_21355	AGGTCCGAGTTCGCAATCAGCATCTCTTTAATCAAGTTGGAAAGATGCTCC**CTAG**	**CTAG**ATAAGGTTGCAAGAGGATCACCTGAGGGATTTTTCTTTCCTAGCAAAGAGT
	4733223	4732056	-	AA098_21370	ATGCTGGAGAGGGGCCAGTCGGCCTGCAGGCCGGTATCTGGGAGTGATCGC**CTAA**	**CTAA**GCGCTGGCTTTGCAAGGTGCTGGCGCGTGAGTTTAGTCATTTTATTGCCAG
	6146035	6144868	-	AA098_27550	TCTTCGCGTTAAGATTCAAGCGTCCTCAGGTGACCCGGCGTATGGACAGAG**GTAG**	**GTAG**CGACTACCACATGCCATACCCTCAGCCAAACTTTCGAACAGCCTGCAACCA
	1804854	1803687	-	AA098_08100	ACCAAATTACTTGAAAAACCCTCCGGCAAAAAATAGGGCAAGCTGCCTTCC**CCAG**	**CCAG**AGGGGATGTCACCAGATCTGTCATCCAAACCAATGAGCAGCAGCACCTCAC
	3242714	3241547	-	AA098_14740	CCGTCATCATTGAGACGCGCTGCCAACCATGCATCAATTCTGGGTGCAGCT**TTAG**	**TTAG**GTATGGTTTTAAGAGAAATTTCGGGGATTTCGCTGCATGCGGAGATGAGGC
	738976	737809	-	AA098_03275	ATCTCAGTGCTCTGTGATTTCCCTTTATTTCGCATGACCCGTTAGTCACGCTTTA	CTTATGGCATAATGACATTATTGCTCATGGAGACTCATCTTGCGCTCGAACCTGC
	4745298	4745885	+	AA098_21405	NA[b]	NA
	4748068	4748647	+	AA098_21415	NA	NA
IS*Psp7*	92055	93167	+	AA098_00410	AGAGGCTTCACTATGCGCAAACCAGAACTCGCCGCCGTCATCGCCGATAAGG**CTG**	**CTG**ATCTGACCAAGGAAAAGGCCAATCAGGTTTTGAACGCGATTCTCGACAGCAT
	737382	736270	-	AA098_03265	CGAGTCCGTTAGAGTCAAGCCGACTCGGGAGCAAATTGATCGCGCACAAAAG**CTG**	**CTG**TATGACCGGTTGCGTGCAGGTAAAAATTCCATTCCCCAAAATCGTCATTATG
	887223	888335	+	AA098_03915	GTTGCGGCCTCGGTTGTGCGGGGGATGTTGATGCCTTGTTAACGCTAAATAACAT	CTCCCTATTTTTATTCAGTGGTAGTACGGCACACGTAGTGCGCCGGAGACATCCG
	911808	912920	+	AA098_04030	GGTTGTTCAGTGGTCCGGTGTTTCTAAACGCAACTTTTTTGTTATGTCTATG**CAG**	**CAG**TATTAAACGCCAGCACGAACGTGGTTCTGTATATCGAGTTACTAAACTTGCT
	929439	928327	-	AA098_04115	AACAGCAAGGTACCAACGCATCCCTTCACAGAAAAATGCACCGACATGAAAACTC	CATCAATAGTGATCGAGCGTCTCTAACCTCAAAGCGCTTACCTAGCGAGGCCTGT
	1623289	1622177	-	AA098_07340	GTGAATTTACTTCTCTCAGATTCCGAGAGGCTTTCAGAGATTTTGCTTATAG**ATT**	**ATT**TCTTAAAAGCCTCGTCGGAGCTACCGTCGATCTTCGGTTCGCCACACCCCGA
	1634814	1633702	-	AA098_07385	ATAGATCGCTCACAACAGCGCCAGCCCAAGCTGGAGATTCTTCCCAATAAAT**CGT**	**CGT**CATAAGGAATTCCTAGCATTATCTAATGTGGCAAATGAGCGTTGCAGCTCCA
	1807182	1806070	-	AA098_08110	ATGCGAATCGCTTGGTCCAGCTCCTCATTGCTATCATCCGGGAACTCTATTG**GGG**	**GGG**CGTAATAGACGCGTGCATCCTGAACGTCGGCATTTTCATCCAGCTCCAACCT
	1809945	1811057	+	AA098_08130	GCAACCTGTTCGGGTCGAGCATGAAGGCGCGGAGCATGGGGGTGATACGTTC**AGC**	**AGC**TATAAAACCCTAACGGTAGACTACTGGTATTTGAAACATCTCACGAGGGAGC
	2942519	2943631	+	AA098_13395	CGTGGTTGGGCAGGCTTTTCGCCGTACGGGCGCGCTGATGTTCACCGTTACA**GGG**	**GGG**AGTAAATTATGCTAACTGACGTTCTTTCCATGCTCGCGCCGCAAAACAGGCG
	3683652	3682540	–	AA098_16705	TATCTATTTGTAGGAAAGTTCTTTGCGTCATATAGGCCATTTGTACTATTCA**GAG**	**GAG**AGTAAATTCAGGATGATTAACCGCTGTGCATATGGCGCTGGTCTCTCGTTGC
	4752516	4753628	+	AA098_21425	CGCTGTTGCCACTCCGACCGCGGCGCTACCAAGGAACTTTCGGCGTGATACT**CCG**	**CCG**CTTTTGCTGCCTTCATCACCATCTTTTGTCATGTTGTGGAAACCCCGATAGA
	4753986	4755098	+	AA098_21430	CCGTGAACACCAGTAGTGTCATTTGAGTGGCACACACTGCAAATTTGGTAAA**CCT**	**CCT**TTTTCCCATTTTCCGGATCACAGTTATCTGCACCTACAGCTACACCAGATAA
	4766004	4764892	–	AA098_21465	GAGCTTGGTTACTCTCAACTAAGCGCATTCAGTCGGGCGTTCCGTCGCTGGA**CTG**	**CTG**GATTAACGCCGATGGAGTACCGAAATACTTATAATAAACGCATGCTTTGATA
	5390157	5391269	+	AA098_24160	CTGGAAAATTTGTATCGCCCAATTTGGCACCCACGCACAATTTGGGGAATAC**AGC**	**AGC**CATAAGCCACGGTGGCGTCCAGACGGGTACGGATTTGGCGAACCTTTGACTA
	5802499	5803611	+	AA098_26090	GCCGCGCGTTATGGTCGTTATTTGTGAAACACCACACTAGGCCTCGGTTATT**GTG**	**GTG**GGATATCGAGATTTATCCAACTTTGGTACCTACCGTTATCACATGCCAGTAA
	6130486	6131598	+	AA098_27480	GGTGATGCCTATGCATTGCTAAGGTTACGCCCGAGAAGTTTTGAAATTGAGA**GGG**	**GGG**TTTTATGAAAAAGCTAATTATCGCTGCTGCACTTGTTGTTTTCTCGGTGGCT

[a] Orientation was defined in terms of the transposase gene.

[b] NA: not available.

Supplementary Table S4. Prophages identified in the *Pseudomonas* sp. JY-Q genome

Region	Length (kb)	CDS	Region coordinate (bp)		G+C%	*att*L/*att*R
1	40.4	13	874827	915242	53.1	ATTCAGTCCGTTG
2[a]	24.5	31	1586655	1611198	60.9	CCTTGCGTGGTTGAG
3	24.0	25	1612075	1636090	58.9	NA[b]
4	65.6	82	2239657	2305309	60.2	GAACTGGCCGACTAC

a putative degenerated prophage

b NA: not available

Supplementary Table S5. Putative adaptor protein for T6SS effector searching in the *Pseudomonas* sp. JY-Q genome

**Adaptor Name**	**Pfam [a] [Protein families]**	**Homolog locus tag (Note)**	**Reference**
EagR	DUF1795 [PF08786]	AA098_13405 (T6SS-1), AA098_13410 (T6SS-1), AA098_18560 (T6SS-3)	Cianfanelli, *et al*. (2016) *PLoS Pathog*
Tap-1 (TecL)<br>TecC	DUF4123 [PF13503]	AA098_12405, AA098_15255, AA098_16765 (T6SS-2)	Unterweger, *et al*. (2015) *EMBO J*<br>Liang, *et al*. (2015) *Proc. Natl. Acad. Sci. U.S.A*
Atu3641	DUF2169 [PF09937]	-[b]	Miyata, *et al*. (2013) *PLoS Pathog*
PAAR	PAAR [PF05488]	AA098_10970, AA098_11645	Shneider, *et al.* (2013) *Nature*
VgrG	Phage_GPD [PF05954]<br>Phage_base_V [PF04717]	AA098_12410 (Orphan), AA098_13340 (T6SS-1), AA098_13400 (T6SS-1), AA098_15250 (Orphan), AA098_16760 (T6SS-2), AA098_18555 (T6SS-3)	Bondage, *et al*. (2016) *Proc. Natl. Acad. Sci. U.S.A*
Hcp	DUF796 [PF05638]	AA098_13240 (T6SS-1), AA098_16755 (T6SS-2), AA098_18550 (T6SS-3), AA098_20455	Silverman, *et al.* (2013) *Mol Cell*

[a] Pfam ID, protein families database accession number (pfam.xfam.org/)

[b] -: not available

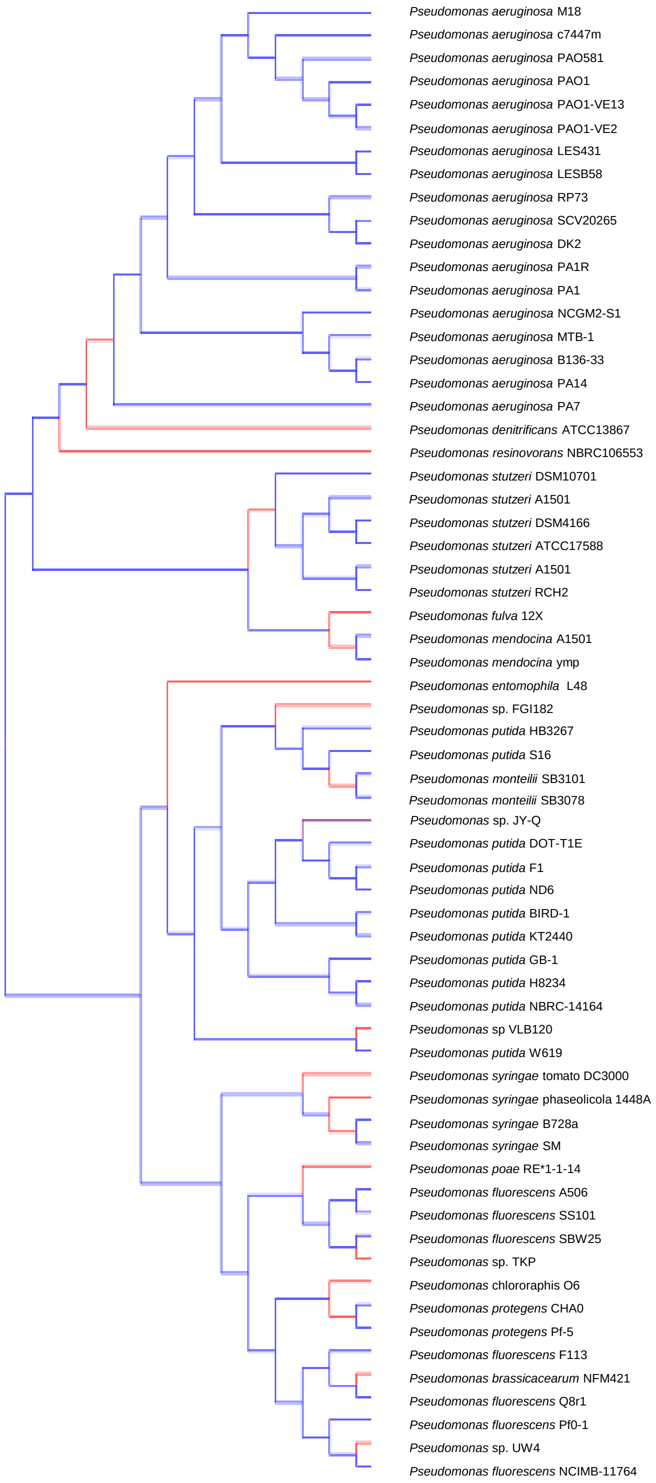

Supplementary Figure S1. Inferred phylogenetic relationships of the 64 sequenced *Pseudomonas* genomes, with the genome of *Pseudomonas aeruginosa* M18 used as the out-group to root the tree. The neighbor-joining tree was generated with CVTree by comparing their proteomes using a composition vector approach (K = 6). *Pseudomonas* isolate names are denoted.

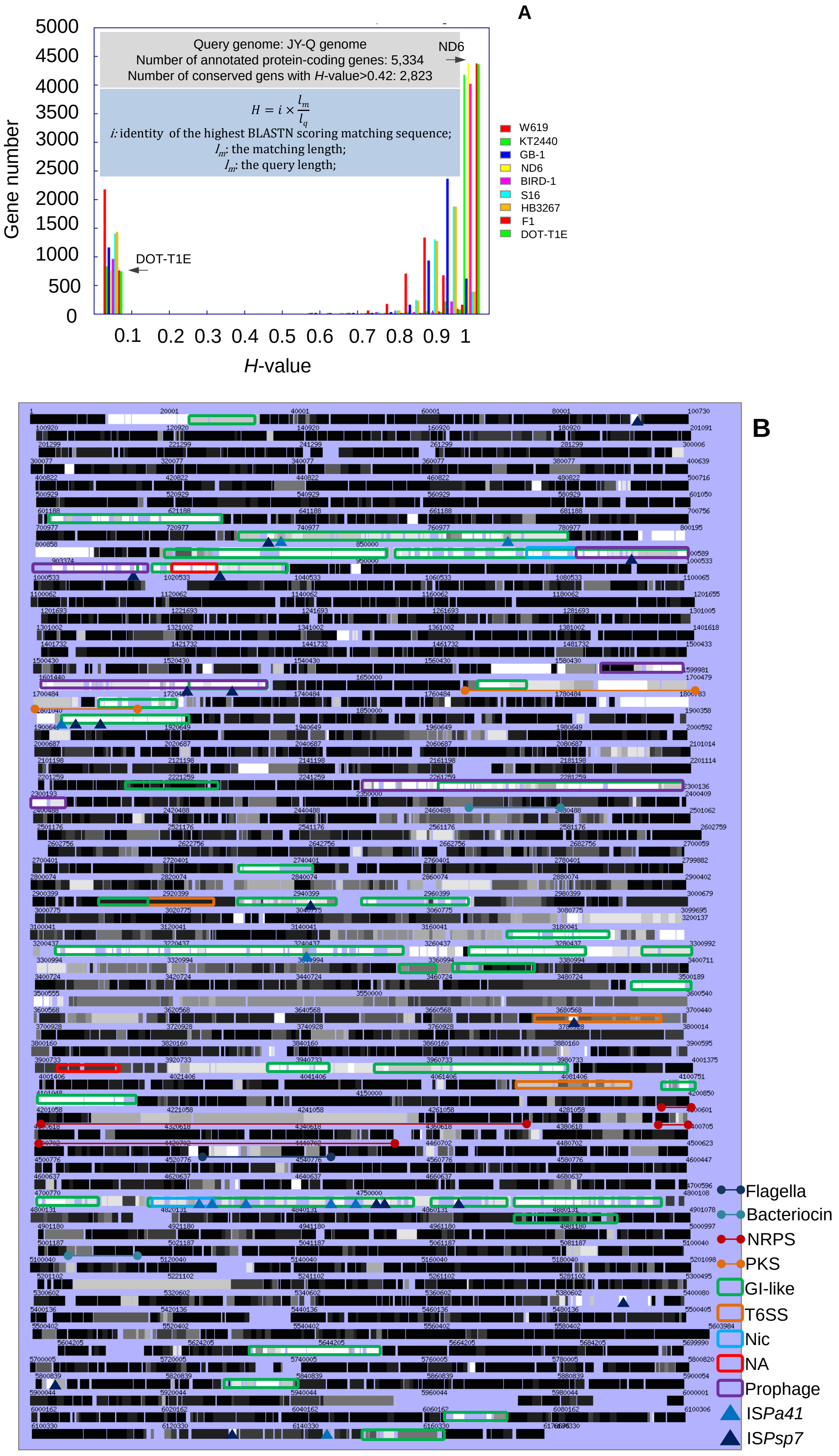
A
Query genome: JY-Q genome
Number of annotated protein-coding genes: 5,334
Number of conserved gens with H-value>0.42: 2,823
H = i × lm/lq
i: identity of the highest BLASTN scoring matching sequence;
lm: the matching length;
lm: the query length;
ND6
DOT-T1E
Gene number
5000
4500
4000
3500
3000
2500
2000
1500
1000
500
0
0.1
0.2
0.3
0.4
0.5
0.6
0.7
0.8
0.9
1
H-value
W619
KT2440
GB-1
ND6
BIRD-1
S16
HB3267
F1
DOT-T1E
B
Flagella
Bacteriocin
NRPS
PKS
GI-like
T6SS
Nic
NA
Prophage
ISPa41
ISPsp7

Supplementary Figure S2. The mGenomeSubtractor-based *in silico* subtractive hybridization of the JY-Q genome against genomes of other completely sequenced *Pseudomonas putida* isolates. (A) Histogram of BLASTn-based *H*-values for all annotated protein-coding genes in the JY-Q chromosome against all 9 subject chromosome sequences (color-coded). The *H*-value reflects the degree of similarity in terms of the length of match and the degree of identity at a nucleotide level between the matching gene in the subject genome and the query gene examined. The conserved genes were identified based on each of the obtained *H*-values great than 0.42. The genome of JY-Q shows the most significant sequence identity to the ND6 genome with 4522 conserved genes (H-value⩾0.42) among all the other 9 completely sequenced genomes. (B) Chromosome map of JY-Q with gene black/white-shade-coded based on the number of comparator *Pseudomonas* genomes identified as harboring a nucleotide sequence-conserved homologue. Genes shown in absolute black ('9') are conserved across all *Pseudomonas* comparator genomes, with genes shown in decreasing shades of black being conserved in lower numbers of *Pseudomonas* comparator genomes, while at the other extreme those shown in white ('0') are unique to JY-Q. Non-coding regions are shown as gaps. The identified genetic elements in this study are separately marked by the symbols in the lower right panel. Note: Nic, nicotine degradation; NA: nicotinic acid degradation; IS, insertion sequence; NRPS, non-ribosomal peptide synthesis; PKS, polyketide synthesis; T6SS, type VI secretion system; GI, genomic island.

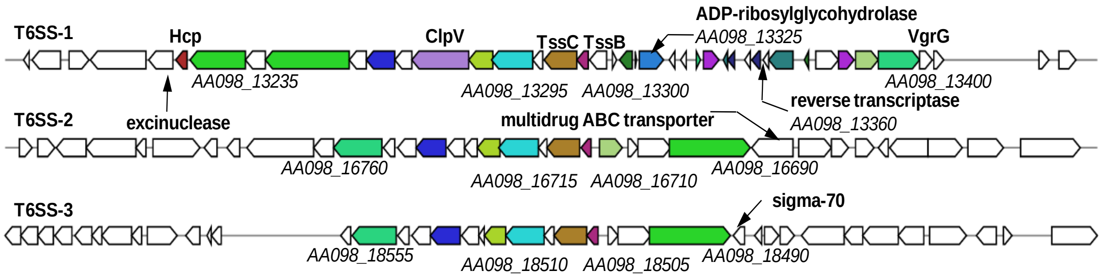

Supplementary Figure S3. Schematic representation of three T6SS gene clusters in *Pseudomonas* sp. JY-Q. The color code of the genes correlates with matching homolog. The observation of remarkably high similar genetic architectures between T6SS-2 and T6SS-3 indicates that T6SS-2 and T6SS-3 could originate from a duplicate event, whereas phylogeny of which T6SS-1 clades separately with an 'intermediated' accessory region.

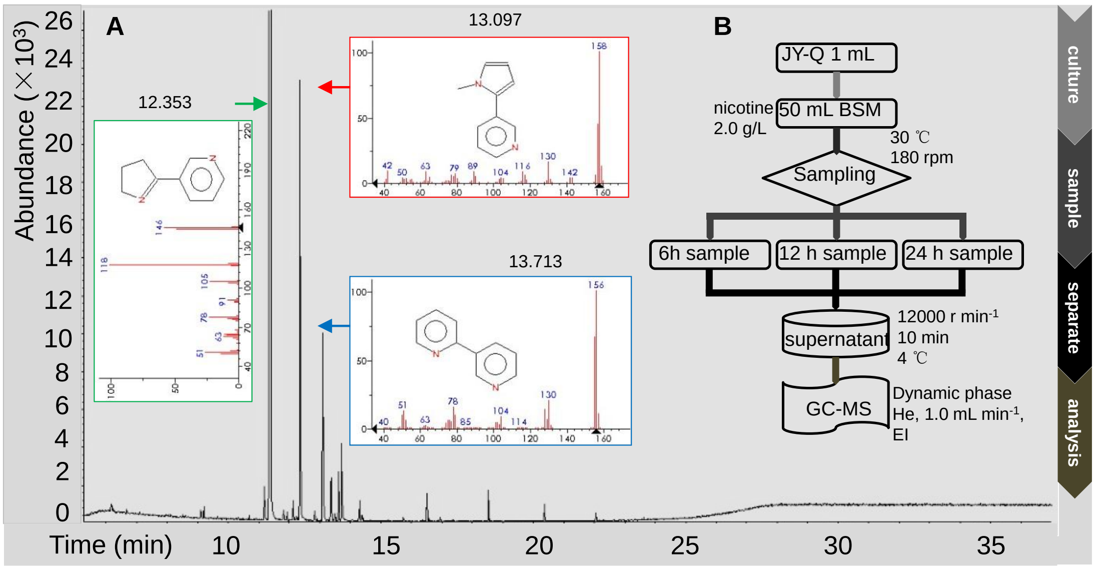

Supplementary Figure S4. Intermediate identification in nicotine degradation of strain JY-Q. (A) GC-MS spectrum and profile of intermediate determination by reaction of *Pseudomonas* sp. JY-Q at pH 7.0 and 37°C using nicotine as the substrate. (B) GC-MS analytical procedures of intermediates for preparation, sampling, cultivation and detection [Details in Materials and methods section]. Chromatographic manipulation conditions were: 1 μL injection volume, carrier gas, helium at a constant flow of 1.0 mL $min^{-1;}$ temperature programme: initial temperature 60 °C and hold for 2 min, then to 280 °C with 10 °C min^{-1} ramp for 10 min.
